# Carbon-Negative Production of Soda Ash: Process Development and Feasibility Evaluation

**DOI:** 10.1021/acs.iecr.5c00483

**Published:** 2025-05-30

**Authors:** Maria F. Gutierrez, Heike Lorenz, Peter Schulze

**Affiliations:** 28307 Max Planck Institute for Dynamics of Complex Technical Systems, Sandtorstr. 1, Magdeburg 39106, Germany

## Abstract

Aiming to produce carbon-negative soda ash, chlor-alkali electrolysis, CO_2_ direct air capture, and sodium carbonate crystallization are combined in a so-called CODA process. In this study, four variants of the CODA process are developed and evaluated by means of modeling and simulation. Variations of the process design are related with the CO_2_ absorption technology, the crystallization strategy, and the possible byproducts of the process. The processes using a cross-flow packed absorber had a smaller CAPEX (between 195 and 209 USD/ton soda) than the process using a droplet absorber (337 USD/ton soda). When coupled with the cross-flow packed absorber, the two-step crystallization strategy had a smaller OPEX (150 USD/ton soda) than the one-step crystallization (175 USD/ton soda). The revenue of selling the process byproducts such as hydrogen, chlorine, and CO_2_ certificates was key to the profitability of the CODA process. The most promising CODA variant (cross-flow packed absorber and two-step crystallization) consumes about 0.15 tons of CO_2_ from the air and earned nearly 200 USD/ton soda ash, making CODA an attractive alternative that deserves to be scaled-up.

## Graphical abstract

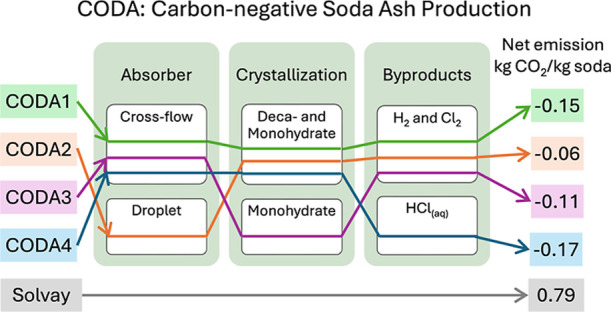

## 1 Introduction

Sodium carbonate is an alkali salt that was obtained in ancient times from the combustion ashes of plants from sodium-rich soils or seaweed (for this reason, it is also known as soda ash). The use of sodium carbonate in the soap and glass manufacture lead to a high demand of this bulk chemical, so that by the end of the 18th century, its large-scale production was enabled by the Leblanc process. The Leblanc process involves the reaction between sodium chloride and sulfuric acid to produce sodium sulfate, which is subsequently reduced into sodium sulfide (see Table 1). This intermediate reacts with calcium carbonate to finally produce sodium carbonate and calcium sulfide. Other byproducts of this process are hydrochloric acid gas and carbon dioxide. In the 19th century, the Leblanc process was replaced by the Solvay process, which was technically and economically superior and less polluting.^1^ In recent years, modifications of the Solvay process have been proposed to reduce the environmental impact of soda ash production,^2^ which are also included in Table 1.

**Table 1:** Reactions Involved in the Most Prominent Routes and Our Proposed Route to Produce Soda Ash

Process	reactions	raw materials	byproducts
Leblanc	2NaCl + H_2_SO_4_ → Na_2_SO_4_ + 2HCl	NaCl, H_2_SO_4_, C, CaCO_3_	HCl, CO_2_, CaS
	Na_2_SO_4_ + 2C → Na_2_S + 2CO_2_		
	Na_2_S + CaCO_3_ → Na_2_CO_3_ + CaS		
Solvay^a^	$CaCO_{3}\overset{C}{\to }CO_{2}+CaO$	NaCl, CaCO_3_, C	CaCl_2_,CO_2_ (from coke combustion)
	2NaCl + 2H_2_O + 2NH_3_ + 2CO_2_ → 2NH_4_Cl + 2NaHCO_3_		
	2NaHCO_3_ → Na_2_CO_3_ + H_2_O + CO_2_		
	CaO + H_2_O → Ca(OH)_2_		
	2NH_4_Cl + Ca(OH)_2_ → 2NH_3_ + CaCl_2_ + 2H_2_O		
modified Solvay (without calcination of limestone – with ammonia consumption)	2NH_3_ + 2CO_2_ + 2H_2_O → 2NH_4_HCO_3_	NaCl, NH_3_, CO_2_, H_2_O	NH_4_Cl
	2NaCl + 2NH_4_HCO_3_ → 2NH_4_Cl + 2NaHCO_3_		
	2NaHCO_3_ → Na_2_CO_3_ + CO_2_ + H_2_O		
e.Solvay^b^,^c^	$2NaCl+2H_{2}O\overset{2e^{-}}{\to }2NaOH+H_{2}+Cl_{2}$	NaCl, CaCO_3_	CaCl_2_
	H_2_+Cl_2_ → 2HCl		
	2HCl + CaCO_3_ → CaCl_2_ + CO_2_ + H_2_O		
	2NaCl + 2CO_2_ + 2NH_3_ + 2H_2_O → 2NaHCO_3_ + 2NH_4_Cl		
	2NaHCO_3_ → Na_2_CO_3_ + CO_2_ + H_2_O		
	2NH_4_Cl + 2NaOH → 2NaCl + 2NH_3_ + 2H_2_O		
CODA^c^	$2NaCl+2H_{2}O\overset{2e^{-}}{\to }2NaOH+H_{2}+Cl_{2}$	NaCl,H_2_O, CO_2_	H_2_, Cl_2_, HCl (optional)
	2NaOH + CO_2_ → Na_2_CO_3_ + H_2_O		
	H_2_ + Cl_2_ → 2HCl (optional)		

a *C*—nonstoichiometric coke combustion for lime kiln heating (C + O_2_ → CO_2_).

b Authors interpretation of the route reported in the video published in.^3^

c *e* ^–^ electricity (preferred renewable).

The Solvay process requires burning of limestone at 1050–1100 °C to liberate CO_2_ that reacts with sodium chloride, ammonia, and water to produce ammonium chloride and sodium bicarbonate (see Table 1). The reaction is carried out with an excess of CO_2_, which is obtained by (nonstoichiometric) coke combustion within the lime kilns and results in the Scope1 emission of 200–300 kg CO_2_ per ton soda ash produced.^4^ The Scope2 emission (Scope1 + electricity and steam, e.g., from a natural gas combined cycle power plant) sums up to 790–1160 kg of CO_2_ per ton of soda ash produced.^5^ The Solvay process cyclically uses ammonia to promote the formation of sodium bicarbonate, which is later transformed in sodium carbonate. The ammonia is recovered by reactive distillation of ammonium chloride and milk of lime (Ca­(OH)_2_), which produces calcium chloride as a byproduct. Due to the low demand of calcium chloride, only a small fraction is used as product and most of the liquid remaining after distillation is discarded with the wastewater. Consequently, approximately 1 t of CaCl_2_ per ton of soda ash is diluted with cooling water and discharged directly into the natural water body (river or sea).

Nowadays soda ash is an important commodity chemical used as raw material for the glass melt and for reacting with sand.^1^ It is also used to neutralize inorganic and organic acids and in the production of sodium salts in the detergent and soap industry. In the production of paper pulp, it is used to react with sulfur dioxide, forming sodium sulfite and sodium bicarbonate. Other uses of soda ash can be found in the iron and steel, food, aluminum, ceramics, textile, and water treatment industries. Nearly 56 million metric tons of soda ash (75% of the total production) are annually worldwide produced by the Solvay process.^4^ As a consequence, around 28 million metric tons of CO_2_ and 56 million metric tons of CaCl_2_ are yearly emitted into the environment.

In order to replace the Solvay process with a more environmentally friendly process, the CODA project (named after carbon-negative sODA ash) aimed to develop a new way to produce soda ash using carbon dioxide from the air and a solution of sodium hydroxide obtained from chlor-alkali electrolysis of purified brine (sodium chloride solution). In contrast with the Leblanc and Solvay processes, the CODA process involves two main reactions (see Table 1) under mild conditions (below 150 °C). The carbonate ion in sodium carbonate is produced in reactive CO_2_ absorption and is subsequently crystallized in a hydrated form of sodium carbonate, which is finally dried to obtain soda ash. By using renewable electricity and a direct air capture (DAC) technology, the new proposed production of soda ash has the potential to avoid the emission of up to 790–1160 kg of CO_2_/t soda (emitted in the Solvay process) and removes around 400 kg of CO_2_/t soda from air (carbon capture and utilization).

The block diagram representing the CODA process is presented in Figure 1. A saturated aqueous brine solution (NaCl 26 wt %) is used as feed in the chlor-alkali electrolysis to produce an aqueous solution of NaOH (32 wt %) required for CO_2_ absorption. Main byproducts of the CODA process are generated in this subprocess: depleted brine (NaCl 18 wt % which is resaturated by evaporation or dissolution in rock salt caverns and recycled), as well as hydrogen and chlorine gases as marketable products. Alternatively, these gases can be combined in a combustion chamber to produce gaseous hydrogen chloride that can be absorbed in water to produce hydrochloric acid (HCl_(aq)_) (dashed lines in Figure 1). The chlor-alkali electrolysis has a high-power consumption (around 2.6 MW-h/t NaOH^6^), and some heat is released. The optional subprocesses for treating hydrogen and chlorine (combustion chamber and HCl absorption) have a big heat release because they are highly exothermic.^7^

**Figure 1:**
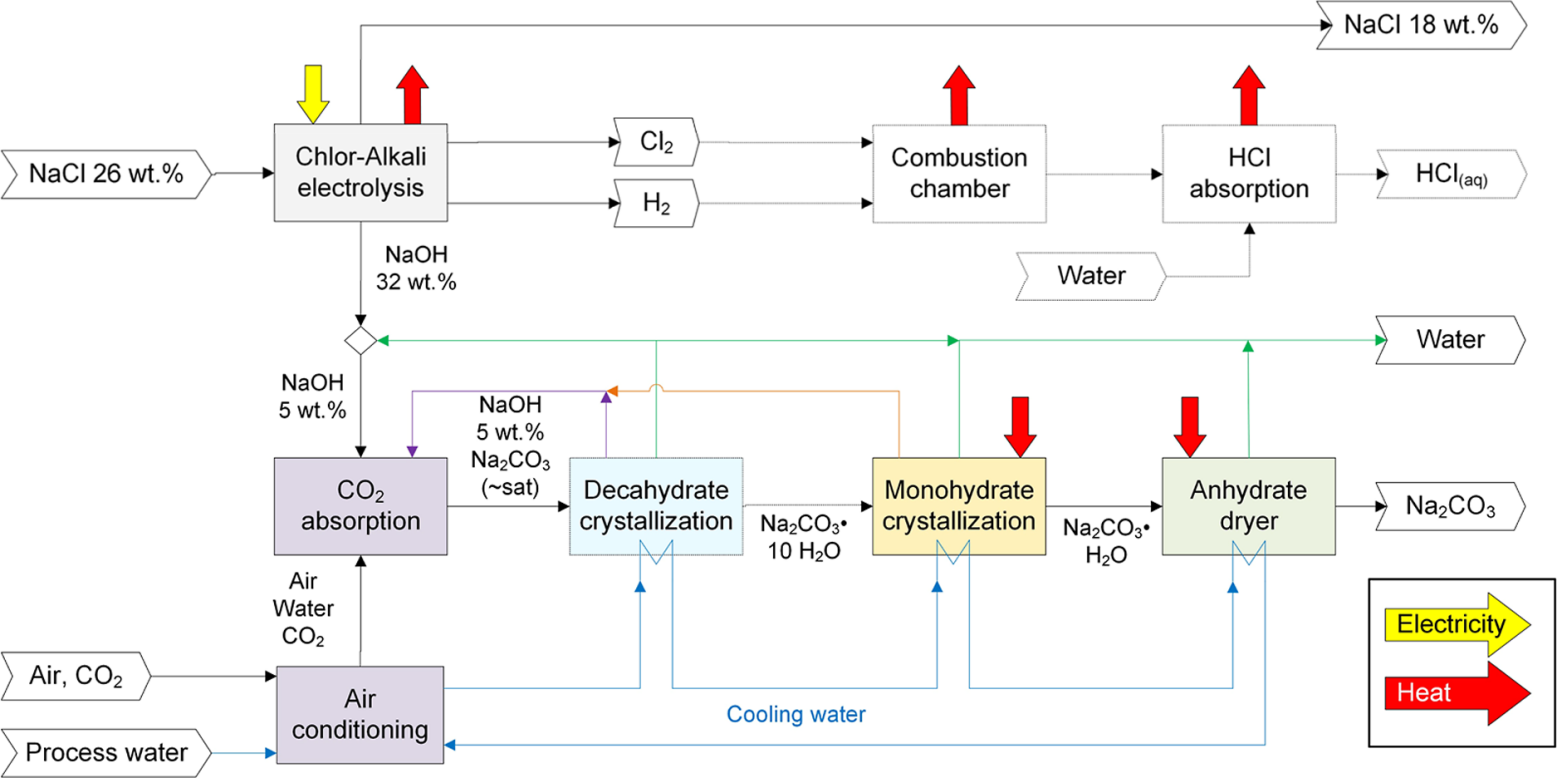
Process block diagram of the CODA process to produce soda ash. Green line: high purity water. Blue line: cooling water. Purple line: recycling stream generated in the process alternative with crystallizing decahydrate. Orange line: recycling stream generated in the process alternative without crystallizing decahydrate. Combustion chamber and HCl absorption are optional subprocesses.

The NaOH solution out of the electrolysis cells is diluted until obtaining a solution with 5–7 wt % NaOH. Previous studies focusing on the fundamentals of the CO_2_ absorption using solutions containing NaOH and Na_2_CO_3_ ^8^ showed that higher NaOH concentrations would result in smaller mass transfer coefficients (less efficient absorption). In the CO_2_ absorption subprocess, previously humidified air is used as feed. This conditioning step allows avoiding highly pure (expensive) water evaporation in the CO_2_ absorber under dry weather conditions and removes pollutants such as dust from the air to be processed. In the conditioning step, process water is evaporated, and as a result, cooling water useful for the rest of the process is produced (blue line in Figure 1). DAC absorption using alkaline solutions has been previously studied using a counter-current packed absorber,^9,10^ a cross-flow packed absorber,^11,12^ and a droplet-based absorber.^13,14^ The effect of the absorber technology on the energy consumption and absorber size has also been studied before.^15^ However, the effect of the choice of absorber type on the global process has not yet been quantified.

The solution from the absorber is used as feed in the crystallization subprocess. The related solid–liquid phase diagram for sodium carbonate in pure water and in aqueous NaOH is presented in Figure 2. Three different hydrates are formed in the range between 5 and 150 °C, and their stable regions are shown in the diagram. The point A in the diagram represents the inlet stream entering the absorber (undersaturated solution at middle European average air temperature). After CO_2_ absorption, the solution is enriched in carbonate ions and its concentration will be closer to point B. The final product of the CODA process is the sodium carbonate anhydrate (Na_2_CO_3_, point F), which is usually obtained by drying (dehydrating) sodium carbonate monohydrate (Na_2_CO_3_·H_2_O, point E) at temperatures of up to 150 °C.^16^ From the phase diagram, two routes to reach point F from point B can be identified. One route comprises two crystallization stages: the first to obtain sodium carbonate decahydrate (Na_2_CO_3_·10H_2_O, point C) at ambient temperature (10 °C in Figure 2) and the second to obtain sodium carbonate monohydrate (Na_2_CO_3_·10H_2_O, point E) at a temperature between 45 and 100 °C (50 °C in Figure 2). In the second route, monohydrate is obtained directly after absorption in one crystallization stage. The crystallization of monohydrate in the first route would require removing less water than that in the second route because the solution fed to this crystallization step would be more concentrated (∼37 wt % in the first route vs ∼8 wt % in the second route). Even though the first route seems more attractive from an energy consumption point of view (operational costs), it also requires more equipment (capital investment costs).

**Figure 2:**
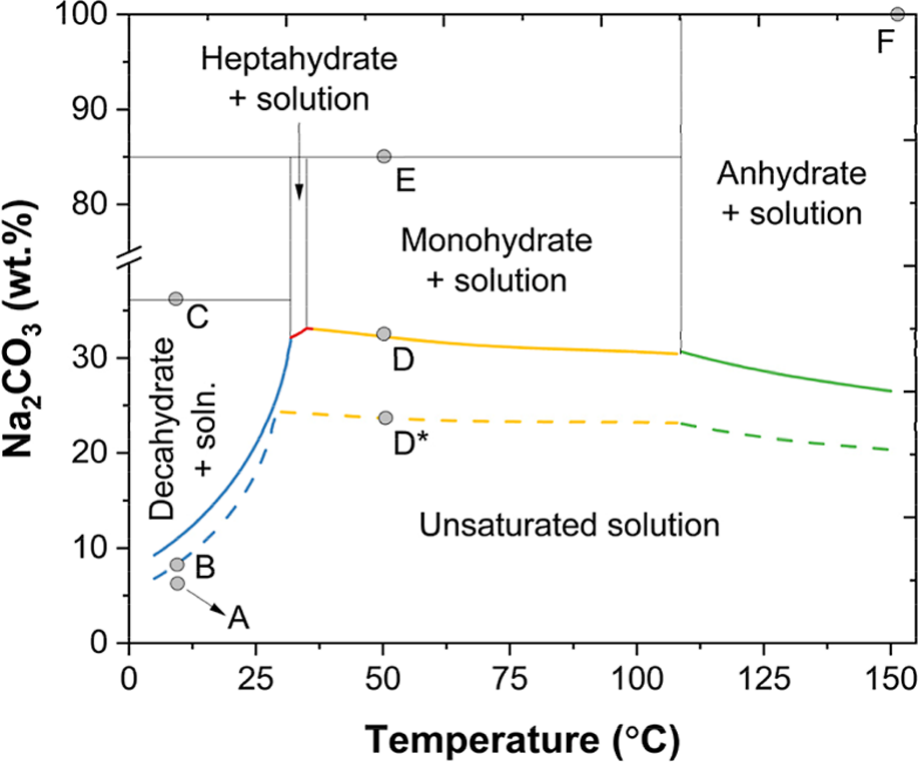
Solid–liquid phase equilibrium diagram of sodium carbonate and its hydrate phases in pure water (continuous lines) and in aqueous 5 wt % NaOH (dashed lines) as a function of temperature. Decahydrate (blue), heptahydrate (red), monohydrate (yellow), anhydrate (green). (A) Undersaturated solution at 10 °C, (B) saturated solution in 5 wt % NaOH at 10 °C, (C) decahydrate crystals at 10 °C, (D) saturated solution in pure water at 50 °C, D*: saturated solution in a 5% NaOH solution at 50 °C, (E) monohydrate crystals at 50 °C, (F): anhydrate crystals at 150 °C. Calculated using ELEC-NRTL parameters and solubility product constants from Aspen Plus V12.^17^

Crystallization of the decahydrate can be achieved either by cooling the solution out of the absorber or by reaching supersaturation in the absorber. The second option requires an absorber technology able to safely deal with potential crystal formation due to supersaturation inside the absorber (droplet absorber), although the absorber would of course be operated in the metastable supersaturation range, in which no spontaneous crystal formation would be expected. First discussions on the implications of the droplet absorption design specifications on the energy consumption of the downstream steps in the CODA process have been previously presented.^14^ In addition, our current studies are focusing on the fundamentals and kinetics of sodium carbonate decahydrate and monohydrate crystallization.^18^

Previous studies on the CODA process allowed us to understand the challenges and requirements of the absorption and crystallization subprocesses. However, a complete assessment of the whole process in terms of energy consumption and technical feasibility has not yet been presented yet. Moreover, the net CO_2_ emission within the CODA process needs to be quantified for the process to be called carbon-negative. In this work, four alternatives of the CODA process are presented and evaluated in terms of energy consumption and the size of the main associated equipment. Based on the available literature and our previous studies, two absorption technologies are compared, and their effect on the downstream stages is discussed. Considering the phase diagram presented in Figure 2, two downstream crystallization strategies are proposed and compared. Heat integration between the upstream (electrolysis and humidification) and downstream processes is also evaluated. In addition, the implications of producing hydrochloric acid for the energy consumption of the CODA process are discussed. Mass and energy balances are performed in Aspen Plus V12,^17^ and the sizing of the equipment is performed using specific models in Python and Excel. Results of this prefeasibility study can be used for economic evaluation, process optimization, and basic/detail engineering of the CODA process, which could finally lead to the implementation of a carbon-negative way to produce soda-ash.

## 2 Process Modeling and Simulation

In order to compare the energy demand and the size of the equipment required in the CODA process, four process scenarios were modeled and simulated. The main differences between the studied process alternatives are summarized in Table 2. In all processes, the chlor-alkali electrolysis and the anhydrate dryer (dehydration of monohydrate) subprocesses (see Figure 1) remain the same. In process P1, a cross-flow packed absorber is used for CO_2_ DAC,^11^ followed by the evaporative vacuum cooling (EVC) crystallization of decahydrate and the evaporative vacuum (EV) crystallization of monohydrate. Process P2 uses a droplet absorber for CO_2_ DAC,^15^ and a supersaturated solution is obtained in its metastable zone^18^ As a result, the following crystallization of decahydrate does not require any cooling. The crystallization of monohydrate in process P2 will be also performed by EV crystallization. Process P3 uses the same absorption technology as P1, but the downstream crystallization strategy goes directly to the EV crystallization of monohydrate (one stage). Finally, process P4 is the only one in which the production of HCl_(aq)_ is considered; other conditions remain the same as process P1. The counter-current absorption technology was not included in the comparison because previous studies showed that the cost of CO_2_ capture of processes using this technology is higher than when using the cross-current absorption (376–400 USD/ton CO_2_ for counter-current absorption^10^ and 94–232 USD/ton CO_2_ for cross-flow absorption^12^).

**Table 2:** Compared CODA Process Variants (or Cases)

subprocess	variations	process			
		P1	P2	P3	P4
absorption	cross-flow packed	X		X	X
	droplet absorber (supersaturated)		X		
crystallization	two stages: crystallization of decahydrate and crystallization of monohydrate	X^a^ ^,^ ^b^	X^c^,^b^		X^a^ ^,^ ^b^
	one stage: crystallization of monohydrate^c^			X^b^	
HCl_(aq)_ production	yes				X
	no	X	X	X	

a Evaporative vacuum cooling (EVC) crystallization of decahydrate.

b Evaporative vacuum (EV) crystallization of monohydrate.

c Supersaturation generated in the absorber.

The modeling and simulation of the process scenarios were done using a variety of computational tools. The calculation of mass and energy balances was performed by using simulation software Aspen Plus V12. The list of components used is given in Table S1 in the Supporting Information. Due to the presence of ions in the mixture, the ELECNRTL property method was used in the simulation. The Elec Wizard tool was used to generate the set of equilibrium, salt formation, and dissociation reactions that could be present in the system (see Table S2 in the Supporting Information). With the aim to activate or deactivate certain reactions in each block, the reactions were conveniently grouped in 4 different sets (see Table S3 in the Supporting Information). Details about the reaction set assignment on each block for all processes are given in the Supporting Information. All processes were simulated to produce 1000 ton/day soda ash from previously purified brine and air. From stoichiometry, such a soda ash plant would capture 0.138 million-ton CO_2_ per year, which is 10% of the total capture proposed in the DAC plant proposed by Carbon Engineering.^12^

### 2.1 Chlor-Alkali Electrolysis

The caustic soda solution used for DAC in the CODA process is produced by electrolysis of brine using a membrane cell technique. The energy released and consumed by the brine electrolysis was of interest to evaluate the heat integration between this upstream process and the subsequent subprocesses. Figure 3 presents the flow diagram of the chlor-alkali electrolysis process and the main associated heat exchangers (HE). Six HE were identified: HE1 and HE2 are used to adjust the feed temperature of the anolyte and catholyte, respectively; HE3 is used to cool down the NaOH enriched solution that is fed to the absorber; HE4 is used to adjust the temperature of the depleted brine; and HE5 and HE6 are used to condense the evaporated water that exists in the cell with the generated H_2_ and Cl_2_, respectively. It would also be conceivable, for example, to combine HE1 and HE4 in one heat exchanger, which was negligible in this work due to the focus on the CODA core process.

**Figure 3:**
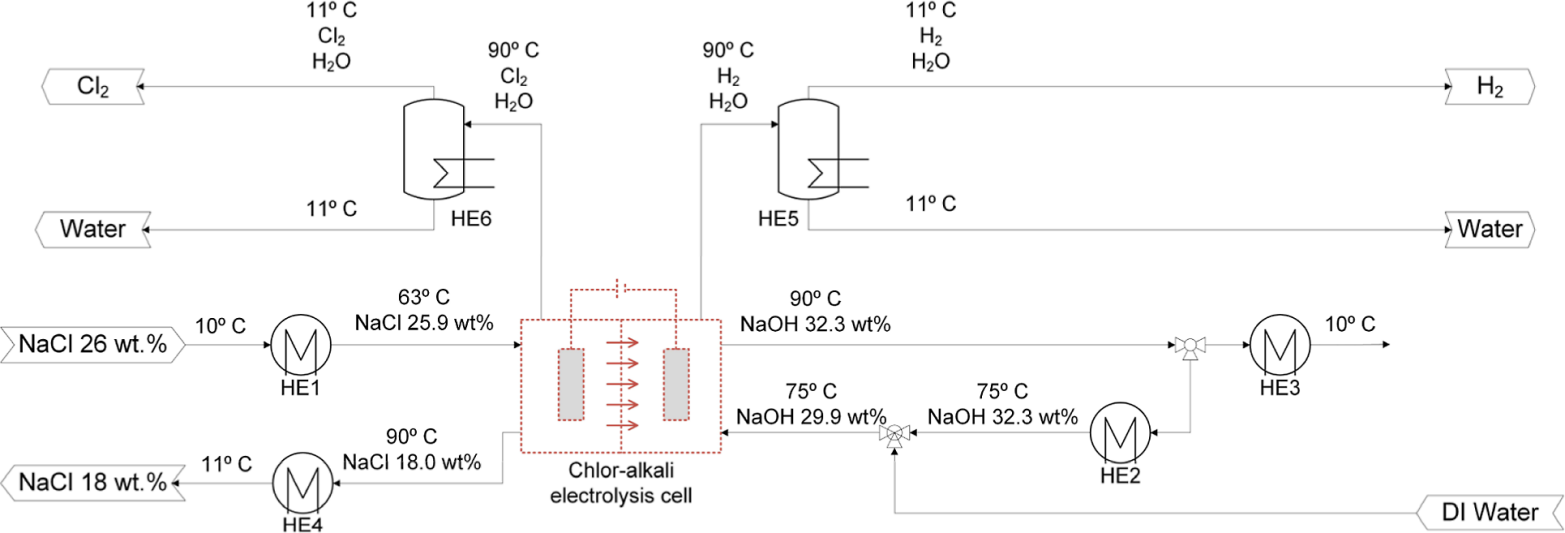
Flow diagram of the chlor-alkali electrolysis process (dashed block not simulated in Aspen Plus V12).

The chlor-alkali electrolysis cell itself was not simulated, but the mass and energy balances around it were simulated in Aspen Plus V12. Correspondingly, the temperature and concentration of the liquid streams entering and exiting the chlor-alkali electrolysis cell were obtained from a previously validated model available in the literature.^19^ The referred model also provided information on the split ratio of alkaline solution that generated the recycled stream entering to HE2. The flows of the streams were determined according to the production capacity, stoichiometry, and concentrations shown in Figure 3 (obtained in the previously mentioned model). The gas streams leaving the electrolysis cell are saturated with water vapor according to the operational temperature. In addition, some of the gases are dissolved in the solution containing ions. The estimation of the concentration and flow of the gases exiting the cell was performed in Aspen Plus V12 by using the *Flash2* model, which considers the gas–liquid phase equilibrium. HE1 to HE4 in Figure 3 was simulated in Aspen Plus V12 using the *Heater* model, while HE5 and HE6 were simulated using the *Flash2* model. The estimation of the electrolysis cell was performed using the model available in the literature,^19^ while the estimation of the area of the HE (*A*) was performed in Python using eq 1. The heat duty (*Q*) was taken from the simulation results, the global heat transfer coefficient (*U*) was defined using heuristic data,^20^ and the mean logarithmic temperature difference (Δ*T*_lm_) was calculated from the simulation results.

1 $$Q=UA\Delta T_{lm}$$

### 2.2 Air Conditioning

With the aim to avoid water condensation or evaporation in the CO_2_ absorber, the present study proposes an air conditioning stage in which the air is humidified before entering the absorber. In a previous study, the water loss in a droplet absorber for DAC was quantified at different conditions of weather (air temperature and humidity) and liquid composition (sodium carbonate concentration).^14^ The water loss can be between 2 and 130 g/kg of CO_2_ captured under dry weather conditions (30% relative humidity), depending on the air temperature and carbonate concentration in the liquid. The evaporation of water in the CO_2_ absorber is undesired because high quality water would be lost (around 18000 ton/year in the worst-case scenario for the proposed process) and because it induces the decrease of the temperature, which could cause crystallization of sodium carbonate decahydrate inside the absorber when working in the metastable supersaturation range. In addition, a desired cleaning effect is assumed in which dust and other impurities are removed from the air, which could otherwise contaminate the product.

The humidification of air uses process or cooling water (e.g., surface water which is cheaper than high-quality deionized water required for the crystallizers) and causes the cooling of the water by evaporation. In this study, the use of the cooled process water to fulfill the cooling demand of the process is evaluated (see Figures 1 and 4; HE16). It is proposed to use a cross-flow packed absorber for all process variants (P1–P4), which is the same technology used for the CO_2_ absorption in P1, P2, and P4. In order to estimate the mass flow of water required in the humidification and outlet air temperature, this unit operation was simulated using a *Radfrac* model in Aspen Plus V12. The main specifications of the simulation of this subprocess are summarized in Table 3. The target humidity of the outlet air in the humidification was set according to the simulation of the CO_2_ absorber, in which the humidity of the inlet air (outlet of humidification) was changed to ensure that there was no water evaporation to the air or water condensation from the air.

**Figure 4:**
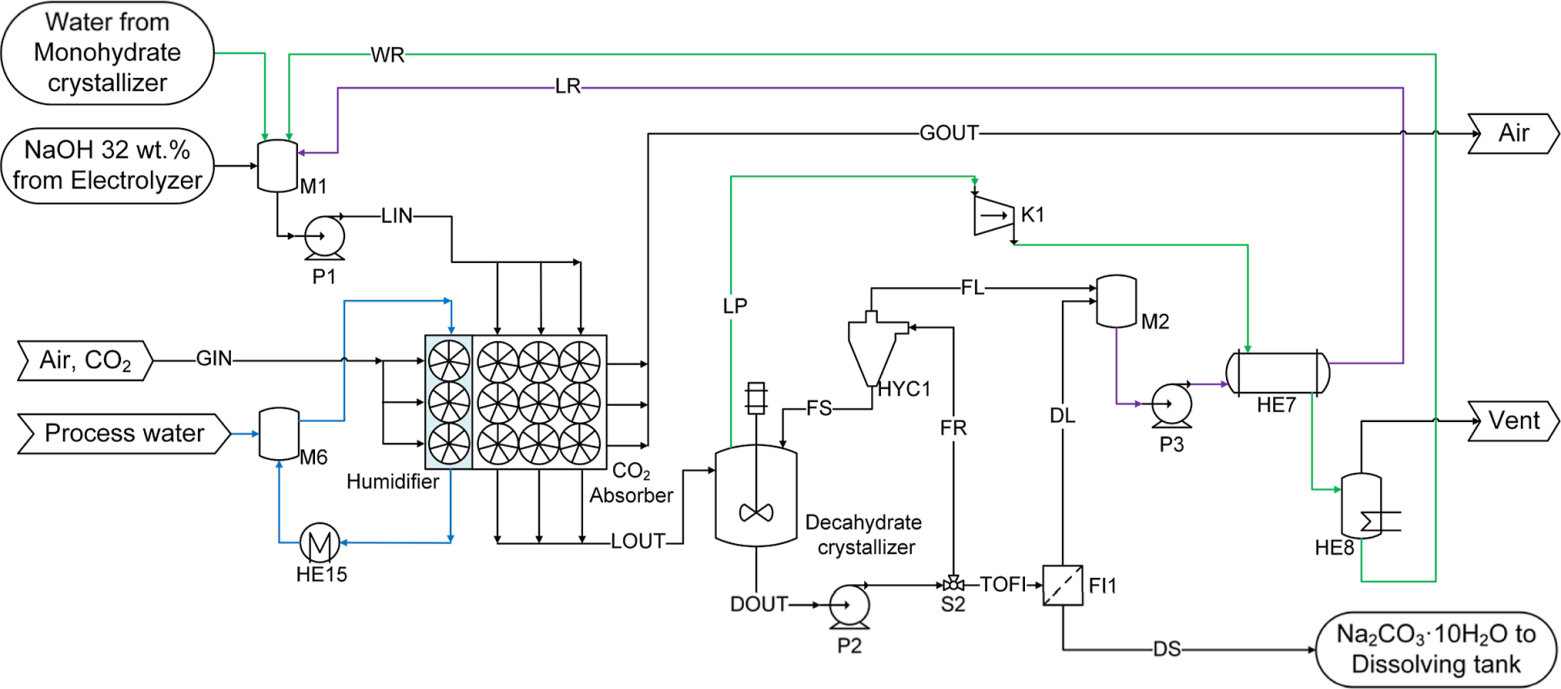
Flow diagram of the absorption-decahydrate crystallization loop used in processes P1 and P4. Green line: high purity water. Blue line: cooling water. Purple line: recycling stream.

**Table 3:** Specifications for the Aspen Plus V12 Simulation of the Air Conditioning

calculation type	rate-based
number of stages	2
top pressure	1 bar
packing type	Mellapack 250X
diameter	calculated with the interactive sizing tool of Aspen Plus V12
packed height per stage (HETP)	varied to obtain the air outlet temperature (design specification)
mass transfer coefficient method	Brf-85
interfacial area method	Brf-85
heat transfer coefficient method	Chilton and Colburn
water inlet temperature	11 °C
water inlet mass flow rate	varied to obtain the air outlet humidity (design specification)
air inlet temperature^a^	10 °C
air outlet temperature (target)	10 °C
air inlet humidity*	80%RH
air outlet humidity^b^ (target)	P1:93.94%RH
	P2:91.78%RH
	P3:97.88%RH
	P4:93.40%RH

a Average weather conditions in Magdeburg (Deutscher Wetterdienst). RH: relative humidity.

b Calculated in CO_2_ absorption simulation to avoid condensation or evaporation of water in the CO_2_ absorber.

As presented in Table 3, two design specifications were used in Aspen Plus V12 to achieve the air conditioning goals: the flow of water was varied to set the air temperature constant (outlet temperature same as inlet temperature), and the size of the equipment was varied to set the outlet air humidity in the desired value. As a result, the humidification subprocess allows us to control the absorption temperature in all processes (P1–P4) and the crystallization of decahydrate in the processes P1, P2, and P4.

The type of packing used in the simulation (Mellapack 250X) was used in previous studies of the CO_2_ absorption using counter-flow arrangement.^10,11^ However, the flow arrangement proposed for the humidification subprocess in this study is cross-flow and the type of packing is Brentwood XF12560 (like in^11,12^ ) because of its lower air pressure drop and cost. Consequently, the calculation of the equipment size done by Aspen Plus V12 through the design specification does not correspond to the real size required for the cross-flow packed.

A better estimation of the packing depth for humidification (*Z*_h_) was performed with eq 2, which is obtained from the mass and energy balances on a humidification tower.^21^ The air velocity (v_G_), the overall mass transfer coefficient (*K*_G,h_), and specific surface area (*a*) are required to calculate the theoretical height of transfer unit (*H*_tOG_). The air velocity and the specific surface area were set according to the values reported for the cross-current flow packed absorber proposed by Holmes in the optimized base case.^11^ Since the mass transfer correlation and geometry of Mellapack 250X were used to calculate *K*_G,h_, the calculated packing depth is not completely accurate but serves as a basis for the comparison analysis.

2 $$Z_{h}=H_{tOG}N_{tOG}=\frac{v_{G}}{K_{G,h}a}\int_{H_{in}^{\prime }}^{H_{out}^{\prime }}\frac{dH^{\prime }}{\left(H^{\prime *}-H^{\prime }\right)}$$

The number of transfer units (*N*_tOG_) was calculated by the integral of a function of the enthalpy of the air–water mixture along the packing (*H* ^′^) and the enthalpy of the air–water mixture under the saturation conditions (*H* ^′*^). The inlet (*H*_in_^′^) and outlet (*H*_out_^′^) enthalpies of the air–water mixture, which are the integral limits, are calculated from the absolute humidity and temperature of the mixture at the inlet and outlet of the packing. The enthalpy of the air–water mixture along the packing is calculated from the operational curve, which depends on the gas to liquid ratio. Details on the calculation of each term are provided in section B of the Supporting Information.

### 2.3 CO_2_ Absorption

Due to the lack of models in Aspen Plus V12 able to describe the two types of absorption technologies selected (droplet absorber and crossflow packed absorber), the sizing of the CO_2_ absorption equipment was performed outside Aspen Plus V12 and using information from the literature.^11,14,15,22^ However, in order to improve the calculation of the recycling streams in the processes, this unit operation was also simulated in Aspen Plus V12 using a *Radfrac* model and was adjusted to fit the real equipment performance. The main specifications of the simulation of this subprocess are summarized in Table 4.

**Table 4:** Specifications for the Aspen Plus V12 Simulation of the CO_2_ Absorption

calculation type	rate-based
number of stages	2
top pressure	1 bar
packing type	Mellapack 250X
	(P2: Ceramic Rasching Rings)
diameter	calculated with the interactive sizing tool of Aspen Plus V12
packed height per stage (HETP)	varied to obtain the capture efficiency (design specification)
mass transfer coefficient method	Brf-92(P2: Onda-68)
interfacial area method	Brf-92(P2: Onda-68)
heat transfer coefficient method	Chilton and Colburn
inlet CO_2_ mole fraction (mass fraction)	400 ppm (∼0.0613 wt %)
air inlet temperature	10 °C
air inlet humidity	varied to avoid condensation or evaporation of water in the absorber
gas to liquid mass ratio, *G*_m_/*L*_m_	2.161(P2:0.067)
capture efficiency, η_abs_(target)	74.2%(P2:42.1%)

The type of packing used in the CO_2_ absorption simulation was selected to match the gas to liquid ratio required in each selected technology (cross-flow packed and droplet absorber). The gas to liquid mass ratio (*G*_m_/*L*_m_) and the capture efficiency (η_abs_), which are also reported in Table 4, were obtained from the literature (^11^ for the cross-flow packed absorber and^13^ for the droplet absorber).

The calculation of the total mass inlet flow of air to the process ( $\dot{G_{in}}$ ) was calculated based on the CO_2_ absorption requirement ( $\dot{F_{CO_{2}}}$ ), the capture efficiency of each absorption technology, and the inlet CO_2_ mass fraction ( $y_{CO_{2}}$ ), as presented in eq 3. According to the reactions in the CODA process (see Table 1), it is required to absorb 17301.29 kg/h of CO_2_ to produce 1000 ton/day of soda ash. The liquid inlet mass flow to the absorber ( $\dot{L_{in}}$ ) was calculated based on the gas to liquid ratio of each technology, as presented in eq 4.

3 $$\dot{G_{in}}=\frac{\dot{F_{CO_{2}}}}{y_{CO_{2}}\eta_{abs}}$$

4 $$\dot{L_{in}}=\frac{\dot{G_{in}}}{G_{m}/L_{m}}$$

The estimation of volume of the cross-flow packed absorber (*V*_abs_) was based on the optimized base case design proposed in the literature,^11^ in which the air velocity was set in 1.6 m/s, the packing depth (*D*) in 8.6 m, and the specific air pressure drop in 20.23 Pa/m. With these conditions, the flow of CO_2_ captured per inlet area ( ${\mathrm{r̂}}_{abs}$ ) is 3.12 kg/(h-m^2^). The expression used to calculate the air-contact or volume is listed in eq 5. The height of the absorber was fixed in 20 m, as in^11^ and the length of the absorber was calculated from the volume.

5 $$V_{abs}=\frac{\dot{F_{CO_{2}}}}{{\mathrm{r̂}}_{abs}}D$$

On the other hand, the estimation of the droplet absorber volume (*V*_abs_) was based the computer-aided assessment of a droplet absorber presented in a previous publication.^13^ The volume of the droplet absorber studied in the mentioned work was 0.00319 m^3^ (5 m height, 28.5 mm diameter), and the nozzle plate pressure drop was 11426.8 Pa. Since the fall of droplets drives the air flow in the droplet absorber, the liquid and air velocities are tightly related. The droplets are generated by a nozzle plate with 841 holes of 180 μm diameter, in which a liquid volumetric flow rate of 2.63 L/min should be handled to ensure the proper droplet formation regime (vertical injection, no spray). Through a CFD simulation, the average *G*_m_/*L*_m_ and the specific surface area in the droplet absorber were estimated. This information was used to feed the differential material balances in the absorber, which allowed to calculate η_abs_. More details on the model and its validation with experimental data can be found in a previous publication.^15^

In the present study, the droplet absorber consists of a certain number of small absorbers (*n*_abs_), each with 5 m height, 28.5 mm diameter, and a nozzle plate as head. The number of small absorbers is calculated from the total mass liquid flow (previously calculated with eq 4), the liquid density (ρ_L_), and the liquid volumetric flow rate required in each nozzle plate ( ${\mathrm{q̇}}_{i}=2.63L/\min$ ), as presented in eq 6. The total droplet absorber volume is calculated with eq 7, in which the number of small absorbers and the volume of each small absorber (*V*_*i*_ = 0.00319 m^3^) are required.

6 $$n_{abs}=\frac{\dot{L_{in}}}{\rho_{L}{\mathrm{q̇}}_{i}}$$

7 $$V_{abs}=n_{abs}V_{i}$$

Even though the dimensioning of the absorber (or humidifier) performed in Aspen Plus V12 does not represent the real size of the proposed equipment, the dimensions should be given when the rate-based calculation type is used in Aspen Plus V12. As shown in Tables 3 and 4, the interactive sizing tool was used to estimate the diameter of the absorber (or humidifier). However, due to the high inlet mass flow rate of air and liquid used in the simulations, the estimated diameter exceeded the limits of the usual equipment in Aspen Plus V12 (30 m). In order to obtain a successful hydraulic evaluation and sizing of the equipment, it was necessary to split the inlet flow rate of liquid and air into 5 smaller streams each.

As mentioned before, the humidity of the inlet air was varied to ensure that there was no condensation or no evaporation of water in the absorber. This was done manually by copying and replacing the outlet flow of water in the air as an inlet specification until no change in the outlet flow of water in the air was observed.

### 2.4 Crystallization

The liquid stream from the absorber is fed into a crystallizer in which either Na_2_CO_3_·10H_2_O (decahydrate) or Na_2_CO_3_·H_2_O (monohydrate) is crystallized, depending on the process variant/case (see Table 2). This unit operation was simulated in Aspen Plus V12 by using a *Crystallizer* model. The main specifications of the simulation of this subprocess are summarized in Table 5. In the process variants where the decahydrate is crystallized first (P1, P2, and P4), the resulting crystals are then partly redissolved in their own crystal water before being fed as suspension to the second crystallizer to obtain the monohydrate crystals. This is done in a dissolving tank, which is simulated in Aspen Plus V12 also using the *Crystallizer* model, as presented in Table 5.

**Table 5:** Specifications for the Aspen Plus V12 Simulation of the Crystallization

specification	process	decahydrate crystallizer	monohydrate crystallizer	dissolving tank
operating conditions (degrees of freedom of the crystallizer model in Aspen Plus V12, fixed during the simulation to allow convergence)	P1	temperature: 9.236 °C	temperature: 50 °C	temperature: 50 °C
		vapor flow rate: 46332.5 kg/h	heat duty: 38390.7 kW	pressure: 1 bar
	P2	temperature: 10.012 °C	temperature: 50 °C	temperature: 50 °C
		pressure: 1 bar	heat duty: 38389.2 kW	pressure: 1 bar
	P3		temperature: 50 °C	
			product flow rate: 48748.9 kg/h	
	P4	temperature: 9.241 °C	temperature: 50 °C	temperature: 50 °C
		vapor flow rate: 46362.9 kg/h	heat duty: 14982.6 kW	pressure: 1 bar
convergence: tear method	all	Broyden	Wegstein	Broyden
recirculation flow rate	P1, P2, P4		188158 kg/h	
	P3		2213860 kg/h	
salt component ID	all	DECA(S)	MONO(S)	DECA(S)
valid phases	all	vapor–liquid	vapor–liquid	liquid-only
operating mode	all	crystallizing	crystallizing	dissolving or melting
particle size distribution (PSD)	all	user-specified PSD: log–normal distribution function *d*_50_720 ± 1μm	user-specified PSD: log–normal distribution function *d*_50_812 ± 1μm	copy PSD from inlet
crystal mass fraction in the suspension, *w*_k_	all	0.20	0.20	0.0916

The specification of the operating conditions presented in Table 5 was conveniently performed to ensure block convergence when all the recycling streams were connected. For the decahydrate crystallizer, the temperature was defined to obtain an adiabatic crystallizer (zero heat duty). For the processes P1 and P4, the supersaturation required for crystallization was obtained by the cooling of the mixture, which was achieved through vacuum water evaporation (EVC). With the aim to obtain a completely electrified plant, the produced vacuum was compressed by compressor K1 to increase its temperature and to use it for heating the recycling stream to the absorber (widely known as mechanical vapor recompression, MVR). This scheme is presented in Figure 4, where a hydrocyclone (HYC1) and a filter (FI1) were included to separate the crystals (FS and DS) from the saturated liquid (FL and DL). The liquid streams obtained from these separators are recycled and mixed in the mixing tank M1 with the NaOH solution coming from the electrolysis (purple line) and recycled water streams. The suspension exiting the crystallizer (DOUT) is divided into two streams (by splitter S2): one to feed the filter (TOFI) and the other to feed the hydrocyclone (FR). The splitting factor was defined to obtain the desired flow rate of crystals out of the loop (112489 kg/h decahydrate DS). The vapor flow rate (LP) was selected to ensure a proper suspension density in the crystallizer with a weight fraction of solids of around 20%. The vacuum pressure required in the crystallizer was calculated as a result of the simulation. As presented in the process flow diagram, all the evaporated water is recycled after condensing and cooling (in HE7 and HE8). Since some water is lost in the vent stream, which also contains incondensable gases that were solubilized in the absorption, a makeup stream of water, coming from the monohydrate crystallization, is added to fulfill the water balance in the loop. The pressure increase in the compressor (K1) was defined by using a design specification to obtain a minimum temperature approach in the heat exchanger (HE7) of 5 °C.

The flow diagram for the absorber-decahydrate crystallization loop for process P2 is given in Figure 5. Since in the process variant P2, the supersaturation is achieved in the absorber, no cooling of the mixture is required for decahydrate crystallization. However, the absorption of CO_2_ and its reaction with NaOH are exothermic processes,^23^ and the temperature of the mixture is slightly increased (from 10.040 to 10.044 °C). As presented in Figure 5, this energy should be removed from the stream before it enters the crystallizer (in HE7) at 10 °C so that the crystallizer has zero heat duty in the simulation and the crystallization is only driven by the supersaturation generated in the absorber.

**Figure 5:**
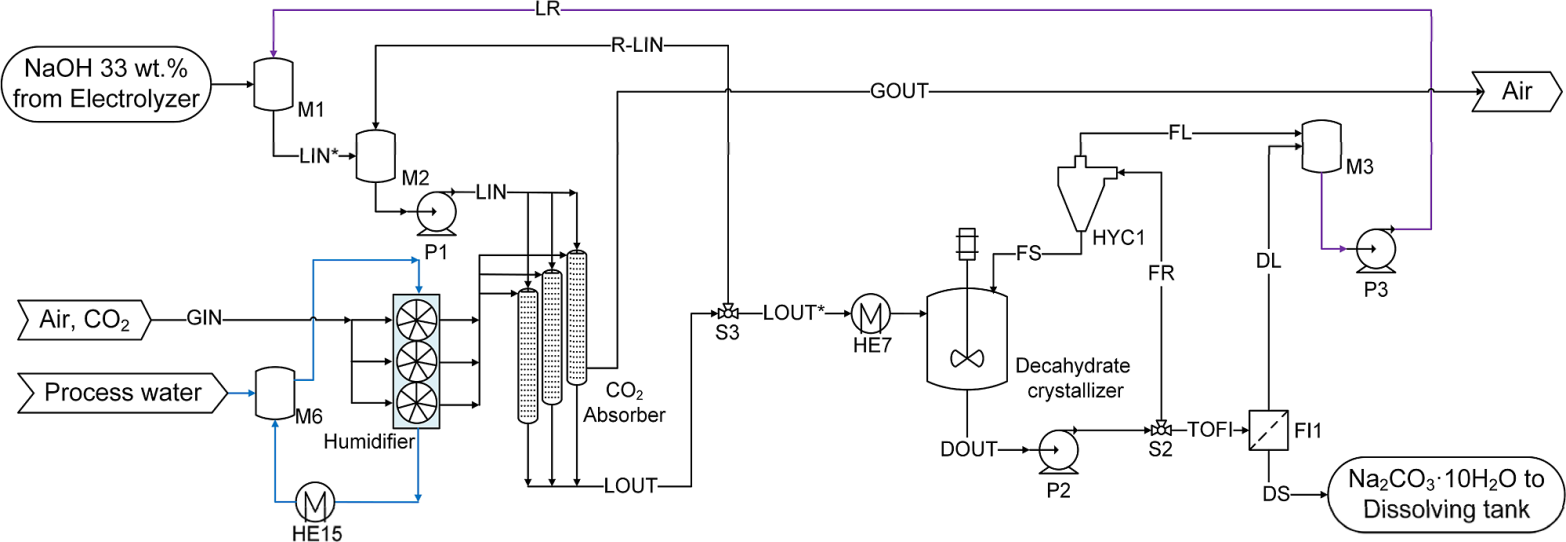
Flow diagram of the absorption-decahydrate crystallization loop used in process P2. Blue line: cooling water. Purple line: recycling stream.

In this process variant, no water makeup is required and the inlet concentration of the NaOH solution from the electrolyzer is slightly higher than that for the P1 and P4 processes previously presented. Due to the very small gas to liquid ratio required in the droplet absorber (see Table 4) and with the aim to reduce the size of the subsequent crystallization-related equipment and its associated energy consumption, an internal recycling of liquid in the absorber is defined in the S3 splitter. The internal splitting factor was arbitrarily selected with the aim of recycling 70% of the total liquid mass out of the absorber. However, it should be noted that supersaturation in the absorber is controlled by this recycle stream. Thus, in a real plant, it would be adjusted to the needs of the crystallizer. As in processes P1 and P4, the splitting factor in S2 was defined to obtain the desired flow rate of crystals out of the loop. In this case, the crystallizer pressure was specified, and no external design specification was needed to ensure the desired suspension density.

For processes P1, P2, and P4, the decahydrate crystals are fed to a dissolving tank in which the temperature is increased to 50 °C. The simulation of this equipment was done as presented in Table 5. The heat required for this subprocess is transferred in a heat exchanger HE9 in a forced circulation arrangement, as shown in Figure 6. The internal recirculation flow rate (splitter S4) was defined by using a design specification to obtain zero heat duty in the dissolving tank itself (*Crystallizer* block in Aspen Plus).

**Figure 6:**
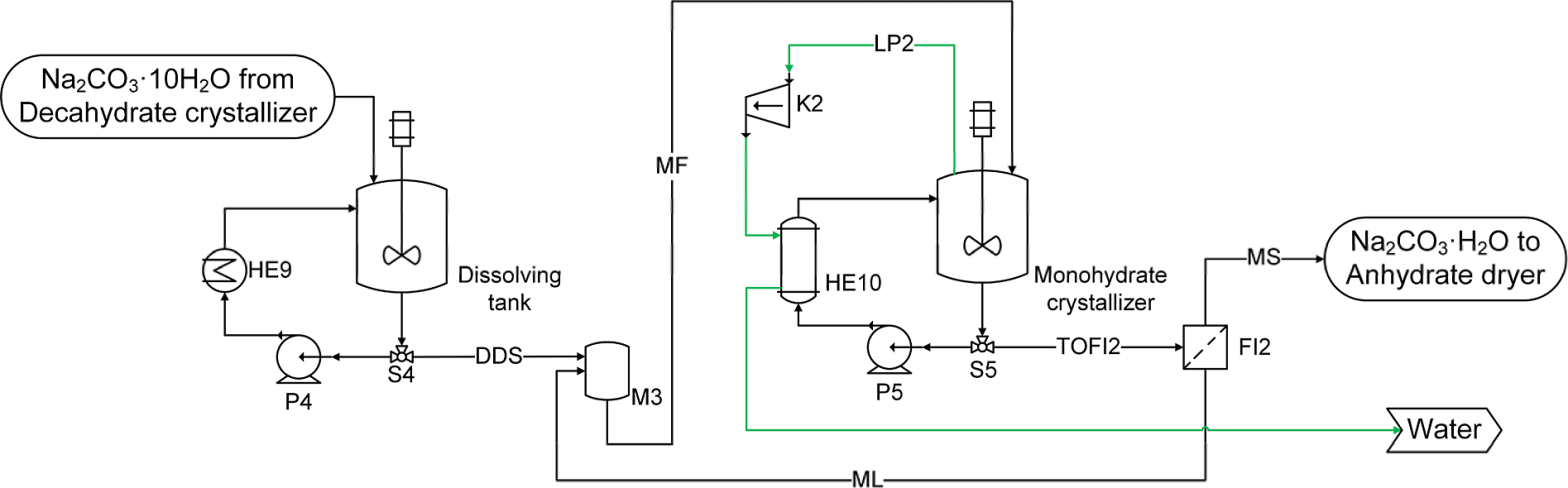
Flow diagram of the dissolving tank and monohydrate crystallizer loops used in processes P1, P2, and P3. Green line: high purity water. Red line: steam used to provide the drying heat.

The supersaturation required for monohydrate crystallization is obtained by water evaporation under vacuum conditions (EV), as presented in Figure 6. The vapor produced (LP2) is compressed (and consequently heated) to provide the temperature level required in the crystallizer and used in the heat exchanger HE10 as utility fluid for the evaporation (in a mechanical vapor recompression scheme, MVR). The pressure ratio in the compressor (K2) was defined with a design specification to achieve a minimum temperature approach in the heat exchanger (HE10) of 5 °C. The monohydrate crystallizer model was defined as compiled in Table 5. For processes P1, P2, and P4, the temperature and heat duty were specified, and consequently, the vapor flow rate and the vacuum pressure required were calculated by the simulation. The heat duty was defined using a design specification to achieve the target product crystal flow rate (48748.89 kg/h of monohydrate). In this crystallizer, the recirculation flow rate (generated in splitter S5) was specified inside the block so that Aspen Plus V12 creates and converges the tear stream internally. This recirculation flow rate was estimated in an additional simulation (not shown here), in which a design specification was used to obtain zero heat duty in the crystallizer block.

For the process variant P3, the solution out of the absorber is heated to the temperature of the monohydrate crystallization and fed to the crystallizer, as presented in Figure 7. For this case, an internal recycling of the liquid in the absorber is also added (generated in splitter S3) but in this case, the reason is to generate the desired suspension density in the simulation. As in the other processes, the recirculation flow rate of the monohydrate crystallizer (generated in splitter S5) was estimated in an additional simulation (not shown here). Some of the evaporated water in the crystallizer is recycled and mixed with the mother liquor obtained from the crystallization (orange line) and the sodium hydroxide solution coming from the electrolysis. The flow rate of the recycled water (generated in splitter S6) was defined to fulfill the water mass balance in the loop. In contrast to the other processes, in process P3 the product crystal flow rate was specified directly in the *Crystallizer* block and the heat duty was calculated as a result (see Table 5). This specification helped for the convergence of the absorber-crystallizer loop.

**Figure 7:**
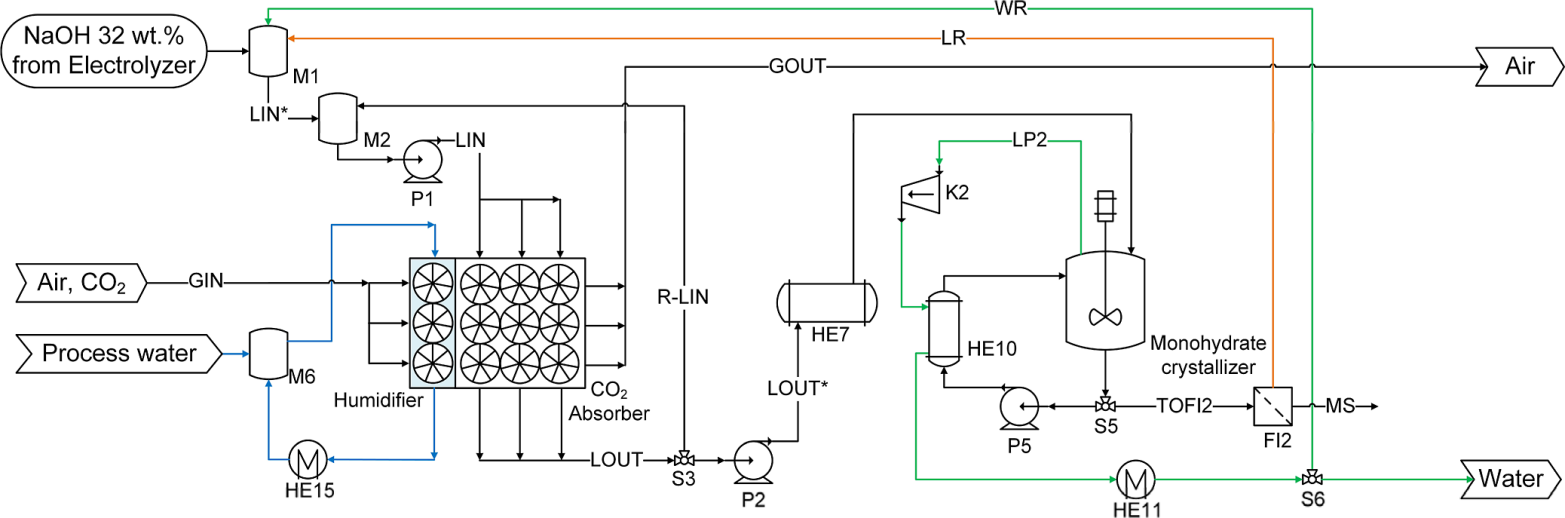
Flow diagram of the absorption-monohydrate crystallization loop used in process variant P3. Blue lines: cooling water. Orange lines: recycling stream. Green lines: steam and water condensate. Black lines: main process streams (solutions and suspension).

In all crystallizers, the set of reactions assigned through the Chemistry ID and the thermodynamic model (ELECNRTL) were used to calculate the saturation concentration. This model was validated using experimental data from the literature and from our experiments (see section C in the Supporting Information). For the simulation in Aspen Plus V12, the final size of the formed crystals was specified according to our experimental results.^24^ The experimental conditions were tuned to yield crystals with a quality according to dense soda ash (bulk density ∼1100 kg/m^3^ and mean crystal size around 800 μm), which has the major market share of soda ash products.

### 2.5 Anhydrate Dryer

The drying of monohydrate crystals is a common subprocess in all the proposed process variants to produce the anhydrous soda or soda ash. Therefore, this unit operation was not sized or modeled in detail. For the simulation in Aspen Plus V12, a *RStoic* reactor was used and the chemical reaction in eq 8 was specified with 100% conversion. The temperature of the reactor was set to 150 °C following the conditions of the conventional calciners to produce soda ash.^16^

8 $$Na_{2}CO_{3}\cdot H_{2}O\to Na_{2}CO_{3}+H_{2}O$$

For processes P1, P2, and P3, the energy required for the drying is given by superheated steam (direct convective dryer), which is produced by compression of the steam removed in the dryer, as illustrated in Figure 8. This concept of superheated steam drying is well-known in the literature^25^ and has the advantage of being powered solely by renewable electricity using an open-loop heat pump principle, similar to the mechanical vapor recompression used in the crystallizers in this work. The vapor from the dryer is divided (S6) into two parts (LP3–1 and LP3–2), which are fed into two compressors working at different levels of pressure. The high-pressure compressor (K3) produces high-temperature steam (HP3–1) that is used to superheat the second part of the steam, which is continuously recycled through the dryer to provide the sensible heat required for drying. The outlet pressure of the low-pressure compressor (K4) was defined using a design specification to ensure that the temperature of the steam entering back into the dryer (HS3–2) was 200 °C. A second design specification was used to establish the flow of steam used in the high-pressure compressor (splitting factor in S6) to obtain zero heat duty in the dryer. Additional water was added to the feed of the high-pressure compressor to avoid temperatures above 212 °C.^20^

**Figure 8:**
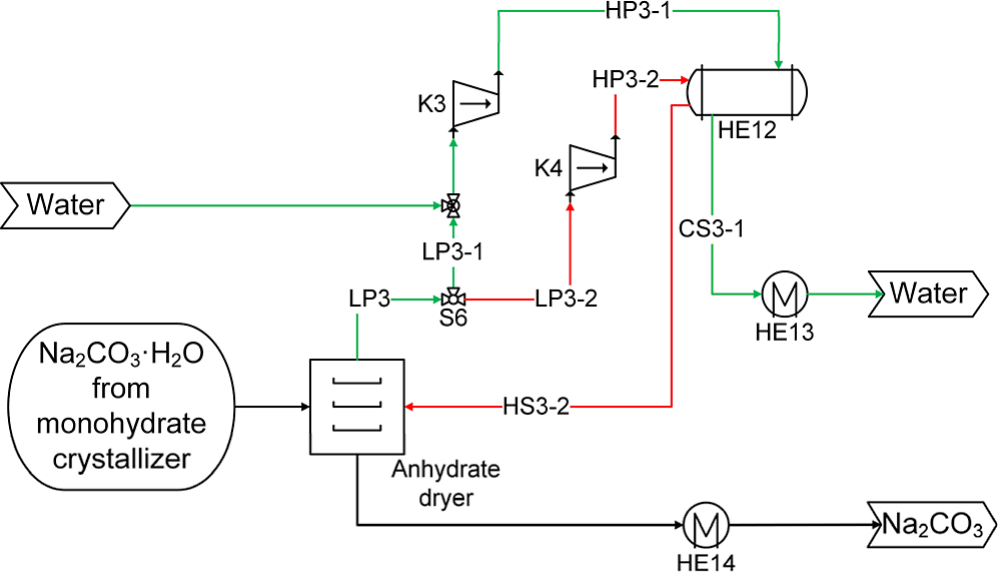
Flow diagram of the steam dryer for the dehydration of monohydrate crystals to produce soda ash (anhydrate) used in processes P1, P2, and P3.

For process P4, the steam dryer was not used because the waste heat from the combustion chamber and HCl absorption was utilized to provide the energy of the drying in an indirect dryer. Details on the flowsheet are provided in Figure 9, where two loops of water (CW1 and CW2) were applied to use the waste heat in the anhydrate dryer. Hot steam around 345 °C and cold steam around 110 °C are generated when the cooling water streams are used in the combustion chamber and the heat exchanger HE12, respectively. Both steam streams are mixed (HS) to obtain steam at 185 °C to provide the energy required in the dryer. The steam is condensed in the dryer to a temperature level of 155 °C. The temperature of the cooling water was then adjusted in a cooler (HE16) until 37 °C. The water flow rate and pressure in both loops were adjusted to achieve the desired temperatures.

**Figure 9:**
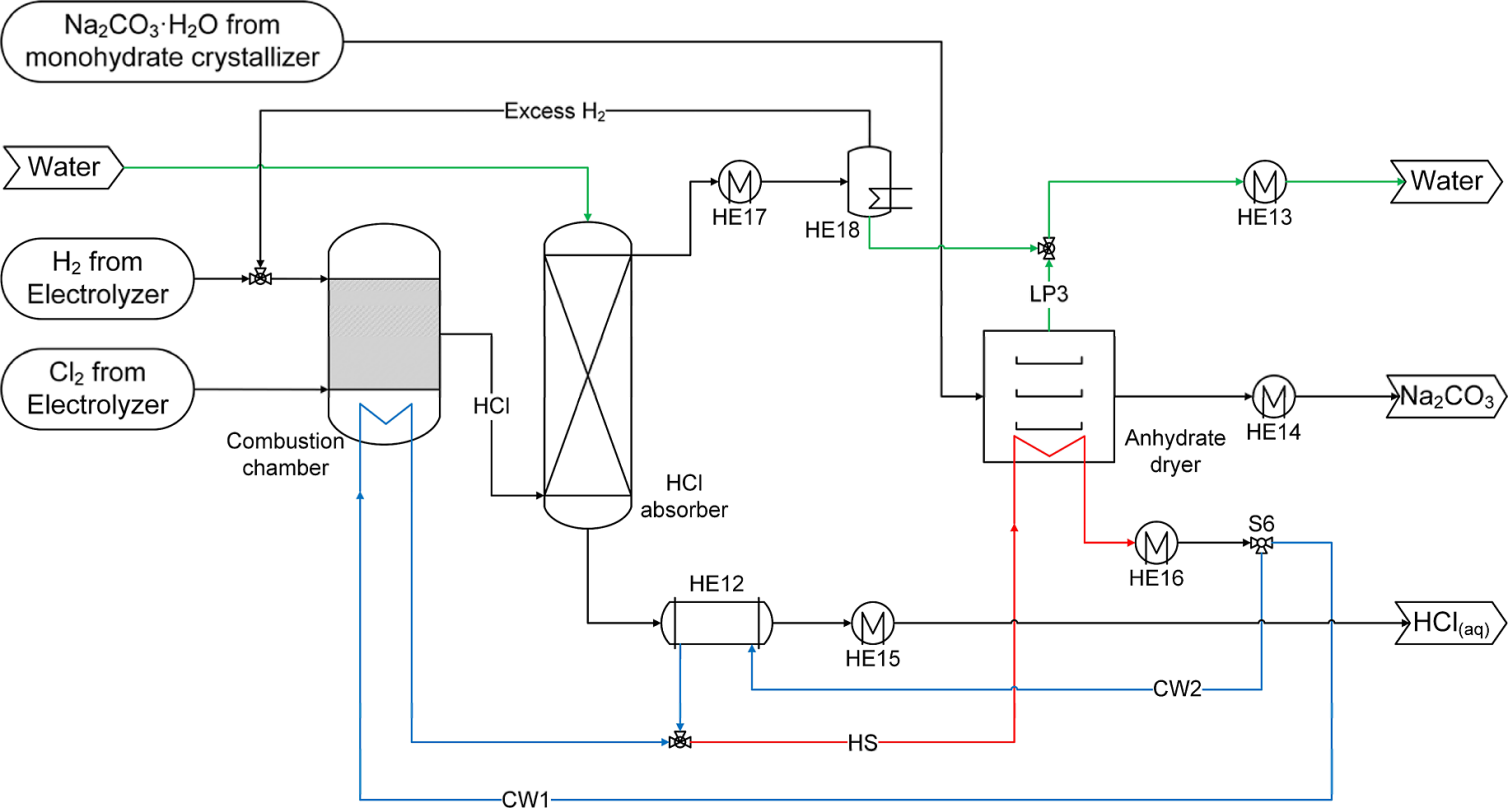
Flow diagram of the heat-integrated systems of HCl synthesis and the steam dryer for the dehydration of monohydrate crystals to produce soda ash (anhydrate) in process P4. Green line: high purity water, Blue line: cooling water, Red line: heating steam.

### 2.6 Complete Flowsheet Simulation

As presented in Figures 4, 5, and 7, the absorption and the crystallization are coupled in a process loop through recycling of the mother liquor. In order to achieve a stable convergence of the simulation, the flow and composition of the streams in the loop were estimated through global and component balances prior to the simulation. Details on the balances are presented in section D of the Supporting Information.

In the proposed processes, the water used in the humidification equipment is used as a cooling utility (see Figure 1). This was evaluated in the simulations in Aspen Plus V12 through the connection of two *Heater* blocks through an energy stream. The use of the cooled water was performed in series, and a pressure drop of 5 psi (∼345mbar) in each heat exchanger was assumed. The structure of the net of HE was selected to ensure a minimal temperature difference of 1 °C, assuming that a plate heat exchanger with counter-current flow regime is used. The proposed heat-integrated processes are the first schemes to evaluate the potential energy saving of the CODA process. Further improvements in the processes could be made by optimization.

Further heat integration of the process was performed by using the waste energy of the electrolysis system to avoid the use of low pressure and medium pressure steam from a boiler (or a power plant using a fossil fuel). In processes P1, P2, and P3, the energy required to heat and melt the decahydrate crystals from the absorption temperature to the monohydrate crystallization temperature (energy in the heat exchanger HE9 in Figure 6) was provided by the waste heat of the electrolysis system. This heat integration was performed, ensuring a minimal temperature difference of 5 °C in the heat exchanger (waste heat above 55 °C). In all processes, the remaining waste heat from electrolysis was utilized to reduce the heat consumption in the monohydrate crystallization heat exchanger (HE10). As a result, a part of the steam out of the crystallizer (LP2) was not used in the compressor (K2) and the electricity consumed was reduced.

The presented process alternatives are completely electrified, which means that no external heating energy is required. Only in process P1, some waste heating at low temperature (below 11 °C) is required to heat up the cooling water used in the humidification equipment. In process P2, an additional heat pump using butane as the working fluid was added to provide cooling energy. In processes P2 and P4, water loops were applied for heat integration to transfer the energy released in some location of the process to the equipment where energy was required.

### 2.7 Process Evaluation by Techno-Economic Assessment

The four CODA process variants were evaluated using the energy consumption (power, cooling, and heat) and the size of the main equipment. The power consumption of compressors and pumps was estimated in Aspen Plus V12. Only the energy required in the absorber pump (*E*_abs_) was estimated in Python, for which eq 9 was used. The absorber height (*H*), the liquid density (ρ_*L*_), the liquid mass flow rate ( $\dot{L_{in}}$ ), and the pump efficiency (η_*p*_) were required. For process P2, a nozzle pressure drop (Δ*P*_nozz_) was also required. Three additional major pumps were considered: the cooling water pump needed for the humidification and for cooling service in most heat exchangers and the recirculation pump for the absorption-crystallization loops (pumps P2 and P3 in Figures 4, 5, and 7). For pump P2, a pressure drop of 5 psi was considered (typical pressure drop in filters and hydrocyclones), while for pump P3, the discharge pressure was set in 1 bara so that the energy required for vacuum was somehow considered.

9 $$E_{abs}=\frac{\left(\rho_{L}gH+\Delta P_{nozz}\right)\dot{L_{in}}}{\eta_{p}\rho_{L}}$$

The energy required in the fans for the cross-flow packed absorber was estimated in Aspen Plus V12 using the specific pressure drop of the packing and the packing depth (Section 2.5) to calculate the total air pressure drop. In processes P1, P3, and P4, the depth of the packing required in the humidification (*Z*_h_) was added to the depth required in the absorption (8.6 m). The power required in the electrolysis process was estimated assuming the specific electricity consumption reported in the literature (2.62 MW-h/t Cl_2_,^19^). The cooling and heating requirement was estimated through the energy balances performed in Aspen Plus 12.

The number of electrolysis cells, total cells area, and total cells volume were estimated using specific values calculated from the literature (20.26 cells/MW, 2.72 m^2^/cell, 0.1 m^3^/cell^19^). The area of the heat exchangers was estimated with eq 1, the volume of the absorber with eq 5 or 7 depending on the type of absorber, and the volume of the crystallizers with eq 9.

A CO_2_ balance considering only the emission due to the electricity consumption was made from chloralkali electrolysis to the sodium anhydrate dryer. The CO_2_ emissions associated with the raw brine and the production of hydrogen and chlorine (or chlorohydric acid) were not considered in this work. Future work should focus on a proper life cycle analysis to evaluate the carbon negativity of the CODA process.

The net specific CO_2_ emission ( $e_{CO_{2}}$ in kg-eq CO_2_/kg soda) of the different process scenarios was also evaluated by using an emission factor ( $f_{CO_{2}}$ ) of 0.127 kg eq CO_2_/kW-h, which is the maximum value reported for onshore wind energy.^26^ Eq 10 presents the equation to calculate $e_{CO_{2}}$ , which shows that the total specific electricity consumption (*E*_total_ in kW-h/t soda), the flow of CO_2_ absorbed ( ${Ḟ}_{CO_{2}}=17.30ton/h$ ), and the flow of soda produced ( ${Ḟ}_{soda}=41.67ton/h$ ) were used. The last term in eq 10 represents the amount of CO_2_ absorbed per kilogram of soda ash produced (calculated from stoichiometry).

10 $$e_{CO_{2}}=f_{CO_{2}}E_{total}-\frac{\dot{F_{CO_{2}}}}{\dot{F_{soda}}}$$

The total specific electricity consumption was calculated by summing the electricity consumption of the main equipment in the CODA process (*E*_k_) and dividing the sum into the amount of soda ash produced, as presented in eq 11.

11 $$E_{total}=\frac{\sum_{k}E_{k}}{\dot{F_{soda}}}$$

Aiming to evaluate the influence of the type of renewable energy on the carbon-negativity of the CODA process, a maximal emission factor for carbon neutrality was calculated by making the net specific CO_2_ emission equal to zero ( $e_{CO_{2}}=0$ ) and solving eq 10 for the CO_2_ emission factor ( $f_{CO_{2}}$ ).

The cost of the main equipment was calculated with empirical correlations published in the literature, as presented in section E of Supporting Information. For the equipment in which the limit of the correlation was reached, the maximum limit of the size factor was used and the amount of equipment of the biggest factor was calculated. The total capital investment (TCI) was estimated with the Overall Factor Method with the formula and factor given in section E of the Supporting Information.

The capital investment cost per ton of product (CAPEX) was calculated using eq 12. The capital charge factor per year (*f*_c_) was assumed to be 10% (the TCI will be paid back within 10 years) and the operation time per year (*t*_op_) was fixed in 7225 h (continuous operation). Both assumptions were also done in economic analysis of DAC plants.^11^

12 $$CAPEX=\frac{f_{c}C_{TCI}}{t_{op}}$$

The operational cost per ton of product (OPEX) was calculated as the sum of the raw material cost and the electricity cost; other costs were not considered in the present cost analysis. The price of raw brine was assumed in 2 USD/ton, which is nearly 25% of the salt price,^27^ and the electricity price in 65 USD/MWh, which is the maximum price reported in 2024.^28^

The production cost was calculated as the sum of CAPEX and OPEX, and the profit was calculated as the difference between the sales revenue and the production cost. For the sales revenue, the 4 year average market prices (years 2020–2024) of the products were approximated from different sources as follows: soda ash 310 USD/ton,^29^ hydrogen 5000 USD/ton,^30^ chlorine 200 USD/ton,^31^ and hydrochloric acid 56.5 USD/ton.^32^ Since the CODA process absorbs CO_2_ from the air, the revenue of selling CO_2_ emission certificates was also considered in the economic evaluation by using a price of CO_2_ 75 USD/ton.^33^ A summary of the data used to evaluate CAPEX, OPEX, and sales revenue is presented in Table 6.

**Table 6:** Data Used to Evaluate CAPEX, OPEX, and Sales Revenue

CAPEX	OPEX	sales revenue
electrolysis cells	purified brine	soda ash price
CO_2_ absorber (including humidification for processes P1, P3, and P4)	electricity electrolysis	H_2_ price (not for process P4)
absorber pump	electricity absorber pumps	Cl_2_ price (not for process P4)
absorber fan	electricity absorber fans	HCl price 33 wt % (only for process P4)
compressors and blowers for MVR	electricity MVR compressors and blowers	CO_2_ emission certificates
compressor for heat pump (only for process P2)	electricity crystallization loops pumps	
hydrocyclone (not for process P3)		
pump decahydrate loop (not for process P3)		
decahydrate crystallizer (not for process P3)		
monohydrate crystallizer		

## 3 Results and Discussion

The results of the mass and energy balances for process variants P1, P2, P3, and P4 are presented in Figures 10, 11, 12, and 13, respectively. Screenshots of the simulation flowsheets are presented in section F of the Supporting Information. Heat duty of HE and power consumption of main pumps and compressors are displayed in the process flow diagrams. In addition, temperature, pressure, flow rate, and mass percentage of main streams are displayed. For the green, blue, and red lines, no composition is presented because these streams represent mainly water. HE with only one fluid and shaded in light blue correspond to those using cooling water from the humidification process as utility. Details on how the cooling utility is distributed (cooling heat exchanger net) are not given. Equipment shaded in strong blue require an additional cooling utility at a lower temperature level (below the temperature of the cooling water from the humidification). Equipment shaded in red require additional heating utility at a temperature above the ambient temperature (only required for process P1).

**Figure 10:**
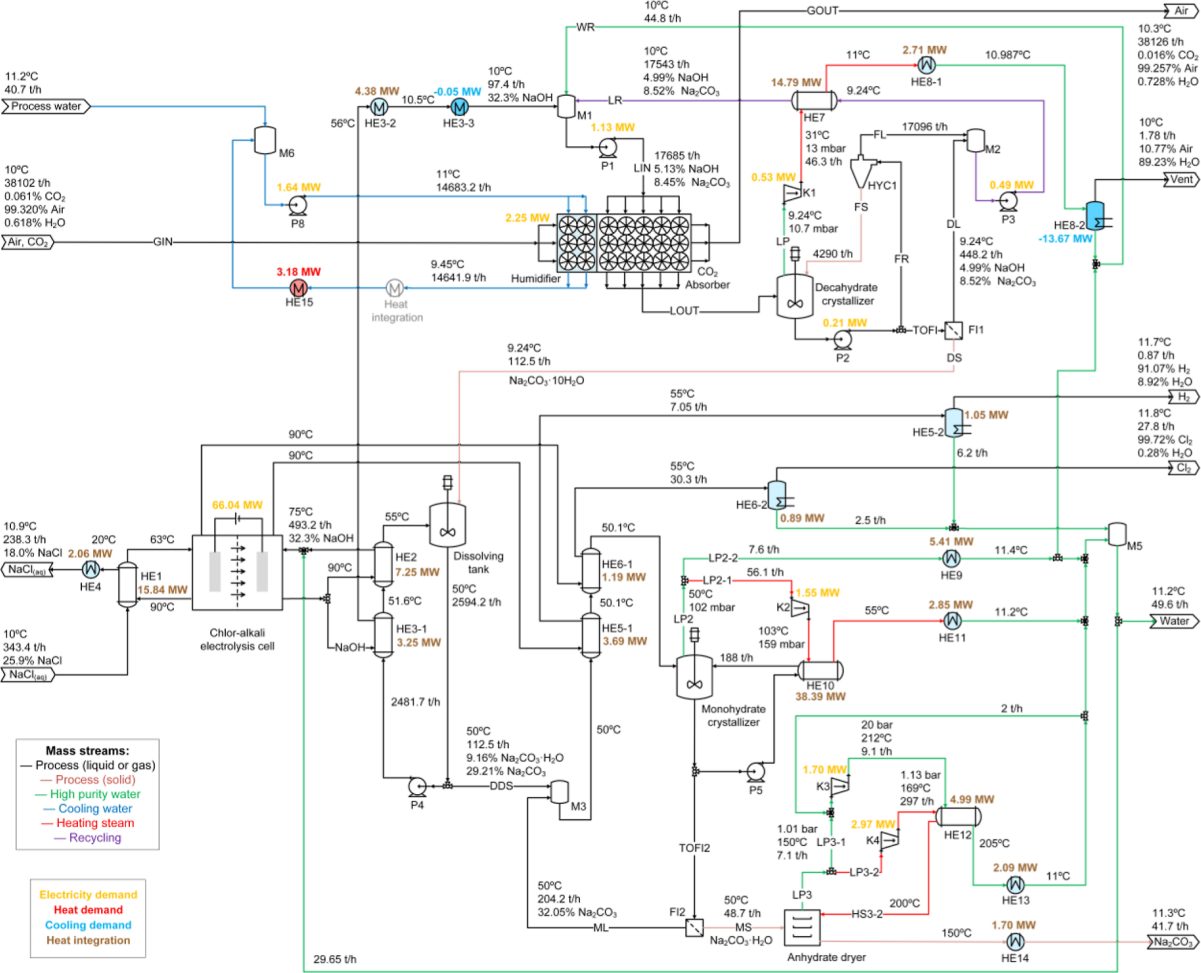
Overview of process P1 showing mass and energy balances including heat integration. Heat exchangers in light blue use cooling water from the humidifier as utility.

**Figure 11:**
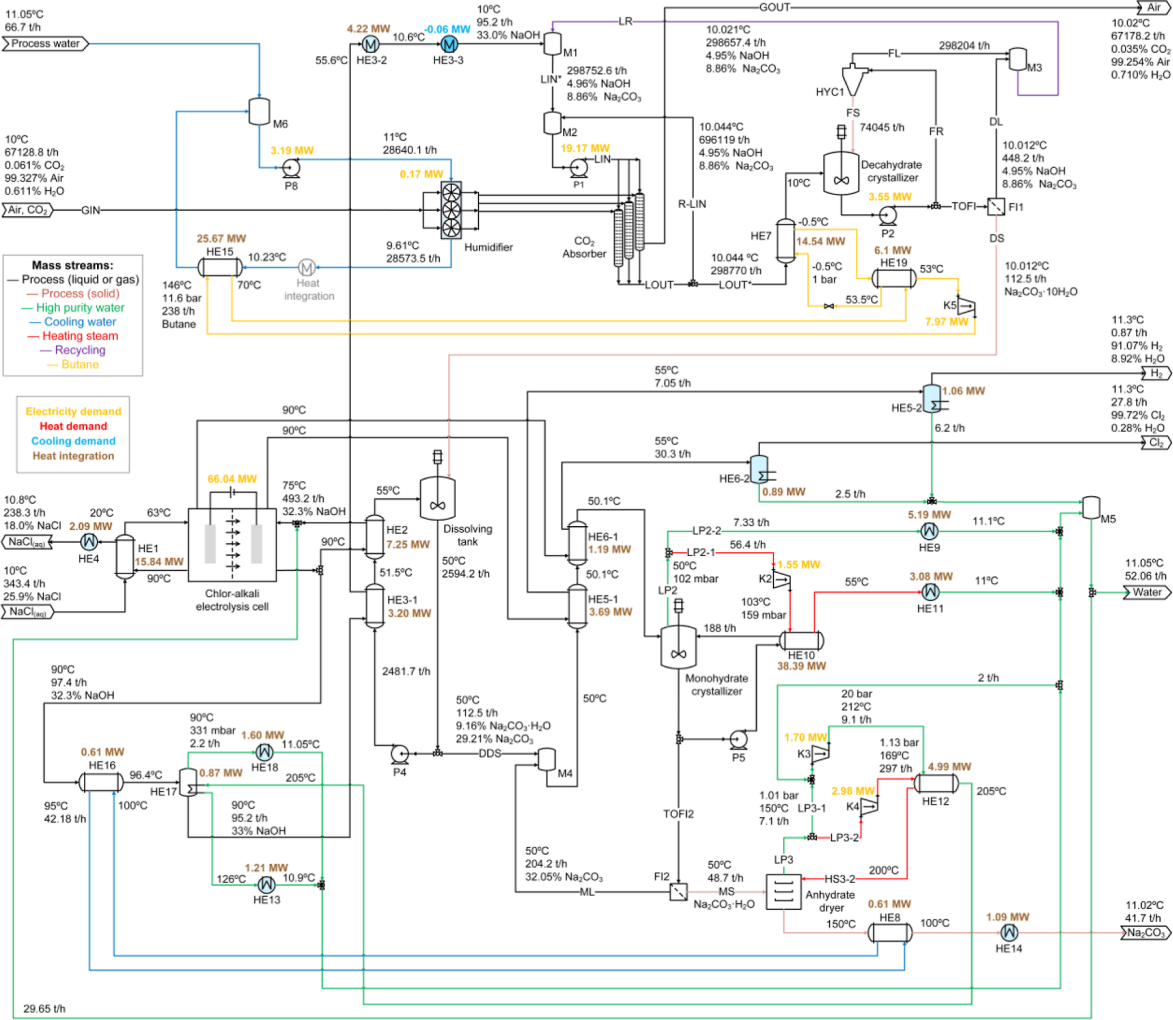
Overview of process P2 showing mass and energy balances including heat integration. Heat exchangers in light blue use cooling water from the humidifier as utility.

**Figure 12:**
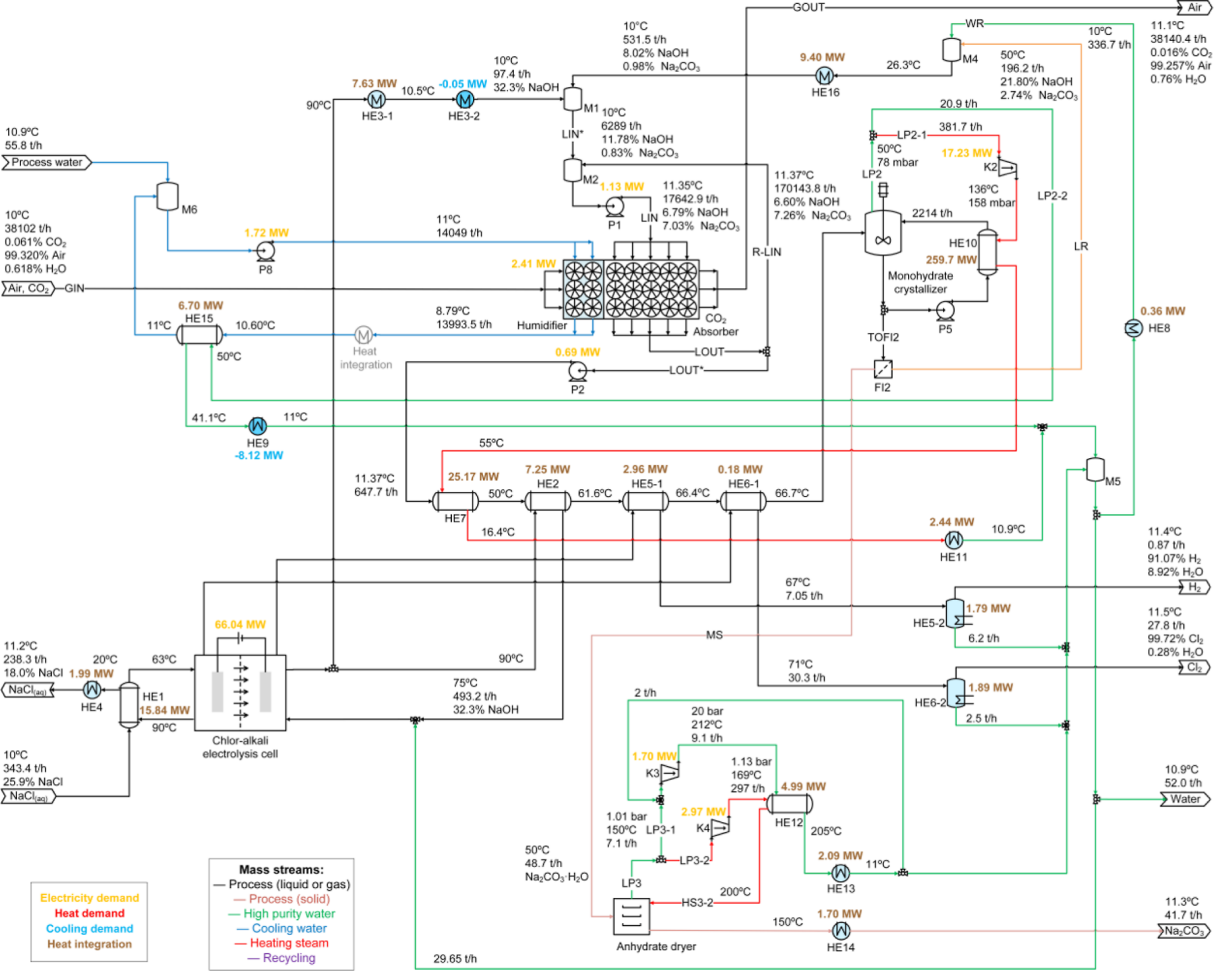
Overview of process P3 showing mass and energy balances including heat integration. Heat exchangers in light blue use cooling water from the humidifier as utility.

**Figure 13:**
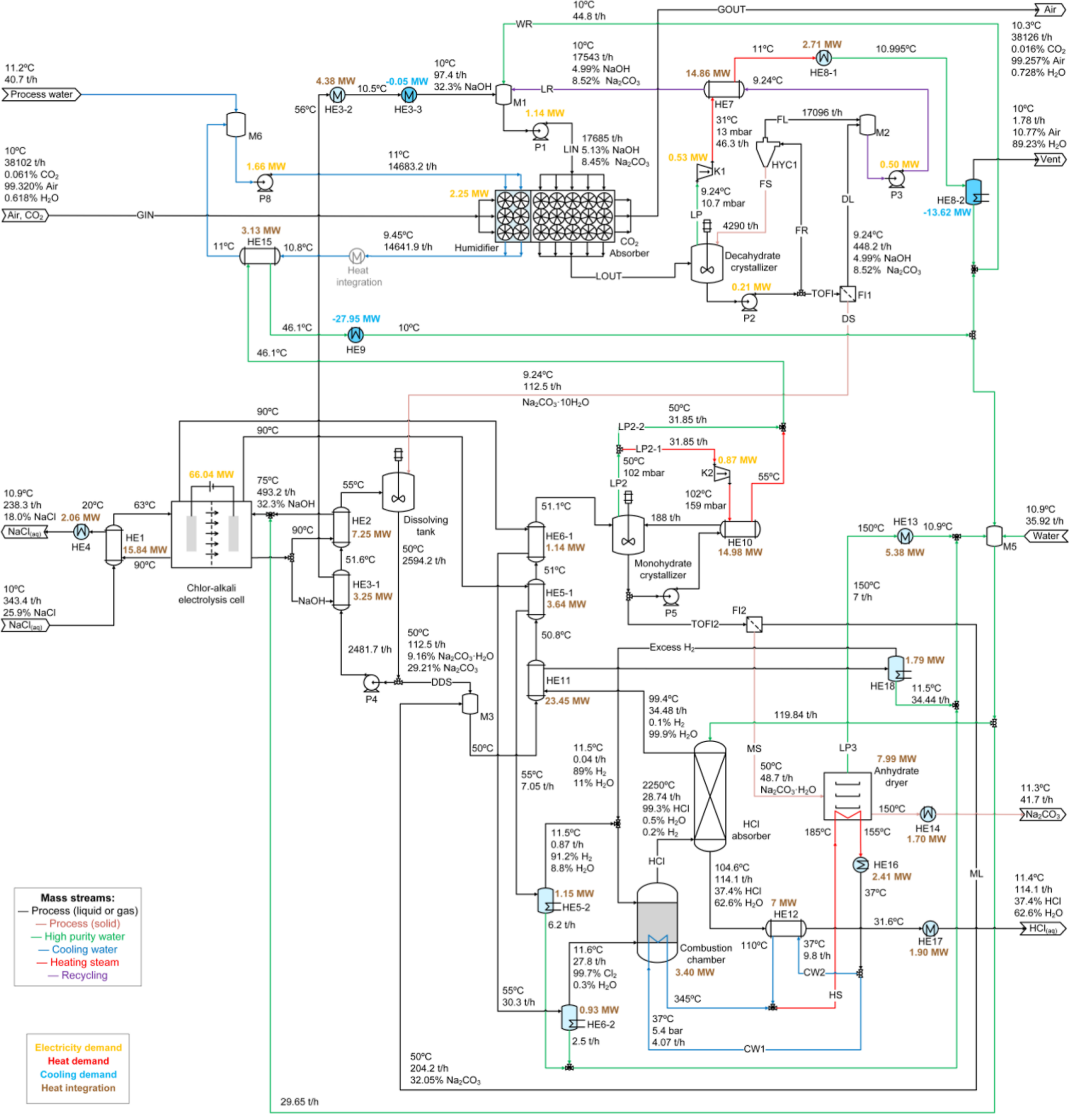
Overview of process P4 showing mass and energy balances including heat integration. Heat exchangers in light blue use cooling water from the humidifier as utility.

There are of course numerous alternative process design decisions for subvariants of this process study. For example, some water evaporation could be allowed in the absorber to save on the chiller and NaOH concentration or one could allow a slightly higher temperature in the decahydrate crystallizer to save on the chiller. Higher absorbers could also be permitted, or decahydrate, such as the monohydrate, could be crystallized by vacuum evaporation. However, these changes would entail further process adjustments, which would then usually require a process step elsewhere. In order to avoid getting lost in subvariants, certain design decisions were therefore retained in this study, which could of course also be optimized in further studies.

### 3.1 Process Variant P1-Packed Absorber and Decahydrate Crystallization

The CODA process scenario P1 was used to evaluate the use of a cross-flow packed absorber and the crystallization of sodium carbonate decahydrate. According to the results presented in Figure 10, chloralkali electrolysis consumes 85% of the total electricity required in the process. As a result, any effort to decrease the electricity consumption in the absorption and crystallization units will have a reduced impact on the total energy demand of the CODA process. On the other hand, the energy required in the absorber fans is 18% of the total electricity required in the compressors and pumps in the process. The ratio between the heat provided by the compressed steam and the work needed in the compressor (similar to a coefficient of performance) is very good in the decahydrate and monohydrate crystallization (28 and 25, respectively), which indicates an efficient use of the electric energy. This type of electrification requires steam compressors working at ambient temperature (10–50 °C) and vacuum pressure (10–160 mbar). Since the cost estimation done in this work was done for typical compressors (above ambient temperature), the investment cost of using this type of compressors in the process electrification should be still evaluated using vendor data.

The crystallization of sodium carbonate decahydrate directly after absorption reduces the stream flows in the downstream processing units. According to the simulation results, the mass flow of the stream entering the decahydrate crystallizer is 99% higher than the flow entering the monohydrate crystallizer. As a result, the size of the equipment related to monohydrate crystallization should be considerably smaller. In this study, the evaporation of water at low temperature and pressure was used to cool the mixture and to generate the required supersaturation. Even though the direct use of a cooling utility would be a more robust technology option for this process (no vacuum compressor required), the presented results allow evaluation of the benefits of using a potential new technology (10 mbara MVR).

The process flowsheet in Figure 10 allows us to observe that some waste heat from the electrolysis unit has been used to reduce the energy demand on the monohydrate crystallization unit (HE6–1 and HE6–2). Approximately 11% of the energy has been saved, which reduces the power in the compressor by 0.15 MW.

The electrification of the anhydrate dryer by using the steam dryer concept can be evaluated from the results in Figure 10. The high-pressure compressor (K3) handles a small steam flow in comparison to the low-pressure compressor (K4), which has a compression ratio smaller than 2 (it is practically a blower). They have a similar energy consumption (1.7–3 MW), and in total, the ratio heat/work of the steam dryer is below 2. Further study on the drying curve and the effect of the use of steam in the dryer is required for the implementation of the proposed steam dryer concept. The use of this type of dryer avoids the need of 7.99 MW of heat above 150 °C, which would require burning a fossil fuel (increase of CO_2_ emission) or additional investment for a solar thermal heat supply.

According to the obtained results, the inlet and outlet liquid stream of the absorber is very close to saturation (98–99%). This level of saturation can be reduced by increasing the crystallization temperature to operate under safer conditions, preventing crystal formation in the absorber due to unexpected changes in the temperature. However, the decrease in the crystallization will increase the cooling requirement in the crystallizer, which subsequently increases the flow of water to be evaporated and the power required in compressor K1. The results presented here are somehow on the edge of operational safety limits. From a sensitivity analysis performed in Aspen Plus V12, if the crystallization temperature is reduced from 9.23 to 8 °C, the saturation of inlet liquid stream would be 94%, the steam to be evaporated should be ∼80 t/h (instead of 46 t/h), the power to the compressor should be ∼1.33 MW (instead of 0.55 MW), and the heat transferred in HE7 would be ∼39 MW (instead of 14.79 MW).

### 3.2 Process Variant P2-Droplet Absorber and Decahydrate Crystallization

The process alternative P2 allows evaluation of the use of a different absorption technology on the CODA process. The first effect that can be recognized in the process structure is the elimination of the compressor K1 and the need to evaporate water in the decahydrate crystallizer to cool down the mixture and generate the supersaturation (see Figure 11). With the use of a droplet absorber, the supersaturation is achieved though the absorption, and there is no need to use cooling or evaporative crystallization. This operation requires certain safety measures to avoid blockage of the nozzle plates in the absorber. Even though there is no need to cool the stream out of the absorber to generate the crystallization, there is a slight temperature increase due to the CO_2_ absorption and reaction with NaOH, and some energy should be removed from the system in HE7 for the crystallizer to be adiabatic in the simulation. Though the heat to be removed is almost the same that should be removed in the cooling crystallization in process P1, the temperature difference is considerably smaller (0.03 °C instead of 1.29 °C in process P1). This shows that cooling provided in HE7 is not related to crystallization and that the high cooling demand in process P2 is due to the high mass flow rate of the stream to be cooled. In fact, due to the use of a droplet absorber, the flow rates of air and liquid are considerably different between processes P1 and P2. The volumetric flow rate of air in scenario P2 is 76% higher than that in process P1 due to the smaller capture efficiency of the selected geometry (42% instead of 74% in process P1, see Table 4). However, since the droplet absorber does not need a fan to move the air through the absorber, the fan energy consumption is not increased. The fan in process P2 is only used for the air humidification unit, which requires a considerably smaller packing depth than the absorber (0.38 m instead of 8.6 m); therefore, the energy consumption of the fan is small.

On the other hand, the small G/L ratio of the droplet absorber causes the volumetric liquid flow rate in process P2 to be almost 56 times bigger than the flow in process P1. This has a high impact on energy consumption and the size of the pump associated with the absorber (P1). The effect of the high liquid flow rate required in the droplet absorber was damped using an internal recirculation in the absorber (see Figure 11). Even though 70% of the liquid was recirculated to the absorber, the stream fed to the decahydrate crystallizer unit is 17 times higher than the stream produced in the packed absorber. Consequently, the filter pump (P2) in process P2 is considerably bigger than that in process P1. This disadvantage of the droplet absorber over the packed absorber has been discussed in a previous publication^15^ and reduces the economic feasibility of the process alternative P2 over the alternative P1.

Since process P2 does not use an EVC crystallization (because the droplet absorber generates the supersaturation), water is not evaporated in the absorber-crystallization loop. Consequently, it is required to adjust the concentration of the NaOH solution entering the loop to fulfill the water mass balance. The concentration of the NaOH solution should be 33 wt %, considering that 1 mol of water per mole of soda ash is generated in the reaction inside the absorber and 10 mol of water per mole of soda ash exits the loop in the decahydrate crystals (stream DS in Figure 11). The evaporation of water requires new equipment in process design (HE17). Some of the heat released by cooling the soda ash out of the anhydrate dryer (HE8) and the water removed in the dryer are used to provide the energy required in the evaporation of water. As a result, a loop using water as heating/cooling utility was added in process P2 to transfer the energy from the dryer (HE8) to a preheater (HE16). The pressure of the evaporator was adjusted in Aspen Plus V12 to have a temperature difference of 5 °C in HE16. Even though the energy required for this evaporation is not high compared with other energy requirements in the process (1.48 MW), the heat integration arrangement proposed in process P2 avoids the use of external heating utilities that would increase the carbon emission of the CODA process.

In contrast with the process alternative P1, the process variant P2 has a smaller external cooling demand and has no external heating demand. This is also caused by the type of absorber, which has an effect on the type of crystallization. The process P2 does not require to condense the water evaporated in the decahydrate crystallizer at low temperature and pressure in process P1 (HE3–2 in Figure 10). The external heating demand in process P2 is provided by a heat pump, which also provides the cooling energy required to remove the heat released in the absorber (HE7 in Figure 11). The heat pump is operated between −0.5 and 146 °C by using butane as the working fluid (yellow streams). The coefficient of performance of the heat pump is 3.6.

### 3.3 Process Variant P3-Packed Absorber and Monohydrate Crystallization

This single-stage crystallization strategy is evaluated through process scenario P3, in which sodium carbonate monohydrate is crystallized directly after absorption. The complexity of the process flow diagram and the number of equipment required is considerably reduced, as presented in Figure 12. The decahydrate crystallizer with a hydrocyclone and consequently also the dissolving tank for decahydrate are not needed in process P3. An internal recirculation loop in the absorber adjusts the feed flow to the absorber to obtain the required gas/liquid ratio in the absorber. The recirculation flow rate from the crystallizer to the absorber (LR) is 90 times smaller in process P3 than that in process P1 because there is no recycle from the decahydrate crystallizer added. Therefore, streams ML (P1 and P2) and LR (P3 only) are similar around 200 t/h of the mother liquor. Moreover, the technical feasibility of process alternative P3 is higher than that of process P1 since the size of the equipment handling solids is considerably smaller (filter separating 48.7 t/h of solids compared to a hydrocyclone separating 4290 t/h of solids).

On the other hand, the water to be removed from the monohydrate crystallizer is 6 times bigger when decahydrate is not previously crystallized. Consequently, the electricity and flow associated with compressor K2 are increased by 90% and the heat needed to be transferred in the heat exchanger HE10 is 6 times bigger for process P3 in comparison to process P1. This result was obtained after heat integration, in which some of the waste energy of the evaporated water is used to preheat the feed stream to the crystallizer (HE7 in Figure 12). In addition, the waste heat from the electrolysis, which was used in the dissolving tank in process P1, was now used to reduce the heat required in the monohydrate crystallization in scenario P3 (HE2 in Figure 12). The complete heat integration (including HE5–1 and HE6–1, also included in process P1) reduces the heat demand by 12%.

According to the results presented in the process flow sheet (Figure 12), the absorber in process P3 operates at a smaller saturation condition in comparison to the absorber in process P1. The saturation of the outlet stream of the absorber (LOUT) is around 92% (instead of 99% in process P1). The recirculation stream LR is not only smaller in process P3 but also contains less sodium carbonate and is highly concentrated in NaOH. The higher water evaporation in the monohydrate crystallizer increases the concentration of NaOH in process alternative P3 (21.89 wt % instead of 5.0 wt % in process P1). At such high NaOH concentration, the saturation concentration of Na_2_CO_3_ is smaller than that in the decahydrate crystallizer (2.69 wt % instead of 8.52 wt % in process P1). The influence of NaOH in the monohydrate crystallization is currently under study within the CODA project.^18^ The NaOH concentration in the monohydrate crystallizer could be adjusted by changing the NaOH concentration of the absorber operation, which was fixed around 5 wt % (6.79 wt % in Figure 12). As an example, if the NaOH concentration in the absorber is 2.4 wt %, the mother liquor out of the monohydrate crystallizer (LR) would have approximately 9.8 wt % NaOH (and 16.2 wt % Na_2_CO_3_). The temperature of operation of the absorber also influences the concentrations in the monohydrate crystallizer. For instance, if the temperature on the absorber raises up to 20 °C, the NaOH concentration in the crystallizer would be 17.2 wt % (and 6.8 wt % Na_2_CO_3_). The coupling of the absorber with the crystallization unit requires further analysis on the operation control focused on how the absorption temperature would influence the crystallization.

Due to the different NaOH and Na_2_CO_3_ concentrations in the liquid inlet of the absorber in process P3, the activity of water in the solution is also affected. This changes the water saturation concentration in air, and as a result, process P3 requires more process water in the humidification (55.8 t/h instead of 40.7 t/h in process P1). Moreover, the temperature of the absorber is also slightly different in processes P1 and P3, which also changes the absolute saturation humidity.

### 3.4 Process Variant 4-Hydrochloric Acid Production

The process alternative P4 was used to estimate how the CODA process would benefit from the techno-economic and emission points of view by the production of hydrochloric acid. The process flow diagram of process variant 4 in Figure 13 thus also includes the mass and energy balances of the combustion chamber and HCl absorber. In this process, the CO_2_ absorption and decahydrate crystallization units are basically the same as in process P1. In contrast, monohydrate crystallization uses some of the heat released in the HCl absorption to preheat the feed of the crystallizer (HE11 in Figure 13). This additional heat integration represents a 44% reduction of the electricity in the MVR compressor K3 and a reduction of the size of the heat exchanger HE10 associated with the monohydrate crystallizer (the heat demand in HE10 is reduced by 61%).

The anhydrate dryer of the process alternative P4 uses the energy released in the combustion chamber and the HCl absorber by means of a water/steam loop (Figure 13). By the use of the heat released in the HCl production, the electricity demand of the CODA process is reduced by 5.32 MW (0.68 MW in monohydrate crystallization and 4.68 MW in anhydrate dryer). This heat integration avoids the need of the compressors in the steam dryer arrangement (K3 and K4) but requires two additional complex pieces of equipment, which, however, are the industrial state of the art and therefore feasible (the combustion chamber and the HCl absorber). The additional equipment should be able to handle strong acid streams (corrosion resistant materials), which increases the investment cost. In addition, the combustion chamber operates at very high temperatures (2250 °C^7^), which also requires special equipment. The results obtained in the present study can be used in further analysis to decide if the production of HCl improves the economic feasibility of the CODA process, which mainly depends on the actual location of the system, in particular, whether there are consumers for the chlorine gas at the site.

### 3.5 Process Alternatives Comparison

The energy consumption and equipment size for the four CODA process scenarios are presented in Table 7. The electricity consumption was grouped in the main process units to allow further results analysis. In addition, the total heat provided by heat integration and the water usage is given for each process alternative. The total heat exchanger area is reported in Table 7, while the detailed information for each process and heat exchanger is provided in the Supporting Information, section F. In addition, the maximal liquid flow rate pumped in each process was reported to provide an idea of the size of the pumps in the process.

**Table 7:** Main Results of Energy Consumption and Equipment Size for the CODA Process Alternatives to Produce 1000 ton/day Soda Ash

parameter	units	P1 packed, deca-mono	P2 droplet, Deca-mono	P3 packed, mono	P4 packed, deca-mono, HCl
energy consumption					
electricity	MW	85.54	116.49	100.94	80.21
electrolysis	MW	73.05	73.05	73.05	73.05
absorber pumps	MW	1.13	19.18	1.13	1.14
absorber fans	MW	2.25	0.00	2.41	2.25
crystallization compressors	MW	6.76	17.53	21.91	1.40
other pumps	MW	2.34	6.64	2.44	2.37
external heating above 11 °C	MW	3.18	0	0	0
external cooling below 11 °C	MW	13.72	0.06	8.16	42.55
heat integration	MW	115.71	143.39	352.26	129.37
water usage					
process water demand	t/h	40.7	66.7	55.8	40.7
water out of the process	t/h	49.6	52.06	52.0	–35.92
equipment size					
electrolysis number of cells		1481	1481	1481	1481
electrolysis total area	m^2^	4027	4027	4027	4027
electrolysis volume	m^3^	148	148	148	148
volume absorber	m^3^	50583	17487	54178	50583
packing depth humidification	m	0.52	0.38	1.16	0.52
total heat transfer area	m^2^	19855	13704	29035	27681
max. pump flow rate	m^3^/h	15403	864646	15330	15330

The specific consumption of green electricity in the CODA process seems high (1.9–2.8 MW h/t), but it is the only energy source, and it does not exceed the total specific energy consumption of the Solvay process when all energy forms (natural gas and coal) are taken into account (∼2.9 MW h/t).^4^ However, the electricity consumption of the whole chemical industry is expected to increase due to the current transition into greener electrified processes.

The electrolysis unit requires between 63% for process P2 and 91% for process P4 of the total electricity demand. Since the electrolysis process is the same for all studied alternatives, this already shows that process P2 has the highest electricity demand and process P4 has the smallest one. By comparing the electricity consumption of processes P1 and P2, it can be seen that the use of the droplet absorber causes an increase in the electricity required in all units except the absorber fan. The increase in the work required by the absorber pump and by the crystallization compressors exceeds the reduction of the energy in the fan. The main reason for this is the high liquid flow rate in process P2 caused by a very small G/L ratio required in this technology and with a higher amount of air required due to a smaller capture efficiency. At this point, it must be pointed out that the droplet absorber could not be fully optimized in the CODA project. A cost optimization of the absorber design could probably reduce the energy expenditure by optimizing the three most important design specifications (nozzle diameter, nozzle density, and nozzle head diameter) for minimum cost.

The chlor-alkali electrolysis requires approximately 1500 cells of 0.1 m^3^ each and a total of 4027 m^2^ of area. The size of this unit is in the range of industrial electrolysis plants in current operation in Germany.^19^ The simulation approach followed in this study allowed us to evaluate the use of waste heat of the electrolysis unit in the down streaming process. In general, 10.5 MW of heat was provided by the electrolysis unit, which avoided the need of external heating (or electricity in a compressor) in the dissolving tank in processes P1, P2, and P4 and reduced the electricity demand of compressor K3 in process P3.

On the other hand, the use of the hydrogen and chlorine to produce hydrochloric acid HCl in process P4 reduces the electricity demand of the CODA process, so that this is the alternative with the smallest electricity requirement. However, due to the high exothermic nature of the HCl production, process scenario P4 has the biggest external cooling demand among the studied alternatives.

Although the direct crystallization of monohydrate after absorption (studied in process P3) significantly reduces the amount of equipment required (reduces investment costs), the electricity consumption of the entire process is increased. Interestingly, the increase in the electricity demand when decahydrate is not crystallized is almost as high as that when using the droplet absorber. The distribution of the electricity demand among the different equipment in the CODA process is graphically presented in Figure 14. The electrolysis unit is not included because its electricity demand is the same for all processes.

**Figure 14:**
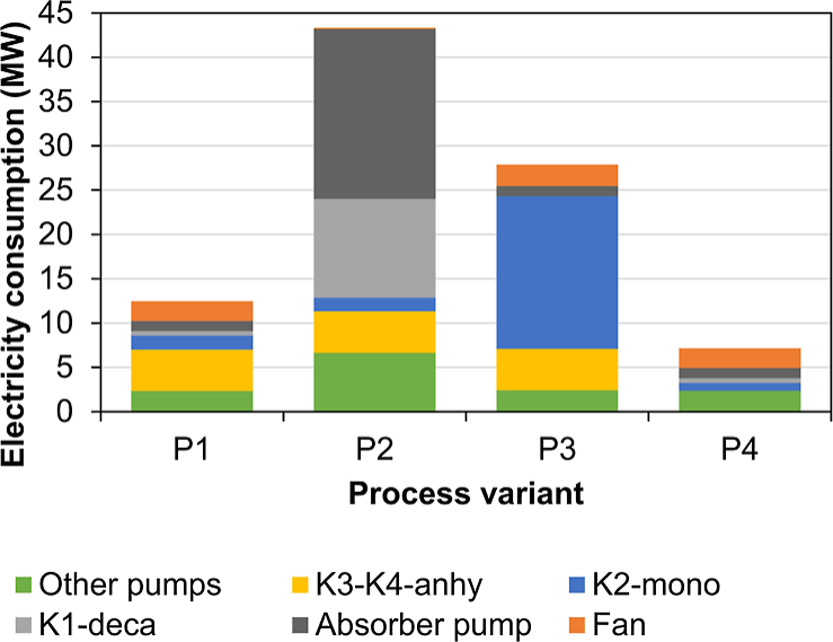
Comparison of electricity consumption in the studied CODA process alternatives to produce 1000 ton/day soda ash, excluding electrolysis electricity consumption (+73 MW for each variant).

As shown in Figure 14, the electricity consumed in the compressor associated with the monohydrate crystallization is considerably higher for process P3 than that for the other process alternatives. Moreover, it is very similar to the pump energy required in the droplet absorber. There is an important reduction in the electricity consumption in process alternative P4 due to the use of waste heat (generated in the HCl production) in the drying of monohydrate to obtain soda ash (yellow bar). The use of a droplet absorber as the CO_2_ absorber increases the energy consumption of process P2 in the absorber pump and the electricity consumption in the compressor associated with the decahydrate crystallization. Both effects are related to the high liquid flow required in this type of absorber (low G/L ratio).

The results of the purchase equipment and total investment cost estimation are summarized in Figure 15. The total investment costs are around six times bigger than the sum of the purchase equipment cost because it includes other type of costs such as land, engineering, allowances, and working capital. For the process alternatives using a cross-flow packed absorber (P1, P3, and P4), the cost of the absorber is more than 50% of the total equipment cost (when ignoring costs for the electrolysis plant). The purchase cost of the droplet absorber is considerably smaller than that of the cross-flow packed absorber because the droplet absorber is 2.9 times smaller than the cross-flow packed absorber (green bar). However, the higher cost of the absorber pump (orange bar) when using the droplet absorber results in a higher total equipment cost in the process alternative P2. As a result, the benefit of using a technology with a higher capture rate (droplet absorber) is diminished by the disadvantage of requiring higher liquid flow rates in the absorber. The comparison of these absorption technologies in terms of capital investment costs related to equipment size and operational costs related to energy consumption allows to select the cross-flow packed absorber as the preferred technology for the CODA process.

**Figure 15:**
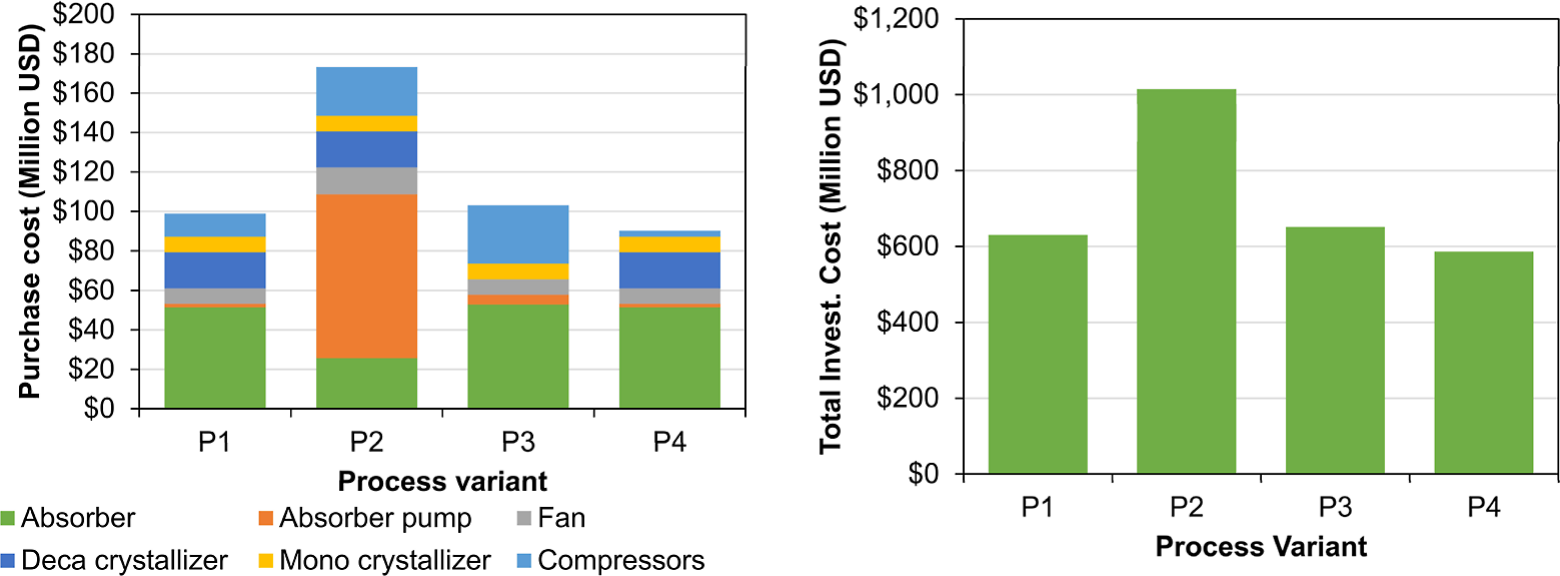
Comparison of purchase equipment costs (left) and total investment (right) costs in the studied CODA process alternatives to produce 1000 ton/day soda ash. Electrolysis costs of ∼121M$ are the same for all variants and thus not shown in the left figure but included in the total costs figure on the right.

It can be derived from Figure 15 that the cost of the compressor is highest for P3 and P2 in which most water must be evaporated under vacuum conditions. The expected reduction in investment cost of process variant P3, due to the direct crystallization of monohydrate (one crystallizer and filter less), is not observed, since the size and cost of the compressors are incremented in variant P3 due to the higher amount of water evaporated. As a result, from the operational and capital cost point of view, the two-step crystallization strategy (used in process variants P1, P2, and P4) is more favorable than the one-step-crystallization strategy (direct crystallization of monohydrate after absorption).

In contrast with the energy consumption, the equipment cost of process alternative P4 is not considerably smaller than that for the other alternatives. The use of waste heat from HCl production reduces the size of the compressors associated with the anhydrate drying; however, this has no big impact on the purchase equipment cost. Moreover, the additional costs related to the production of HCl were not considered here. As a result, the total cost of equipment for the process alternative P4 is underestimated.

The production costs and profits of all studied process alternatives are presented in Figure 16. The contributions of capital investment costs (CAPEX) and operational costs (OPEX) are also specified in the chart as bars. For all process alternatives, the CAPEX is significantly higher than the OPEX, which indicates a high dependence of the economic feasibility of the CODA process on the initial investment cost. The production costs of soda ash using the CODA process are between 336 and 535 USD/ton soda, which is more than the average market price for soda ash used in the calculations (310 USD/ton soda ash). As a result, the profit obtained in the CODA process is related with the revenue obtained by selling the byproducts. Selling Cl_2_ results in a revenue of 134 USD/ton of soda, while selling H_2_ results in a revenue of 95 USD/ton of soda. Revenues from selling CO_2_ certificates range from approximately 5 to 12 USD/ton soda depending on the net carbon negativity of each process variant, that is the net CO_2_ captured in the CODA process.

**Figure 16:**
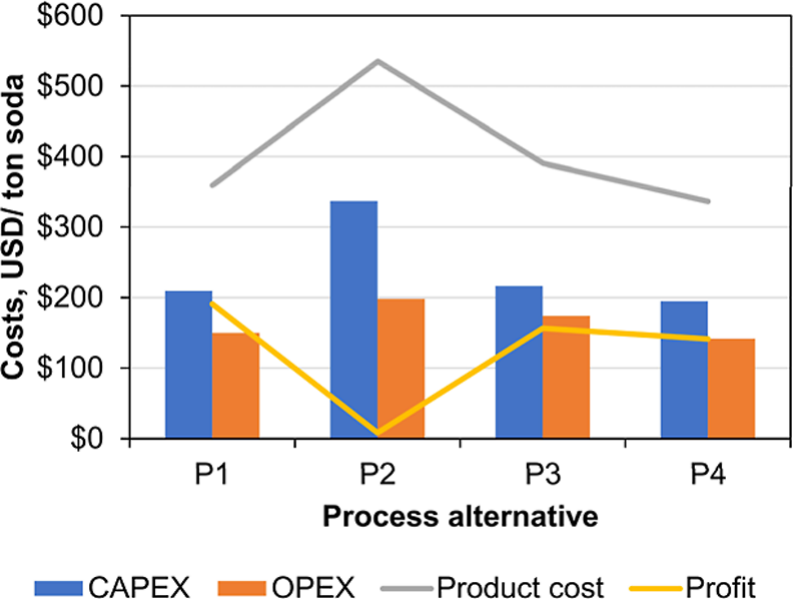
Preliminary cost analysis of the studied CODA process alternatives for a 1000 ton/day soda ash plant. CAPEX (blue), OPEX (orange), production costs (gray), profit including selling byproducts (yellow).

On the other hand, the comparison of the economical profit of the proposed process alternatives shows that the use of a cross-flow packed absorber for DAC and the crystallization of decahydrate in the downstream processes is the most economic viable CODA process arrangement (alternative P1 with 191 USD/ton soda ash profit). The use of waste heat from the HCl production results in a smaller production cost, but the profit is reduced because the byproduct is cheaper (the revenue from selling HCl is smaller than the revenue from selling H_2_ and Cl_2_).

The specific electricity consumption and CO_2_ emissions of the studied process alternatives can be used to compare them with other technologies. As presented in Table 8, the specific power consumption in the absorber obtained in the simulations is in the range of that reported in the literature. Smaller values are obtained in this study because we used optimized base case absorber design, while the value reported in Table 8 for the cross-flow packed absorber corresponds to the average between the pessimistic and optimiztic scenarios in the literature.^11^

**Table 8:** Main Specific Electricity Consumption and CO_2_ Emission the CODA Process Alternatives Compared to Other Technologies

parameter	units	P1 packed, deca-mono	P2 droplet, deca-mono	P3 packed, mono	P4 packed, deca-mono, HCl	other technologies
specific absorber power consumption	MW h/ton CO_2_	0.20	1.11	0.20	0.20	0.236 ± 0.110 (cross-flow packed)^11^
						0.237 ± 0.079 (counter-current packed)^10^
specific energy consumption	MW h/t soda	2.05	2.80	2.42	1.93	2.9 (Solvay process)^4^
net specific emission	kg-eq CO_2_/kg soda	–0.15	–0.06	–0.11	–0.17	0.79 (Solvay process)^34,35^
maximal emission factor for carbon neutrality	g-eq CO_2_/kW h	0.20	0.15	0.17	0.22	0.15 (solar thermal)
						0.30 (photovoltaic)
						0.08 (geothermal)
						0.07 (hydro)
						0.02 (nuclear)
						0.01 (wind offshore)
						0.13 (wind onshore)
						0.50 (natural gas)
						^26^

All of the CODA process alternatives presented in this study have a smaller specific energy consumption than the traditional Solvay process. From the analyzed scenarios, processes P1 and P4 are the most interesting because they reduce the specific energy consumption by approximately 1 MW h/t soda (33%). The proposed CODA process consumes not only less energy but also removes CO_2_ from the air. Considering the amount of CO_2_ absorbed in the CODA process and the equivalent amount of CO_2_ emitted using renewable electricity, the net specific emission of all process alternatives was calculated. Results in Table 8 reveal that the new process to produce soda ash is carbon-negative because the net emissions are negative. The net specific emission of the CODA process was compared with the emission of the Solvay process^34,35^ (positive emission). The evaluation of the net specific emission of the different process alternatives shows that the use of a droplet absorber (process P2) considerably affects the carbon negativity of the CODA process. Although the effect is not as strong, this is also the case of process P3, in which monohydrate is directly crystallized after absorption. The higher carbon negativity in processes P1 and P4 is achieved by expense of a bigger absorber and more equipment (capital investment cost).

Table 8 also contains the calculated maximal emission factor required for the CODA process to be carbon neutral for the studied process variants. For comparison purposes, the emission factor of different electricity sources was added. Using natural gas for the generation of the electricity required in the CODA process would result in a positive net emission of CO_2_ (not carbon negative). On the other hand, renewable energies with a smaller emission factor would result in a better carbon negativity of the CODA process, which would increase the economic feasibility of the process considering the revenue obtained by selling CO_2_ certificates.

The contribution to the CO_2_ emission by each section of the CODA process is presented in Figure 17. According to the results, more than 60% of the emissions of the CODA process are related to the electrolysis because it consumes over 60% of electricity of the process. The process design provided in the present study allows us to reduce the emission of the CODA process to its minimum, which is given by process alternative P4 (cross-flow packed absorber, crystallization of decahydrate after absorption, and use of waste heat from HCl production).

**Figure 17:**
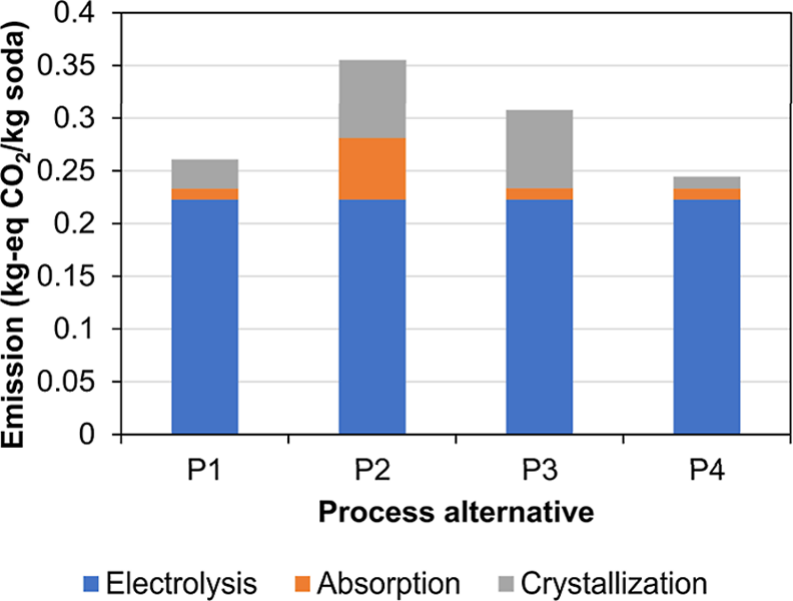
Specific emission of the CODA process alternatives using onshore wind energy as a source of electricity. Carbon-negative emissions are obtained considering the capture of 0.415 kg CO_2_/kg soda.

## 4 Conclusion

With the CODA process, a new and environmentally friendly process to produce soda ash was developed based on electrolysis of brine, CO_2_ DAC, and sodium carbonate crystallization. By means of process simulation and based on previous studies, complete process flowsheets with mass and energy balances of the production of soda ash by the CODA process were obtained. All the process variants were completely electrified by using mechanical vapor recompression, heat integration, and/or heat pump to provide the required heating demand.

Results showed that the selection of the absorption technology has a high impact on the technique and economic feasibility of the overall CODA process. Even though the droplet absorber technology has a better performance in terms of capture rate than the cross-flow packed absorber technology, the larger liquid flow rate required for its operation resulted in higher production costs related to pumping electricity consumption and size of equipment. As a result, the cross-flow packed absorber technology was selected as a preferred CO_2_ absorption technology in the CODA process. The crystallization of sodium carbonate decahydrate from the liquid out of the absorber followed by the crystallization of sodium carbonate monohydrate and its final dehydration was chosen as the separation strategy. The direct crystallization of monohydrate was related with higher electricity consumption and size of compressors due to the higher requirement of water evaporation.

The preliminary economic evaluation showed that the production costs of soda ash via the CODA process are higher than the market price, mainly due to a high capital investment cost. Even though the capital expenses have a big impact on the profitability of the CODA process, market prices of electricity, CO_2_ certificates, and soda ash also influence the profit. In fact, the economic feasibility of the CODA process involves revenue obtained by selling the byproducts of the process. Both chlorine and hydrogen contribute to the profitability of the CODA process. The production of aqueous HCl from the byproducts of the electrolysis reduced the electricity demand but decreased the profitability of the CODA process due to its lower selling price. The carbon-negativity of the process allows to sell CO_2_ certificates and increases the profitability of the CODA process. In contrast with traditional soda ash production, the carbon-negative production of soda ash could profit from its inherent sustainability.

The uncertainties of the economic evaluation of the CODA process are related to market prices for raw materials and electricity, equipment cost (especially the cost of the droplet absorber), and market values of the products. Even though the quantification of these uncertainties is out of focus in this study, typical numbers at this stage of the project, namely, feasibility study, are usually ±30%.

Regarding the CO_2_ emission, the uncertainties are also multifarious; specific emission of different electricity sources might be well-known, but the electricity consumption for utility processes could not be considered at this stage of the project and might add emissions and costs. Emission of the materials used for the construction of the CODA plant could also not be addressed at this stage of the study and would add to CO_2_ emissions. On the other hand, in the coming decades, the specific emission factors for electricity and materials will drop in course of the defossilization of the whole industry and thus the impact on the CODA emission might vanish in future.

Future work should focus on the technical evaluation of the compression of steam under vacuum, which was required for the electrification of the crystallization. In addition, the impact of the external cooling demand below 11 °C on the process feasibility should be evaluated. Finally, the study of the impact of ambient temperature and humidity on the electricity consumption would give insights into the location conditions in which the CODA process could benefit.

## Supplementary Material





## Notes

Simulation details (components, reactions, assigned property models), humidification submodel equations, validation of property model, initial mass balances, equations to estimate equipment cost and TCI, screenshots of the Aspen Plus flow sheets, and detailed simulation results (DOCX)
Simulation files in Aspen Plus V12 of the four CODA process variants (ZIP)

## List of Symbols and abbreviation

A: surface area for heat transfer, m^2^
a: packing specific surface area, m^2^/m^3^
*A*_e_: total cell area electrolysis, m^2^
*A*_in_: air inlet area to the absorber, m^2^
ANHY: sodium carbonate, Na_2_CO_3_
CAPEX: capital expenses per ton of product
*C*_b_: equipment base cost
CFD: computational fluid dynamic
CODA: carbon-negative sODA ash
*C*_p_: purchase cost
*C*_p,i_: purchase cost of equipment i
*C*_PL_: cost for the platforms and ladders
CS: condensate stream
*C*_TCI_: total capital investment cost
*C*_V_: cost for a vertical tower
CW: cooling water stream
*d*_50_: crystal diameter, μm
*D*: packing depth of the cross-flow packed absorber, m
*D*: absorber diameter used for the cost estimation, ft
DAC: direct air capture
DECA: sodium carbonate decahydrate, Na_2_CO_3_·10H_2_O
DL: mother liquor stream out of a filter after the decahydrate crystallization
DOUT: suspension stream out of decahydrate crystallizer
DDS: suspension stream out of the dissolving tank
DS: decahydrate crystals stream out of the filter
ρ_*L*_: density of the liquid feed into the absorber, kg/m^3^
Δ*P*_nozz_: nozzle pressure drop in the droplet absorber
*E*_abs_: energy required in the absorber pump, kW
$e_{CO_{2}}$: net specific CO_2_ emission, $\frac{kgeqCO_{2}}{kgsoda}$
*E*_k_: electricity consumption of the k equipment, kW
*E*_total_: total specific electricity consumption, $\frac{kW-h}{kgsoda}$
EV: evaporative vacuum
EVC: evaporative vacuum cooling
F: filter
${Ḟ}_{CO_{2}}$: CO_2_ absorption requirement, kgCO_2_/h
*f*_c_: capital charge factor, 10%
$f_{CO_{2}}$: CO_2_ emission factor, $\frac{kgeqCO_{2}}{kW-h}$
FL: mother liquor stream out of a hydrocyclone
*f*_LTCI_: Lang factor of Peters and Tommerhaus
*F*_M_: material factor
FS: decahydrate crystals stream out of a hydrocyclone
${Ḟ}_{soda}$: flow of soda produced, kg/h
*F*_T_: function type factor
*g*: gravity constant, m/s^2^
${Ġ}_{in}$: mass flow of inlet air in the absorber, kg/h
GIN: gas stream feed into the absorber
*G*_m_/*L*_m_: gas to liquid mass flow ratio in the absorber
GOUT: gas stream out of the absorber
*H*: absorber height, m
*H*: vessel height used for the cost estimation, ft
*H*: pump head, ft
*H* ^′^: enthalpy of the air–water mixture along the packing depth, J/kg
*H*_*i*_: vessel height used for the cost estimation, in
*H*_in_^′^: enthalpy of the air inlet to the humidifier, J/kg
*H*_out_^′^: enthalpy of the air outlet from the humidifier, J/kg
HE: heat exchanger
*H*_tOG_: theoretical height of transfer unit in the air humidifier, m
HP: high-temperature steam stream produced from the vapor compression
HS: high-temperature steam providing the heat duty to the anhydrate dryer
HYC: hydrocyclone
*I*_2023_: index cost factor for the year 2023 (808.8)
*I*_2009_: index cost factor for the year 2009 (521.9)
*I*_b_: base index cost (394)
*K*: compressor
*K*_G,h_: overall mass transfer coefficient in the air humidifier, m/s
${\mathrm{L̇}}_{in}$: mass flow of inlet liquid in the absorber, kg/h
LIN: liquid stream feed into the absorber
LIN*: liquid stream feed into the absorber loop
LOUT: liquid stream out of the absorber
LOUT*: liquid stream out of the absorber loop
LR: recycled liquid stream
LP: low-pressure steam stream evaporated in the crystallizer
M: mixing of streams
MF: suspension stream feed into the monohydrate crystallizer
ML: mother liquor stream out of the filter after the monohydrate crystallization
MS: monohydrate crystals streams out of the filter
MONO: sodium carbonate monohydrate, Na_2_CO_3_·H_2_O
MVR: mechanical vapor recompression
*N*: number of pumps of maximal size
*N*: number of fans of maximal size
*N*: number of crystallizers of maximal size
*n*_abs_: number of small absorbers in the droplet absorber
η_abs_: capture efficiency in the absorber
η_p_: pump efficiency
*N*_tOG_: number of transfer units in the air humidifier
OPEX: operational expenses per ton of product
P: pump
P1: process alternative 1: cross-flow absorber, decahydrate, and monohydrate crystallization
P2: process alternative 2: droplet absorber, decahydrate, and monohydrate crystallization
P3: process alternative 3: cross-flow absorber and monohydrate crystallization
P4: process alternative 4: cross-flow absorber, decahydrate, and monohydrate crystallization with HCl production
*P*_C_: power consumption in compressors or blowers, hp
*P*_C,i_: power consumption for each pump of maximal size, hp
PSD: particle size distribution
S: splitter
S: size of the pump, gpm*ft^0.5^
*S*_max_: maximal pump size, gpm*ft^0.5^
*t*_op_: operation time per year, h
${\mathrm{q̇}}_{i}$: liquid volumetric flow rate required in each nozzle plate of the droplet absorber, L/min
${\mathrm{r̂}}_{abs}$: flow of CO_2_ captured per gas inlet area, $\frac{kg}{m^{2}h}$
R-LIN: liquid stream recycled in the absorber loop
*Q*: heat duty (kW)
*Q*: liquid volumetric flow used in the pump cost or hydrocyclone estimation, gpm
*Q*: air volumetric flow used in the fan cost estimation, ft^3^/h
*Q*_max_: maximal volumetric flow in the fan, ft^3^/h
TCI: total capital investment
TOFI: suspension stream feed into filter
*t*_*s*_: thickness of the absorber sheet, in
Δ*T*_lm_: mean logarithmic temperature difference, °C
*U*: global heat transfer coefficient, $\frac{W}{m^{2{}^{\circ}}C}$
*V*_abs_: volume of the absorber (cross-flow or droplet), m^3^
*V*_*i*_: volume of the small droplet absorber, m^3^
v_G_: gas velocity, m/s
*W*: weight of the material required for the absorber in the cost estimation, lb
*W*: flow of crystals out of a crystallizer, ton/day
*W*_max_: maximal flow of crystals out of a crystallizer in the cost estimation, ton/day
WR: liquid stream recycled in the absorber-crystallizer loop (process P3)
*w*_*k*_: crystal mass fraction in the crystallizer suspension
$y_{CO_{2}}$: CO_2_ mass fraction at the inlet air in the absorber
*Z*_h_: packing depth for humidification, m
