# Supplementary material for: Carbon-Negative Production of Soda Ash: Process Development and Feasibility Evaluation
Source: Ind Eng Chem Res. 2025 May 30;64(23):11474–96. doi: 10.1021/acs.iecr.5c00483 (PMC12164680; doi:10.1021/acs.iecr.5c00483)
Supplement: Supplementary file 1 [file ie5c00483_si_001.docx]

**Supporting Information**

Carbon-Negative Production of Soda Ash: Process Development and Feasibility Evaluation

Maria F. Gutierrez*^a^, Heike Lorenz^a^, Peter Schulze^a^

^a^ Max Planck Institute for Dynamics of Complex Technical Systems, Sandtorstr. 1, 39106 Magdeburg, Germany.

# **Simulation details**

**Table S1.** List of components used in the simulation.

| **Component** | **Formula** | **Type** | **CAS number** | **Henry component** |
| --- | --- | --- | --- | --- |
| Water | $H_{2}O$ | Conventional | 7732-18-5 |  |
| Carbon dioxide | $CO_{2}$ | Conventional | 124-38-9 | X |
| Oxygen | $O_{2}$ | Conventional | 7782-44-7 | X |
| Nitrogen | $N_{2}$ | Conventional | 7727-37-9 | X |
| Sodium hydroxide | $NaOH$ | Conventional | 1310-73-2 |  |
| Hydrogen | $H_{2}$ | Conventional | 1333-74-0 | X |
| Chlorine | ${Cl}_{2}$ | Conventional | 7782-50-5 | X |
| Hydrogen chloride | $HCl$ | Conventional | 7647-01-0 | X |
| Sodium chloride | $NaCl$ | Conventional | 7647-14-5 |  |
| Hypochlorous acid | $HClO$ | Conventional | 7790-92-3 | X |
| Hidrogen ion | $H_{3}O^{+}$ | Conventional |  |  |
| Sodium ion | ${Na}^{+}$ | Conventional |  |  |
| Bicarbonate ion | ${HCO}_{3}^{-}$ | Conventional |  |  |
| Hydroxide ion | ${OH}^{-}$ | Conventional |  |  |
| Carbonate ion | ${CO}_{3}^{-}$ | Conventional |  |  |
| Hypochlorite ion | ${ClO}^{-}$ | Conventional |  |  |
| Chlorine ion | ${Cl}^{-}$ | Conventional |  |  |
| Sodium carbonate anhydrate | $Na_{2}CO_{3}$ | Solid | 497-19-8 |  |
| Sodium carbonate monohydrate | $Na_{2}CO_{3}\cdot H_{2}O$ | Solid |  |  |
| Sodium anhydrate heptahydrate | $Na_{2}CO_{3}\cdot7H_{2}O$ | Solid |  |  |
| Sodium carbonate decahydrate | $Na_{2}CO_{3}\cdot10H_{2}O$ | Solid |  |  |
| Sodium bicarbonate | $NaHCO_{3}$ | Solid | 144-55-8 |  |
| Sodium hydroxide solid | $NaOH$ | Solid | 1310-73-2 |  |
| Sodium chloride solid | $NaCl$ | Solid | 7647-14-5 |  |
| Sodium hydroxide monohydrate | $NaOH\cdot H_{2}O$ | Solid |  |  |
| Trona | $Na_{3}(HCO_{3})(CO_{3})\cdot2H_{2}O$ | Solid |  |  |
| Wegscheider | $Na_{5}\left( CO_{3} \right)\left( HCO_{3} \right)_{3}$ | Solid |  |  |

**Table S2.** *C*hemical reactions used in the simulation.

| **Reaction number** | **Reaction name** | **Reaction** | **Type** | **Equilibrium constants available** |
| --- | --- | --- | --- | --- |
| 1 | 1 | $HCl+H_{2}O\leftrightarrow Cl^{-}+H_{3}O^{+}$ | Equilibrium |  |
| 2 | 2 | $HClO+H_{2}O\leftrightarrow C{lO}^{-}+H_{3}O^{+}$ | Equilibrium | X |
| 3 | 3 | $Cl_{2}+2H_{2}O\leftrightarrow HClO+Cl^{-}+H_{3}O^{+}$ | Equilibrium | X |
| 4 | 4 | $HCO_{3}^{-}+H_{2}O\leftrightarrow CO_{2}^{-2}+H_{3}O^{+}$ | Equilibrium | X |
| 5 | 5 | $CO_{2}+2H_{2}O\leftrightarrow HCO_{3}^{-}+H_{3}O^{+}$ | Equilibrium | X |
| 6 | 6 | $2H_{2}O\leftrightarrow{OH}^{-}+H_{3}O^{+}$ | Equilibrium | X |
| 7 | MONO(S) | $Na_{2}CO_{3}\cdot H_{2}O\leftrightarrow2Na^{+}+CO_{3}^{2-}+H_{2}O$ | Salt | X |
| 8 | WEGSC(S) | $Na_{5}\left( CO_{3} \right)\left( HCO_{3} \right)_{3}\leftrightarrow5Na^{+}+3HCO_{3}^{-}+CO_{3}^{2-}$ | Salt |  |
| 9 | TRONA(S) | $Na_{3}\left( HCO_{3} \right)\left( CO_{3} \right)\cdot2H_{2}O\leftrightarrow3Na^{+}+HCO_{3}^{-}+CO_{3}^{2-}+2H_{2}O$ | Salt |  |
| 10 | NAOH:(S) | $NaOH\leftrightarrow Na^{+}+OH^{-}+H_{2}O$ | Salt |  |
| 11 | DECA(S) | $Na_{2}CO_{3}\cdot10H_{2}O\leftrightarrow2Na^{+}+CO_{3}^{2-}+10H_{2}O$ | Salt | X |
| 12 | HEPTA(S) | $Na_{2}CO_{3}\cdot7H_{2}O\leftrightarrow2Na^{+}+CO_{3}^{2-}+7H_{2}O$ | Salt | X |
| 13 | ANHY(S) | $Na_{2}CO_{3}\leftrightarrow2Na^{+}+CO_{3}^{2-}$ | Salt | X |
| 14 | NACL(S) | $NaCl\leftrightarrow Na^{+}+Cl^{-}$ | Salt | X |
| 15 | NAOH(S) | $NaOH\leftrightarrow Na^{+}+OH^{-}$ | Salt | X |
| 16 | BICA(S) | $NaHCO_{3}\leftrightarrow Na^{+}+HCO_{3}^{-}$ | Salt | X |
| 17 | NAOH | $NaOH\leftrightarrow Na^{+}+OH^{-}$ | Dissociation | - |
| 18 | NACL | $NaCl\leftrightarrow Na^{+}+Cl^{-}$ | Dissociation | - |

**Table S3.** Sets of chemistry reactions created for the simulation.

| **Reaction number** | **Reaction name** | **ALLGLOBA** | **GLOBAL** | **GLOBAL-2** | **SUPER** |
| --- | --- | --- | --- | --- | --- |
| 1 | 1 | X | X |  | X |
| 2 | 2 | X | X |  | X |
| 3 | 3 | X | X |  | X |
| 4 | 4 | X |  |  |  |
| 5 | 5 | X |  |  |  |
| 6 | 6 | X |  |  |  |
| 7 | MONO(S) | X | X | X | X |
| 8 | WEGSC(S) | X |  |  |  |
| 9 | TRONA(S) | X |  |  |  |
| 10 | NAOH:(S) | X |  |  |  |
| 11 | DECA(S) | X | X | X | X* |
| 12 | HEPTA(S) | X | X | X | X |
| 13 | ANHY(S) | X | X | X | X |
| 14 | NACL(S) | X |  |  |  |
| 15 | NAOH(S) | X | X |  | X |
| 16 | BICA(S) | X | X |  | X |
| 17 | NAOH | X | X |  | X |
| 18 | NACL | X |  |  |  |

* The equilibrium constant of this reaction was modified to avoid crystal formation in the absorber (RadFrac block)

**Table S4.** Assignment of Chemistry ID sets to each block in processes P1 and P2.

| **P1** | | | **P2** | | |
| --- | --- | --- | --- | --- | --- |
| **Block** | **Type** | **Chemistry ID** | **Block** | **Type** | **Chemistry ID** |
| ABS | Hierarchy | ALLGLOBA (Reactions:GLOBAL) | ABS | Hierarchy | SUPER (Reactions:GLOBAL) |
| ANOLYTE | Flash2 | ALLGLOBA | ANOLYTE | Flash2 | ALLGLOBA |
| CATHO | Flash2 | GLOBAL | CATHO | Flash2 | GLOBAL |
| COOLER | Heater | ALLGLOBA | COND | Heater | ALLGLOBA |
| COOLER2 | Heater | ALLGLOBA | COOLER | Heater | ALLGLOBA |
| COOLER3 | Heater | ALLGLOBA | COOLER3 | Heater | ALLGLOBA |
| COOLER4 | Heater | ALLGLOBA | COOLER4 | Heater | ALLGLOBA |
| COOLER5 | Heater | ALLGLOBA | COOLER5 | Heater | ALLGLOBA |
| COOLER21 | Heater | ALLGLOBA | COOLER6 | Heater | GLOBAL-2 |
| COOLP | Heater | ALLGLOBA | COOLER11 | Heater | ALLGLOBA |
| D-SPL | FSplit | ALLGLOBA | COOLP | Heater | ALLGLOBA |
| D-SPLIT | FSplit | None | COOLP1 | Heater | ALLGLOBA |
| D-TANK2 | Mixer | ALLGLOBA | D-SPL | FSplit | ALLGLOBA |
| DECA | Crystallizer | GLOBAL-2 | D-SPLIT | FSplit | None |
| DEHYDR | RStoic | GLOBAL-2 | DECA | Crystallizer | GLOBAL-2 |
| DISS | Crystallizer | GLOBAL-2 | DEHYDR | RStoic | GLOBAL-2 |
| DISTANK2 | Mixer | None | DIS-HI | Crystallizer | GLOBAL-2 |
| FAN | Compr | ALLGLOBA | DISTANK2 | Mixer | None |
| FI-1 | Filter | None | DISTANK3 | Mixer | None |
| FI-2 | Filter | None | ECON | HeatX | ALLGLOBA |
| FI-PU1 | Pump | ALLGLOBA | EVAP | Heater | ALLGLOBA |
| HE2 | Heater | ALLGLOBA | F-HX | Heater | None |
| HE3-1 | Heater | ALLGLOBA | F-HX2 | Heater | None |
| HE3-2 | Heater | ALLGLOBA | FAN | Compr | ALLGLOBA |
| HE3C | Heater | None | FI-1 | Filter | None |
| HE4 | Heater | ALLGLOBA | FI-2 | Filter | None |
| HE5-1 | Heater | None | FI-PU1 | Pump | ALLGLOBA |
| HE5-2 | Flash2 | None | HE2 | Heater | ALLGLOBA |
| HE6-1 | Heater | None | HE3-1 | Heater | ALLGLOBA |
| HE6-2 | Flash2 | None | HE3-2 | Heater | ALLGLOBA |
| HI-EL | Heater | ALLGLOBA | HE3C | Heater | None |
| HI-EL-C | Heater | ALLGLOBA | HE4 | Heater | ALLGLOBA |
| HI-HE-C | Heater | ALLGLOBA | HE5-1 | Heater | None |
| HI-HE-H | Heater | ALLGLOBA | HE5-2 | Flash2 | None |
| HI-REC | HeatX | ALLGLOBA | HE6-1 | Heater | None |
| HICO | Heater | ALLGLOBA | HE6-2 | Flash2 | None |
| HICO4 | Heater | ALLGLOBA | HI-COP1 | Heater | ALLGLOBA |
| HICO5 | Heater | ALLGLOBA | HI-EL | Heater | ALLGLOBA |
| HICO21 | Heater | ALLGLOBA | HI-EL-C | Heater | ALLGLOBA |
| HICOPR | Heater | ALLGLOBA | HI-HX11 | Heater | ALLGLOBA |
| HIHE2 | Heater | None | HI-REC | HeatX | ALLGLOBA |
| HIHE4 | Heater | ALLGLOBA | HICO | Heater | ALLGLOBA |
| HIHE31 | Heater | None | HICO4 | Heater | ALLGLOBA |
| HIHE32 | Heater | ALLGLOBA | HICO5 | Heater | ALLGLOBA |
| HIHE51 | Heater | None | HICO6 | Heater | ALLGLOBA |
| HIHE52 | Heater | ALLGLOBA | HICOPR | Heater | ALLGLOBA |
| HIHE61 | Heater | None | HIHE2 | Heater | None |
| HIHE62 | Heater | ALLGLOBA | HIHE4 | Heater | ALLGLOBA |
| HUMID | Hierarchy | None | HIHE31 | Heater | None |
| HX-1 | Flash2 | ALLGLOBA | HIHE32 | Heater | ALLGLOBA |
| HX-AB | Heater | ALLGLOBA | HIHE51 | Heater | None |
| HYC-1 | HyCyc | None | HIHE52 | Heater | ALLGLOBA |
| K-1 | Compr | ALLGLOBA | HIHE61 | Heater | None |
| K-2 | Compr | ALLGLOBA | HIHE62 | Heater | ALLGLOBA |
| K-3 | Compr | ALLGLOBA | HUMT | RadFrac | None |
| K-4 | Compr | ALLGLOBA | HX-1 | Flash2 | ALLGLOBA |
| M3 | Mixer | None | HX-11 | Heater | None |
| MIXDIS | Mixer | None | HX-AB | Heater | ALLGLOBA |
| MONO | Crystallizer | GLOBAL-2 | HYC-1 | HyCyc | None |
| MONO-JCK | Heater | ALLGLOBA | K-1 | Compr | ALLGLOBA |
| PU-R | Pump | ALLGLOBA | K-3 | Compr | ALLGLOBA |
| PU-R3 | Pump | ALLGLOBA | K-4 | Compr | ALLGLOBA |
| PU-R4 | Pump | ALLGLOBA | K-5 | Compr | ALLGLOBA |
| PU-WFC | Pump | ALLGLOBA | M3 | Mixer | None |
| PW-TNK | Mixer | ALLGLOBA | MIX-DIS | Mixer | None |
| REC-TNK | Mixer | ALLGLOBA | MONO | Crystallizer | GLOBAL-2 |
| SPL-ST | FSplit | ALLGLOBA | MONO-JCK | Heater | ALLGLOBA |
| SPL-W1 | FSplit | ALLGLOBA | PU-WFC | Pump | ALLGLOBA |
| SPL-W2 | FSplit | ALLGLOBA | PW-TNK | Mixer | ALLGLOBA |
| SPL-W3 | FSplit | ALLGLOBA | R-SPLIT | FSplit | None |
| SPL1 | FSplit | ALLGLOBA | REC-TNK | Mixer | ALLGLOBA |
| SPLDISS | FSplit | None | SPL-DIS | FSplit | None |
| V-1 | Flash2 | ALLGLOBA | SPL-ST | FSplit | ALLGLOBA |
| V-2 | Valve | ALLGLOBA | SPL-W2 | FSplit | ALLGLOBA |
| V-3 | Valve | ALLGLOBA | SPL1 | FSplit | ALLGLOBA |
| W-PU | Pump | ALLGLOBA | TV | Valve | ALLGLOBA |
| W-TANK | Mixer | ALLGLOBA | V-2 | Valve | ALLGLOBA |
|  |  |  | V-3 | Valve | ALLGLOBA |
|  |  |  | W-PU | Pump | ALLGLOBA |
|  |  |  | W-TANK | Mixer | ALLGLOBA |

**Table S5.** Assignment of Chemistry ID sets to each block in processes P1 and P2.

| **P3** | | | **P4** | | |
| --- | --- | --- | --- | --- | --- |
| **Block** | **Type** | **Chemistry ID** | **Block** | **Type** | **Chemistry ID** |
| ABS | RadFrac | ALLGLOBA (Reactions:GLOBAL) | ABS | Hierarchy | ALLGLOBA (Reactions:GLOBAL) |
| ANOLYTE | Flash2 | ALLGLOBA | ABS-HCL | RadFrac | ALLGLOBA (Reactions:ALLGLOBA) |
| CATHO | Flash2 | GLOBAL | ANOLYTE | Flash2 | ALLGLOBA |
| COOLER | Heater | ALLGLOBA | CATHO | Flash2 | GLOBAL |
| COOLER2 | Heater | ALLGLOBA | COND | Flash2 | ALLGLOBA |
| COOLER3 | Heater | ALLGLOBA | COOLER | Heater | ALLGLOBA |
| COOLER4 | Heater | ALLGLOBA | COOLER2 | Heater | ALLGLOBA |
| COOLER5 | Heater | ALLGLOBA | COOLER4 | Heater | ALLGLOBA |
| COOLER6 | Heater | None | COOLER7 | Heater | ALLGLOBA |
| COOLER7 | Heater | None | COOLER8 | Heater | ALLGLOBA |
| COOLER71 | Heater | None | COOLER9 | Heater | ALLGLOBA |
| COOLP | Heater | ALLGLOBA | COOLER21 | Heater | ALLGLOBA |
| D-SPL | FSplit | ALLGLOBA | COOLER41 | Heater | ALLGLOBA |
| DEHYDR | RStoic | GLOBAL-2 | COOLER71 | Heater | ALLGLOBA |
| DISTANK2 | Mixer | None | COOLP | Heater | ALLGLOBA |
| FAN | Compr | ALLGLOBA | D-SPLIT | FSplit | None |
| FI-2 | Filter | None | D-TANK2 | Mixer | ALLGLOBA |
| HE2 | Heater | ALLGLOBA | DECA | Crystallizer | GLOBAL-2 |
| HE3 | Heater | ALLGLOBA | DEHYDR | RStoic | GLOBAL-2 |
| HE3C | Heater | None | DISS | Crystallizer | GLOBAL-2 |
| HE4 | Heater | ALLGLOBA | DISTANK2 | Mixer | None |
| HE5-1 | Heater | None | FAN | Compr | ALLGLOBA |
| HE5-2 | Flash2 | None | FI-1 | Filter | None |
| HE6-1 | Heater | None | FI-2 | Filter | None |
| HE6-2 | Flash2 | None | FI-PU1 | Pump | ALLGLOBA |
| HI-EL | Heater | ALLGLOBA | HE2 | Heater | ALLGLOBA |
| HI-EL-C | Heater | ALLGLOBA | HE3-1 | Heater | ALLGLOBA |
| HI-PR | Heater | None | HE3-2 | Heater | ALLGLOBA |
| HI-REC | HeatX | ALLGLOBA | HE3C | Heater | None |
| HICO | Heater | ALLGLOBA | HE4 | Heater | ALLGLOBA |
| HICO2 | Heater | ALLGLOBA | HE5-1 | Heater | None |
| HICO4 | Heater | ALLGLOBA | HE5-2 | Flash2 | None |
| HICO5 | Heater | ALLGLOBA | HE6-1 | Heater | None |
| HICO6 | Heater | ALLGLOBA | HE6-2 | Flash2 | None |
| HICOP | Heater | ALLGLOBA | HI-CO71 | Heater | ALLGLOBA |
| HIHE2 | Heater | None | HI-COND | Heater | ALLGLOBA |
| HIHE3 | Heater | ALLGLOBA | HI-DEHY | Heater | ALLGLOBA |
| HIHE4 | Heater | ALLGLOBA | HI-EL | Heater | ALLGLOBA |
| HIHE51 | Heater | None | HI-EL-C | Heater | ALLGLOBA |
| HIHE52 | Heater | ALLGLOBA | HI-HE-C | Heater | ALLGLOBA |
| HIHE61 | Heater | None | HI-HE-H | Heater | ALLGLOBA |
| HIHE62 | Heater | ALLGLOBA | HI-STHCL | Heater | ALLGLOBA |
| HUM | RadFrac | None | HICO | Heater | ALLGLOBA |
| HX-1 | Flash2 | ALLGLOBA | HICO7 | Heater | ALLGLOBA |
| HX-AB | Heater | ALLGLOBA | HICO8 | Heater | ALLGLOBA |
| K-1 | Compr | ALLGLOBA | HICO9 | Heater | None |
| K-3 | Compr | ALLGLOBA | HICO21 | Heater | ALLGLOBA |
| K-4 | Compr | ALLGLOBA | HICOPR | Heater | ALLGLOBA |
| M-TANK2 | Mixer | ALLGLOBA | HIHE2 | Heater | None |
| MONO | Crystallizer | GLOBAL-2 | HIHE4 | Heater | ALLGLOBA |
| MONO-JCK | Heater | ALLGLOBA | HIHE31 | Heater | None |
| PR | Heater | None | HIHE32 | Heater | ALLGLOBA |
| PU-R | Pump | ALLGLOBA | HIHE51 | Heater | None |
| PU-R3 | Pump | ALLGLOBA | HIHE52 | Heater | ALLGLOBA |
| PU-WFC | Pump | ALLGLOBA | HIHE61 | Heater | None |
| PW-TNK | Mixer | ALLGLOBA | HUMID | Hierarchy | None |
| SPL-ST | FSplit | ALLGLOBA | HX-1 | Flash2 | ALLGLOBA |
| SPL-W2 | FSplit | ALLGLOBA | HX-AB | Heater | ALLGLOBA |
| SPL1 | FSplit | ALLGLOBA | HYC-1 | HyCyc | None |
| SPLIT | FSplit | ALLGLOBA | K-1 | Compr | ALLGLOBA |
| SPLWO | FSplit | None | K-2 | Compr | ALLGLOBA |
| V-1 | Valve | ALLGLOBA | M1 | Mixer | None |
| V-2 | Valve | ALLGLOBA | M3 | Mixer | None |
| V-3 | Valve | ALLGLOBA | MIX-ST | Mixer | ALLGLOBA |
| W-PU | Pump | ALLGLOBA | MIX-WL | Mixer | ALLGLOBA |
| W-TANK | Mixer | ALLGLOBA | MIXDIS | Mixer | None |
|  |  |  | MONO | Crystallizer | GLOBAL-2 |
|  |  |  | MONO-JCK | Heater | ALLGLOBA |
|  |  |  | PU-R | Pump | ALLGLOBA |
|  |  |  | PU-R3 | Pump | ALLGLOBA |
|  |  |  | PU-WFC | Pump | ALLGLOBA |
|  |  |  | PW-TNK | Mixer | ALLGLOBA |
|  |  |  | REC-TNK | Mixer | ALLGLOBA |
|  |  |  | SPL-ST | FSplit | ALLGLOBA |
|  |  |  | SPL-W1 | FSplit | ALLGLOBA |
|  |  |  | SPL-W3 | FSplit | ALLGLOBA |
|  |  |  | SPL-WL | FSplit | ALLGLOBA |
|  |  |  | SPL1 | FSplit | ALLGLOBA |
|  |  |  | SPLDISS | FSplit | None |
|  |  |  | SPLT-H2 | FSplit | ALLGLOBA |
|  |  |  | ST-HCL | RStoic | ALLGLOBA |
|  |  |  | V-1 | Flash2 | ALLGLOBA |
|  |  |  | V-2 | Valve | ALLGLOBA |
|  |  |  | W-PU | Pump | ALLGLOBA |
|  |  |  | W-TANK | Mixer | ALLGLOBA |

# **Humidification sub-model**

**Table S6.** Equations used to calculate the packing depth in the humidification.

| **Equation** | **Units** | **References** |
| --- | --- | --- |
| Overall mass transfer coefficient of water in the gas phase in the humidification:  $K_{G,h}\approx k_{G,h}$ | $K_{G,h}[m/s]$  $k_{G,h}\left[ m/s \right]$ | - |
| Local mass transfer coefficient of water in the gas phase in the humidification (Bravo, Rocha, Fair, 1985 model):  $k_{G,h}=\left( 0.0338{Re}_{G}^{0.8}Sc_{G}^{1/3} \right)\frac{D_{w,G}}{d_{eq}}$ | $k_{G,h}\left[ \frac{m}{s} \right]$  $Re_{G}\left[ - \right]$  $Sc_{G}[-]$  $D_{w,G}\left[ m^{2}/s \right]$  $d_{eq}[m]$ | (Flagiello, 2021) |
| Diffusivity of water in air:  $D_{w,G}=\frac{{10}^{-4}\left( 1.084-0.249\sqrt{\frac{1}{18.0153}+\frac{1}{44.0098}} \right)T^{\frac{3}{2}}\sqrt{\frac{1}{18.0153}+\frac{1}{28.97}}}{P\left( \frac{0.3711+0.2641}{2} \right)^{2}f}$ | $D_{w,G}\left[ m^{2}/s \right]$  $T\left[ K \right]$  $P\left[ Pa \right]$ | (Treybal 1980) |
| Reynolds number of the gas in a cross-flow packed*:  $Re_{G}=\frac{d_{eq}\rho_{G}v_{G}}{\mu_{G}}$  * usually the relative velocity is used ($v_{G}+v_{L})$, but here only $v_{G}$ is used because of the cross-flow arrangement. | $d_{eq}[m]$  $\rho_{G}\left[ kg/m^{3} \right]$  $v_{G}\left[ m/s \right]$  $v_{L}[m/s ]$ | (Flagiello et al. 2021) |
| Characteristic packing dimension (equivalent diameter) (Bravo, Rocha, Fair, 1985 model):  $d_{eq}=\frac{B_{p}H_{p}}{1000}\left( \frac{1}{B_{p}+2S_{p}}+\frac{1}{2S_{p}} \right)$ | $d_{eq}[m]$  $B_{p}\left[ mm \right]$  $H_{p}\left[ mm \right]$  $S_{p}[mm]$ | (Flagiello et al. 2021) |
| Schmidt number of the gas:  $Sc_{G}=\frac{\mu_{G}}{\rho_{G}D_{w,G}}$ | $\mu_{G}[kg/(m\cdot s)]$  $\rho_{G}[kg/m^{3}]$  $D_{w,G}\left[ m^{2}/s \right]$ | - |
| Viscosity of the gas mixture (equal air viscosity):  $\mu_{G}=\frac{0.001425T^{0.5039}}{1+\frac{108.3}{T}}$ | $T[C]$  $\mu_{G}[cP]$  $T[K]$ | DIPPR, 2024 (from Aspen Plus V12 data base) |
| Density of the gas mixture:  $\rho_{G}=\frac{PM_{air}}{RT}$ | $\rho_{G}[kg/m^{3}]$  $T\left[ K \right]$  $P\left[ Pa \right]$  $M_{air}\left[ \frac{kg}{kmol} \right]$ | Ideal gas |
| Enthalpy of air-water mixture (for the calculation of enthalpies at the inlet and outlet):  $H^{'}=\left( 1005+1884*Y^{'} \right)\left( T_{G} \right)+2502300*Y'$ | $H^{'}\left[ \frac{J}{kg dry air} \right]$  $Y^{'}\left[ \frac{kg water}{kg dry air} \right]$  $T_{G}[C]$ | (Treybal 1980) |
| Enthalpy of air-water mixture under saturation conditions (for the calculation of the saturation enthalpies in the calculation of the integral in Equation 2):  $H^{'*}=\left( 1005+1884*Y_{s}^{'} \right)\left( T_{L} \right)+2502300*Y_{s}^{'}$ | $H^{'*}\left[ \frac{J}{kg dry air} \right]$  $Y_{s}^{'}\left[ \frac{kg water}{kg dry air} \right]$  $T_{L}[C]$ | (Treybal 1980) |
| Enthalpy of air-water mixture (for the calculation of enthalpies in the calculation of the integral in Equation 2):  $H^{'}=4187\frac{L}{G_{s}}(T_{L}-T_{L,out})+H_{in}^{'}$ | $H^{'}\left[ \frac{J}{kg dry air} \right]$  $L[kg/s]$  $G_{s}[kg dry air/s]$  $T_{L}[C]$  $T_{L,out}[C]$  $H_{in}^{'}\left[ \frac{J}{kg dry air} \right]$ | (Treybal 1980) |
| Absolute humidity:  $Y^{'}=H_{r}Y_{s}^{'}$ | $Y^{'}\left[ \frac{kg water}{kg dry air} \right]$  $Y_{s}^{'}\left[ \frac{kg water}{kg dry air} \right]$  $H_{r}[\%]$ | (Treybal 1980) |
| Absolute humidity under saturation conditions:  $Y_{s}^{'}=\frac{0.622 p_{s}}{101325-p_{s}}$ | $Y_{s}^{'}\left[ \frac{kg water}{kg dry air} \right]$  $p_{s}[Pa]$ | (Treybal 1980) |
| Vapor pressure of pure water:  $\ln p_{s}=73.649-\frac{7258.2}{T}-7.3037*\ln T+4.1659\times{10}^{-6}*T^{2}$ | $p_{s}\left[ Pa \right]$  $T[K]$ | DIPPR, 2024 (from Aspen Plus V12 data base) |

**Table S7.** Parameters, variables and constants used in the calculation of the packing depth in the humidification.

| **Parameter, variable or constant** | **Value** | | | | **Reference** |
| --- | --- | --- | --- | --- | --- |
| $v_{G}[m/s]$ | 1.6 | | | | (Holmes and Keith 2012) optimized base case |
| $a[m^{2}/m^{3}]$ | 168 (210 m^2^/m^3^ with 80% efficiency) | | | |  |
| $B_{p}\left[ mm \right]$ | 24.1 | | | | (Flagiello et al. 2021)  Mellapack 250X |
| $H_{p}\left[ mm \right]$ | 11.9 | | | |  |
| $S_{p}[mm]$ | 17 | | | |  |
| $K_{G,h}[m/s]$ | 0.014 | | | | Calculated from Equation in Table S3 |
| $H_{tOG}[m]$ | 0.6111 | | | |  |
|  | **P1** | **P2** | **P3** | **P4** | Calculated form Aspen Simulation |
| Relative Humidity, $H_{r,out}$ | 93.94% | 91.78% | 97.88% | 93.94% |  |
| $G_{s} [kg/s]$ | 10583.8 | 18628.6 | 10583.8 | 10583.8 |  |
| $L [kg/s]$  (calculated in Aspen Plus) | 4000  (4079) | 6440  (7956) | 3737  (3813) | 4000  (4079) |  |
| $T_{Lout}[kg/s]$ | 9.457 | 9.591 | 8.841 | 9.457 |  |
| $H_{in}^{'}\left[ \frac{J}{kg dry air} \right]$ | 25252 | 25252 | 25252 | 25252 |  |
| $H_{out}^{'}\left[ \frac{J}{kg dry air} \right]$ | 27711 | 27301 | 28460 | 27711 |  |
| $Y_{in}^{'}\left[ \frac{kg water}{kg dry air} \right]$ | 0.0060299 | 0.0060299 | 0.0060299 | 0.0060299 |  |
| $Y_{out}^{'}\left[ \frac{kg water}{kg dry air} \right]$ | 0.0070053 | 0.0068424 | 0.0073023 | 0.0070053 |  |
| $N_{tOG}$ | 0.8523 | 0.6250 | 1.913 | 0.8523 | Equation 2 in Mauscript |
| $Z_{h}[m]$ | 0.5208 | 0.3819 | 1.169 | 0.5208 |  |
| $\Delta P[Pa]$ | 10.537 | 7.726 | 23.653 | 10.537 | (Holmes and Keith 2012) optimized base case |

# **Validation of Chemistry and ELECNRTL to calculate the solid**


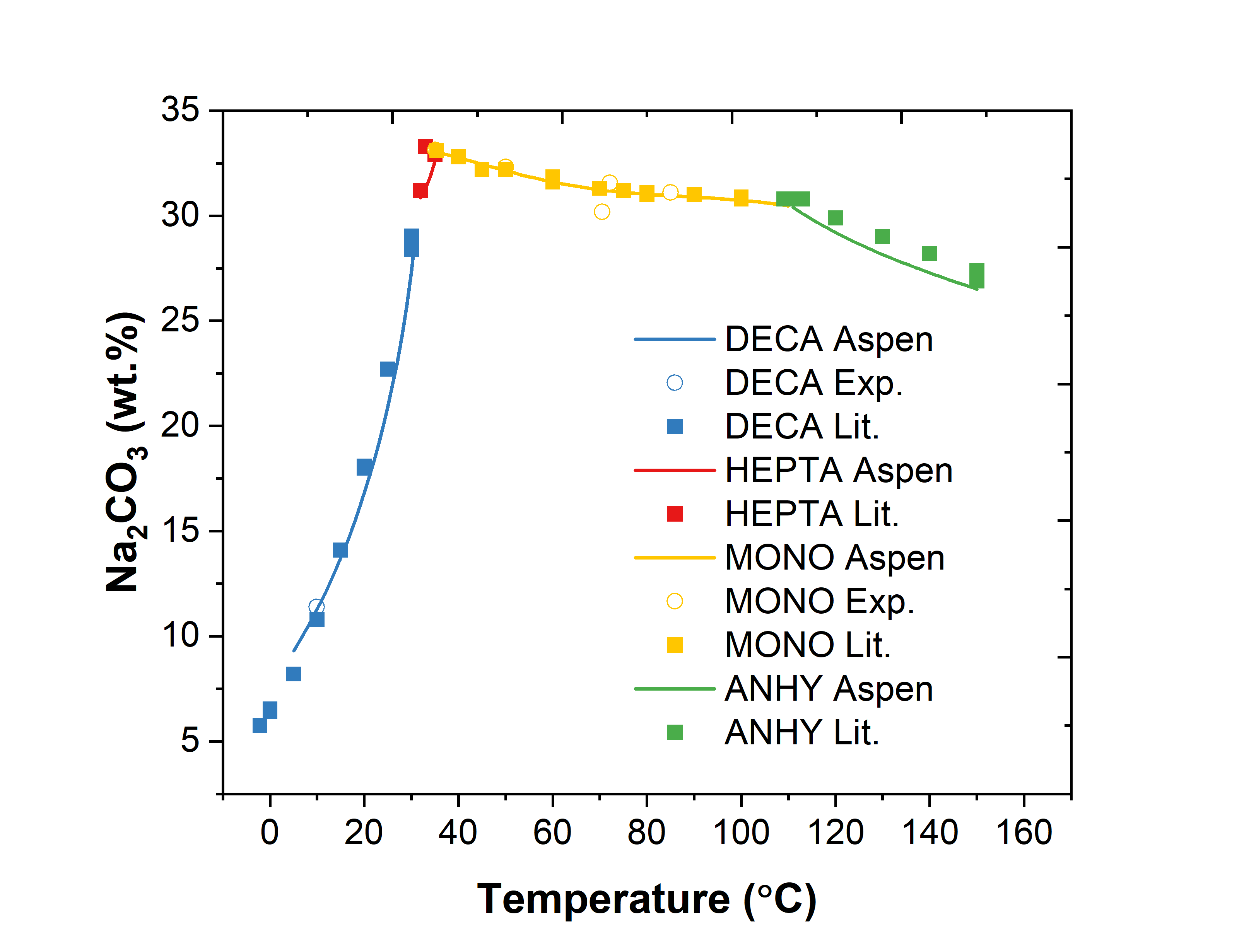


**Figure S1** Validation of the solid-liquid equilibria in the system Na_2_CO_3_-H_2_O. Exp: from(Ghaffari et al. 2025), Lit.: from various sources, Aspen: calculated with Aspen Plus V12.


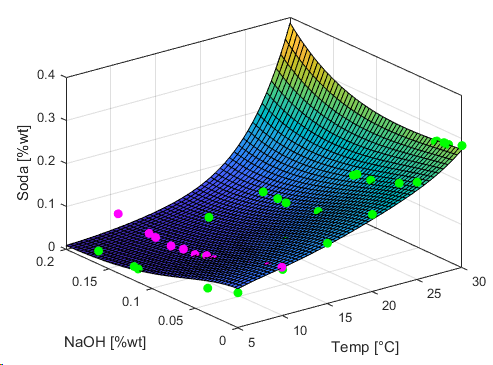


**Figure S2** Validation of the solid-liquid equilibria for sodium carbonate decahydrate in the system Na_2_CO_3_-NaOH-H_2_O. Pink dots: from (Ghaffari, 2024), Green dots: from various soures, Surface: calculated with ELECNRTL in Python using interaction parameters and equilibrium constants from Aspen Plus V12.


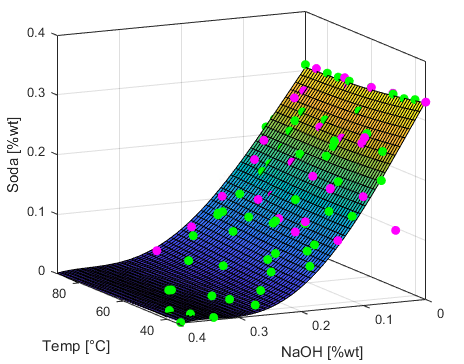

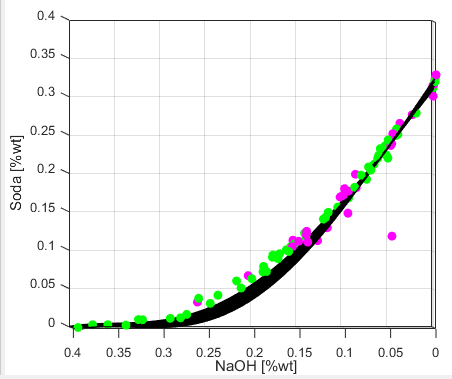


**Figure S3** Validation of the solid-liquid equilibria for sodium carbonate monohydrate in the system Na_2_CO_3_-NaOH-H_2_O. Pink dots: from (Ghaffari, 2024), Green dots: from various sources, Surface: calculated with ELECNRTL in Python using interaction parameters and equilibrium constants from Aspen Plus V12.

Sources of Literature data:

HOSTÁLEK, Z., ТРОЙНАЯ СИСТЕМА ВОДА-КАРБОНАТ НАТРИЯ-ГИДРАТ ОКИСИ НАТРИЯ. Collection of Czechoslovak Chemical Communications, 1957. 22(2): p. 532.

Tranquard, A., Le systéme ternaire Eau-Soude-Carbonate de sodium Étude dans l'intervalle de température 60-100 C. Bulletin de la Société Chimique de France, 1964(2): p. 264.

Freeth, F.A., II. The system: Na2O—CO2—NaCl—H2O, considered as two four-component systems. Philosophical Transactions of the Royal Society of London. Series A, Containing Papers of a Mathematical or Physical Character, 1923. 223(605-615): p. 35-87.

Itkina, L.S., Izoterma Rastvorimosti Sistemy Na2SO4-Na2CO3-Nacl-NaOH-H2O Pri 50-Degrees-C. Zhurnal Prikladnoi Khimii, 1949. 22(3): p. 278-289.

Itkina, L.S., Rastvorimost V Sisteme Na2SO4-Na2CO3-Nacl-NaOH-H2O Pri 100-Degrees. Zhurnal Prikladnoi Khimii, 1953. 26(5): p. 495-499.

Itkina, L.S. and N.M. Chaplygina, Izoterma-50-Gradusov Rastvorimosti V Sisteme 2Li+, 2Na+-Paralle-to-CO3(2-), 20H(-)+H2O. Zhurnal Neorganicheskoi Khimii, 1963. 8(6): p. 1479-1488.

Klyashtorny M. I., O.E.D.I., Sorption of water vapor by the system Na2CO3-NaOH. Zhurnal Prikladnoi Khimii, 1962. 35(3).

Pischinger, E., Szufarski, Z., BADANIE UKLADU Na2SO4-Na3CO3-NaOH-H2O W TEMPERATURZE 30 °C. Chemia Stosowana, 1958. 2(2): p. 247.

Szufarski Z., P.E., Badania rozpuszczalnosci weglanu, siarczanu i chlorku sodowegow roztworach wodortlenku sodowego. Przemysł Chemiczny, 1962. 41( 12): p. 695

# **Initial mass balances**

For the absorption-crystallization loop in processes P1 and P4 the block diagram in Figure S4 was used to obtain Equations (S1) to (S4). In the block diagram, the flow of water evaporated in the decahydrate crystallizer, used to cool down the mixture from the absorption temperature ($T_{1}$) to the crystallization temperature ($T_{2}$) is not included because it is completely returned to the feed of the absorption. In addition, the concentration of the NaOH solution entering the absorption-crystallization loop was adjusted so that the inlet flow water to the loop was equal to the flow of water exiting in the decahydrate crystals minus the water produced stoichiometrically from the reaction. The crystallization temperature was defined from a desired supersaturation in the crystallizer ($S$), which was calculated as the saturation concentration at $T_{2}$ over the saturation concentration at $T_{1}$: $S=\frac{w_{Na_{2}CO_{3}}^{sat}\left( at T_{2} \right)}{w_{Na_{2}CO_{3}}^{sat}\left( at T_{1} \right)}=1.05$.

**Figure S4** Simplified block flow diagram of the absorption-decahydrate crystallization loop used in processes P1 and P4. Purple line: recycling stream. Dashed red line: control volume for mass balances.

The global mass balance in the mixer M1 is presented in Equation (S1), where $\dot{F}_{NaOH}$ is the mass flow rate of sodium hydroxide stoichiometrically required to achieve the soda ash production rate ($\dot{F}_{NaOH}=31447.6 kg/h$ ), $W_{deca}$ is the mass flow rate of water that exits the loop in the decahydrate crystals ($\dot{W}_{deca}=112489 kg/h$) and $\dot{F}_{w}$ is the mass flow rate of water stoichiometrically produced by the reaction in the absorber ($\dot{F}_{w}=7082.24 kg/h$). A first estimation of the recirculation flow rate is given when $\dot{L}_{R}$is calculated from Equation (S12).

| $\dot{F}_{NaOH}+\dot{W}_{deca}-\dot{F}_{w}+\dot{L}_{R}=\dot{L}_{in}$ | (S1) |
| --- | --- |

Equations (S2), (S3) and (S4) represent the balance of NaOH, Na_2_CO_3_ and water in the red control volume in Figure S4. The flow of sodium carbonate out of the red control volume ($\dot{F}_{Na_{2}CO_{3}}$) corresponds to the desired production rate. Combining Equations (S2) and (S3) results in Equation (S5), which when combined with Equation (S4) and with the sum equation of the liquid out of the absorber results in Equation (S6).

| $w_{NaOH}^{LOUT}\dot{L}_{out}=w_{NaOH}^{LR}\dot{L}_{R}$ | (S2) |
| --- | --- |
| $w_{Na_{2}CO_{3}}^{LOUT}\dot{L}_{out}=\dot{F}_{Na_{2}CO_{3}}+w_{Na_{2}CO_{3}}^{LR}\dot{L}_{R}$ | (S3) |
| $w_{w}^{LOUT}\dot{L}_{out}=\dot{W}_{deca}+w_{w}^{LR}\dot{L}_{R}$ | (S4) |
| $w_{Na_{2}CO_{3}}^{LOUT}\frac{w_{NaOH}^{LR}}{w_{NaOH}^{LOUT}}\dot{L}_{R}=\dot{F}_{Na_{2}CO_{3}}+w_{Na_{2}CO_{3}}^{LR}\dot{L}_{R}$ | (S5) |
| $\left( \frac{w_{NaOH}^{LR}}{w_{NaOH}^{LOUT}}-1 \right)\dot{L}_{R}=\dot{W}_{deca}+\dot{F}_{Na_{2}CO_{3}}$ | (S6) |

For the estimation of the concentrations in the streams in the loop, the NaOH concentration in the outlet of the absorber ($w_{NaOH}^{LOUT}$) should be specified (degree of freedom in the process design). This concentration was fixed according to the optimal concentration found for the CO_2_ absorption in the soda ash production (Ghaffari et al. 2023). Then, the concentration of NaOH in the recycling stream ($w_{NaOH}^{LR}$) can be estimated with Equation (S6). The concentration of Na_2_CO_3_ of the recycling stream ($w_{Na_{2}CO_{3}}^{LR}$) is calculated from the solid-equilibrium equations (ELECNRTL used as activity model), which can be represented as a function of the temperature $T_{2}$ and the NaOH concentration $w_{NaOH}^{LR}$. Here it is assumed that the liquid out of the filter (liquid in the crystallizer) is at the saturation condition and that the filtration is perfect, so that the crystals are completely dry at the outlet of the filter. Both assumptions are valid in this conceptual process design study, in which the goal is to compare different process alternatives.

For the absorption-crystallization loop in process P2, the block diagram in Figure S5 was used to obtain Equations (S7) to (S10), used to estimate the flow and composition of the recycling stream (LR). Here the main difference with the previously presented equations is that the concentration of Na_2_CO_3_ of the recycling stream ($w_{Na_{2}CO_{3}}^{LR}$) is calculated at the same temperature of the absorption ($T_{1}$). In addition, the flows of streams LIN* ($\dot{L}_{in}^{*}$) and LOUT* ($\dot{L}_{out}^{*}$) were calculated from splitting ratio in S3 and the flow rate or liquid required in the absorber ($\dot{L}_{in}$). Combining Equations (S19) and (S20) results in Equation (S22), which when combined with Equation (S21) and with the sum equation of the liquid out of the absorber results in Equation (S22). the concentration of NaOH in the recycling stream ($w_{NaOH}^{LR}$) can be estimated with Equation (S23).

**Figure S5** Simplified block flow diagram of the absorption-decahydrate crystallization loop used in process P2. Purple line: recycling stream. Dashed red line: control volume for mass balances.

| $\dot{F}_{NaOH}+\dot{W}_{deca}-\dot{F}_{w}+\dot{L}_{R}=\dot{L}_{in}^{*}$ | (S7) |
| --- | --- |
| $w_{NaOH}^{LOUT^{*}}\dot{L}_{out}^{*}=w_{NaOH}^{LR}\dot{L}_{R}$ | (S8) |
| $w_{Na_{2}CO_{3}}^{LOUT^{*}}\dot{L}_{out}^{*}=\dot{F}_{Na_{2}CO_{3}}+w_{Na_{2}CO_{3}}^{LR}\dot{L}_{R}$ | (S9) |
| $w_{w}^{LOUT^{*}}\dot{L}_{out}^{*}=\dot{W}_{deca}+w_{w}^{LR}\dot{L}_{R}$ | (S10) |
| $w_{Na_{2}CO_{3}}^{LOUT^{*}}\frac{w_{NaOH}^{LR}}{w_{NaOH}^{LOUT^{*}}}\dot{L}_{R}=\dot{F}_{Na_{2}CO_{3}}+w_{Na_{2}CO_{3}}^{LR}\dot{L}_{R}$ | (S11) |
| $\left( \frac{w_{NaOH}^{LR}}{w_{NaOH}^{LOUT^{*}}}-1 \right)\dot{L}_{R}=\dot{W}_{deca}+\dot{F}_{Na_{2}CO_{3}}$ | (S12) |

For the absorption-crystallization loop in process P3, the block diagram in Figure S6 was used to obtain Equations (S13) to (S18), used to estimate the flow and composition of the recycling stream (LR). In Equation (S13), $\dot{F}_{X}$ is the flow of water evaporated in the monohydrate crystallizer, which was adjusted to ensure that the inlet liquid to the absorber was undersaturated in sodium carbonate (approx. 50% of the saturation concentration). In Equation (S16), $\dot{W}_{mono}$ is the flow of water that exits the loop in the monohydrate crystals (7082.24 kg/h), which due to the stoichiometry is equal to the water produced by the reaction in the absorber. As in the process P2, the flows of streams LIN* ($\dot{L}_{in}^{*}$) and LOUT* ($\dot{L}_{out}^{*}$) were calculated from splitting ratio in S3 and the flow rate or liquid required in the absorber ($\dot{L}_{in}$). As mentioned in the main manuscript, the splitting ratio in S3 was adjusted to obtain the desired magma density. Combining Equations (S14) and (S15) results in Equation (S17), which when combined with Equation (S16) and with the sum equation of the liquid out of the absorber results in Equation (S18). The concentration of NaOH in the recycling stream ($w_{NaOH}^{LR}$) can be estimated with Equation (S18).

| $\dot{F}_{NaOH}+\dot{F}_{X}+\dot{L}_{R}=\dot{L}_{in}^{*}$ | (S13) |
| --- | --- |
| $w_{NaOH}^{LOUT^{*}}\dot{L}_{out}^{*}=w_{NaOH}^{LR}\dot{L}_{R}$ | (S14) |
| $w_{Na_{2}CO_{3}}^{LOUT^{*}}\dot{L}_{out}^{*}=\dot{F}_{Na_{2}CO_{3}}+w_{Na_{2}CO_{3}}^{LR}\dot{L}_{R}$ | (S15) |
| $w_{w}^{LOUT^{*}}\dot{L}_{out}^{*}=\dot{F}_{X}+\dot{W}_{mono}+w_{w}^{LR}\dot{L}_{R}$ | (S16) |
| $w_{Na_{2}CO_{3}}^{LOUT^{*}}\frac{w_{NaOH}^{LR}}{w_{NaOH}^{LOUT^{*}}}\dot{L}_{R}=\dot{F}_{Na_{2}CO_{3}}+w_{Na_{2}CO_{3}}^{LR}\dot{L}_{R}$ | (S17) |
| $\left( \frac{w_{NaOH}^{LR}}{w_{NaOH}^{LOUT^{*}}}-1 \right)\dot{L}_{R}=\dot{F}_{X}+\dot{W}_{mono}+\dot{F}_{Na_{2}CO_{3}}$ | (S18) |

**Figure S6** Simplified block flow diagram of the absorption-monohydrate crystallization loop used in process P3. Green line: high purity water. Orange line: recycling stream. Dashed red line: control volume for mass balances.

# **Equipment cost correlation and total investment cost equation**

**Table S8.** Correlations used to estimate the purchase equipment cost in the CODA process.

| **Equipment** | **Purchase cost equation** | **Size factor** | **Ref.** |
| --- | --- | --- | --- |
| Electrolysis | $C_{P,Electrolysis}=30000A_{e}$ | Cell area electrolysis, $A_{e}[m^{2} ]$ | (Ramdin et al. 2019) |
| Cross-flow packed absorber | $C_{p}=\left( 3700+250D \right)A_{in}\left( \frac{I_{2023}}{I_{2009}} \right)$ | Packing depth, $D[m]$  Air inlet area, $A_{in}[m^{2}]$ | (Holmes and Keith 2012) |
| Droplet absorber | $C_{p}=\left( F_{M}C_{V}+C_{PL} \right)\left( \frac{I_{2023}}{I_{b}} \right)$  $C_{V}=\exp(7.0374+0.18255\ln W+0.02297\left( \ln W \right)^{2})$  $C_{PL}=237.1D^{0.63319}H^{0.80161}$  $W=\pi\left( D_{i}+t_{s} \right)\left( H_{i}+0.8D \right)t_{s}0.284$  Material factor, $F_{M}=2.1$ for stainless steel | Vessel mass, $W[lb]$  Vessel diameter ^a^, $D\left[ ft \right], D_{i}[in]$  Vessel height, $H\left[ ft \right],H_{i}[in]$  Shell thickness, $t_{s}[in]$ | (Seider, Seader, and Lewin 2004) |
| Centrifugal pump and motor | $C_{p}=(F_{T}C_{b,motor}+F_{T}F_{M}C_{b,pump})\left( \frac{I_{2023}}{I_{b}} \right)$  $C_{b,pump}=N\exp\left( 9.2951-0.6019{\ln S}_{max}+0.0519\left( \ln S_{max} \right)^{2} \right)$  $C_{b,motor}=N\exp\left( 5.4866+0.1314\ln P_{C,i}+0.053255\left( \ln P_{C,i} \right)^{2}+0.028628\left( \ln P_{C,i} \right)^{3}-0.0035549\left( \ln P_{C,i} \right)^{4}) \right)$  $S=QH^{0.5}$  Material factor, $F_{M}=2$ for stainless steel  Function type factor, $F_{T}=8.9$ for the head and flow range used  Maximal size, $S_{max}=100000 gpm*ft^{0.5}$ | Size pump, $S[gpm*ft^{0.5}]$  Volumetric flow, $Q[gpm]$  Pump head, $H[ft]$  Number of pumps of maximal size, $N[-]$  Power consumption for each pump, $P_{C,i}[hp]$ |  |
| Absorber fan (tube-axial) | $C_{p}=F_{M}C_{b}\left( \frac{I_{2023}}{I_{b}} \right)$  $C_{b}=N\exp\left( 5.89085-0.40254{\ln Q}_{max}+0.05787\left( \ln Q_{max} \right)^{2} \right)$  Material factor, $F_{M}=2.5$ for stainless steel  Maximal volumetric flow, $Q_{max}=800000 ft^{3}/h$ | Volumetric flow, $Q[ft^{3}/h]$  Number of fans of maximal size, $N[-]$ |  |
| Crystallizers | $C_{p}=F_{M}N(27500{W_{max}}^{0.56})\left( \frac{I_{2023}}{I_{b}} \right)$  Maximal flow of crystals, $W_{max}=1000 ton/day$ | Flow of crystals, $W[ton/day]$  Number of crystallizers of maximal size, $N[-]$ |  |
| Compressors | $C_{p}=F_{M}F_{T}C_{b}\left( \frac{I_{2023}}{I_{b}} \right)$  $C_{b}=\exp(7.7661+0.7243\ln P_{C})$  Material factor, $F_{M}=2.5$ for stainless steel  Function type factor, $F_{T}=1.15$ for steam turbine | Power consumption, $P_{C}[hp]$ |  |
| MVR-Blowers | $C_{p}=F_{M}C_{b}\left( \frac{I_{2023}}{I_{b}} \right)$  $C_{b}=\exp(6.6547+0.7900\ln P_{C})$  Material factor, $F_{M}=2.5$ for stainless steel | Power consumption, $P_{C}[hp]$ |  |
| Hydrocyclone | $C_{p}=F_{M}C_{b}\left( \frac{I_{2023}}{I_{b}} \right)$  $C_{b}=190Q^{0.5}$  Material factor, $F_{M}=2$ for stainless steel | Liquid volumetric flow, $Q[gpm]$ |  |
| Total Capital Investment TCI (Study Estimate ±35%) | Based on the Overall Factor Method of Lang, 1947 a,b, and 1948  $C_{TCI}=1.05{f_{L}}_{TCI}\sum_{i} {C_{p}}_{i}+{C_{P}}_{Electrolysis}$  $i=2-9$, running number for main equipment listed in this table, except Electrolysis, which cost function is based on TCI of real life plants | Recommended Lang Factor of Peters and Timmerhaus, including working capital for a solids-liquids processing plant,  ${f_{L}}_{TCI}=4.9$ |  |

^a^ Calculated from absorber volume assuming a cylindrical vessel and using a factor of 1.2 to overestimate the diameter.

# **Aspen Plus simulation screenshots**


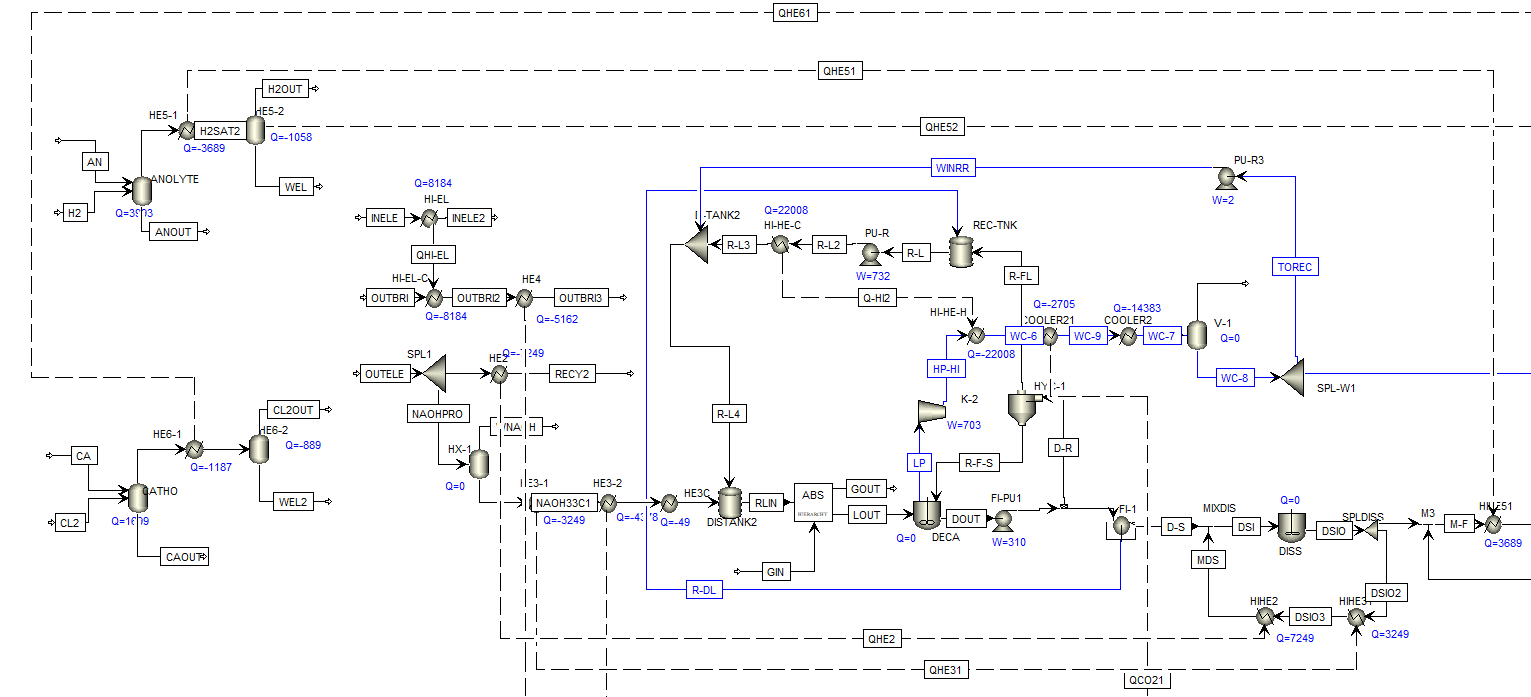

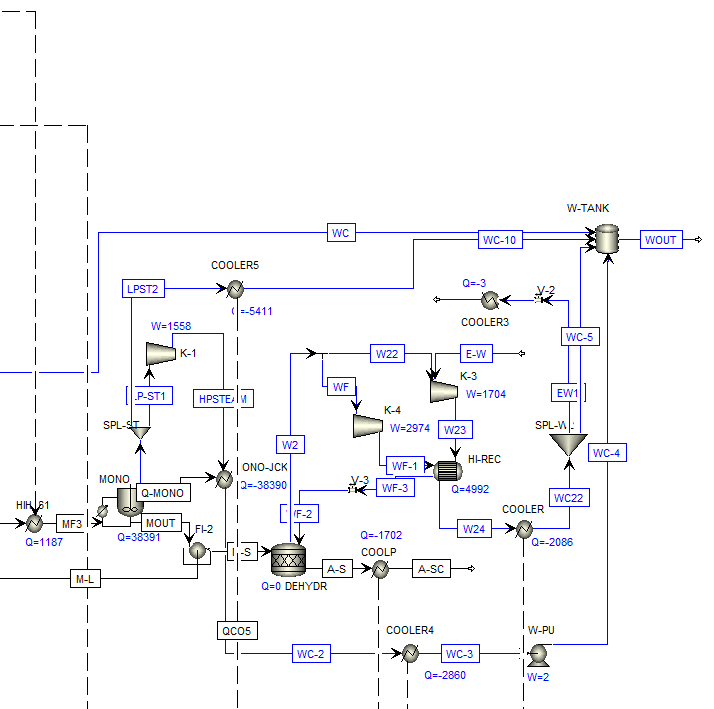


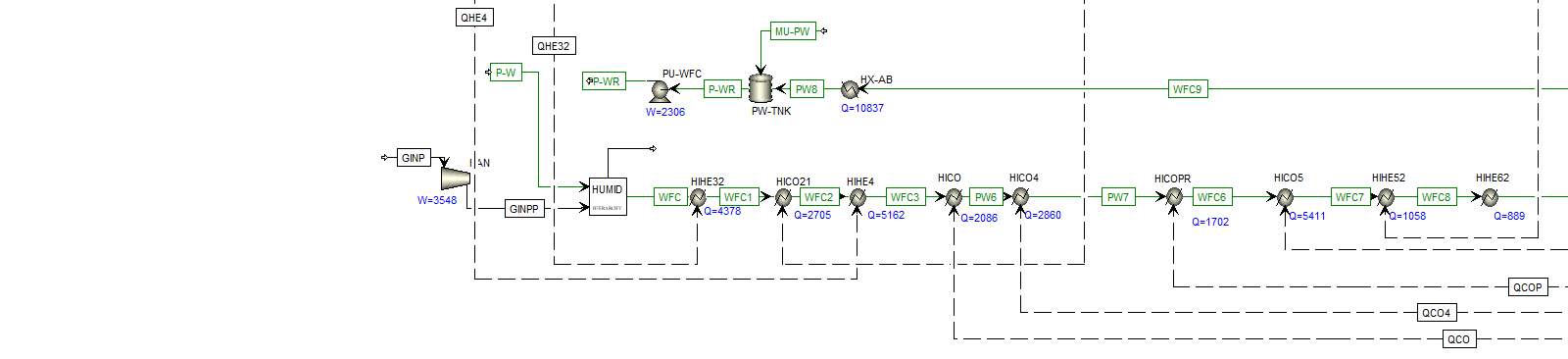

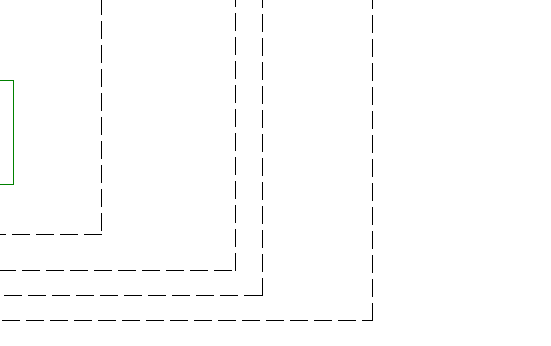


**Figure S7** Screenshot of main flowsheet in Aspen Plus V12 for process alternative P1.


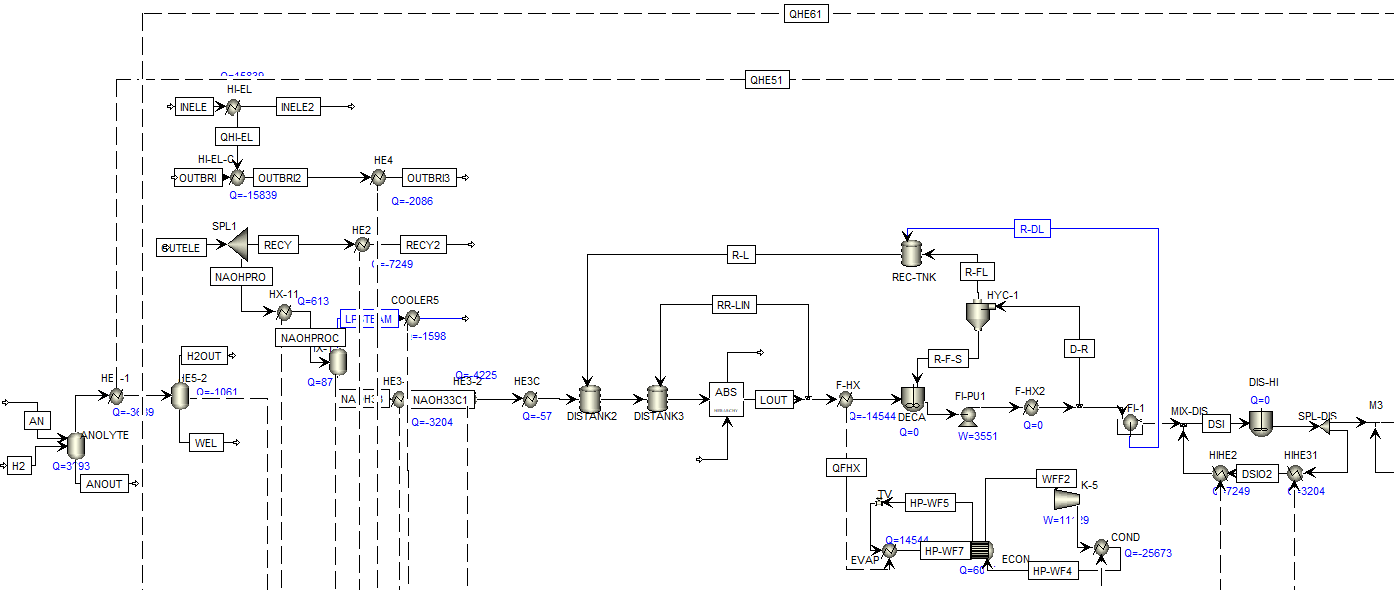

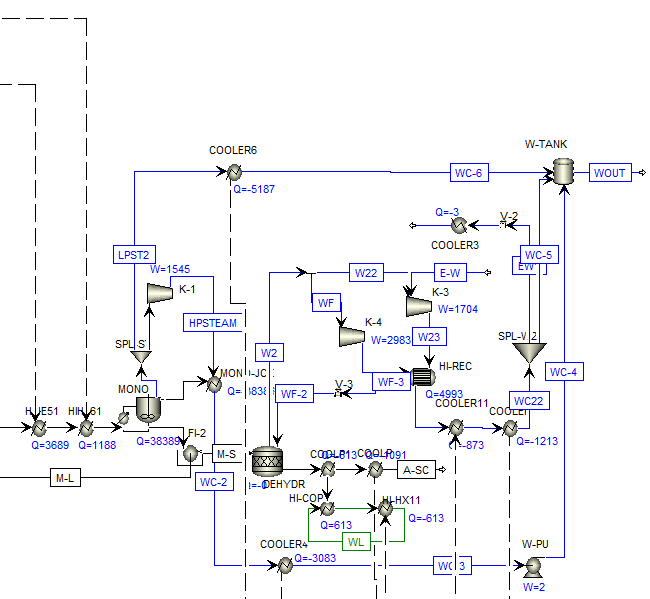


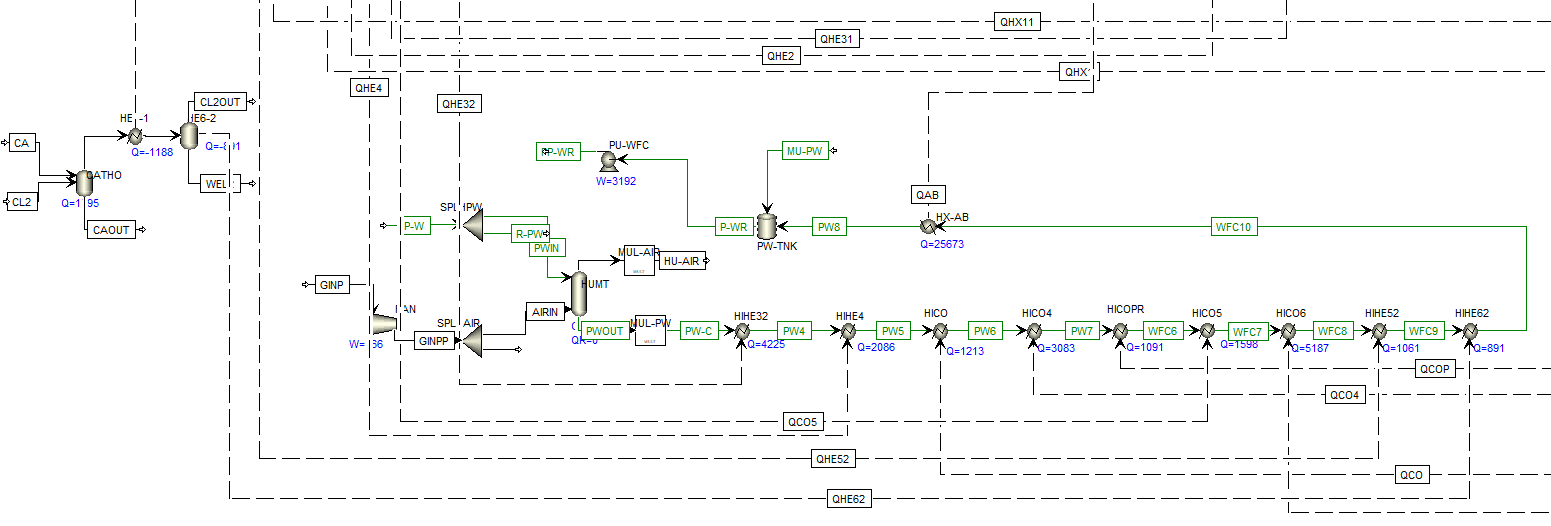

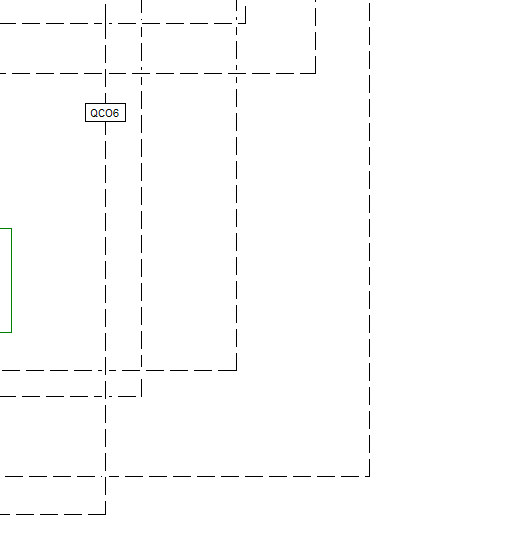


**Figure S8** Screenshot of main flowsheet in Aspen Plus V12 for process alternative P2.


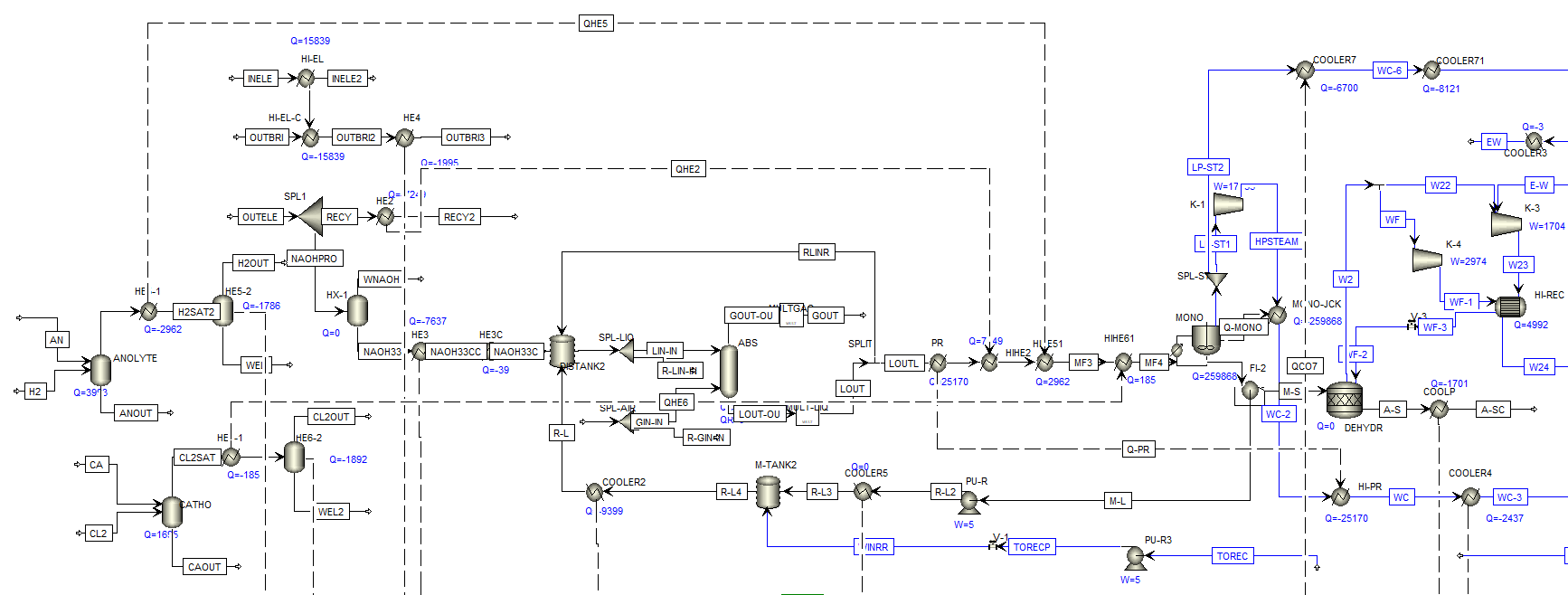

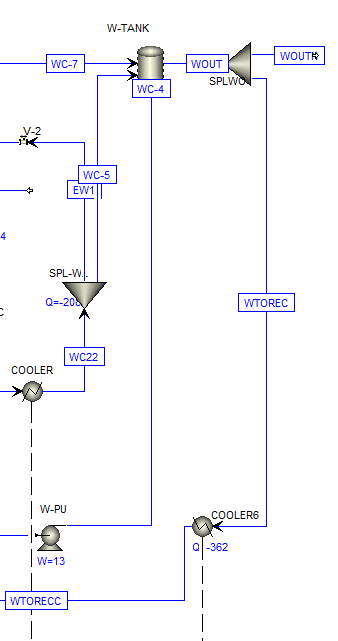


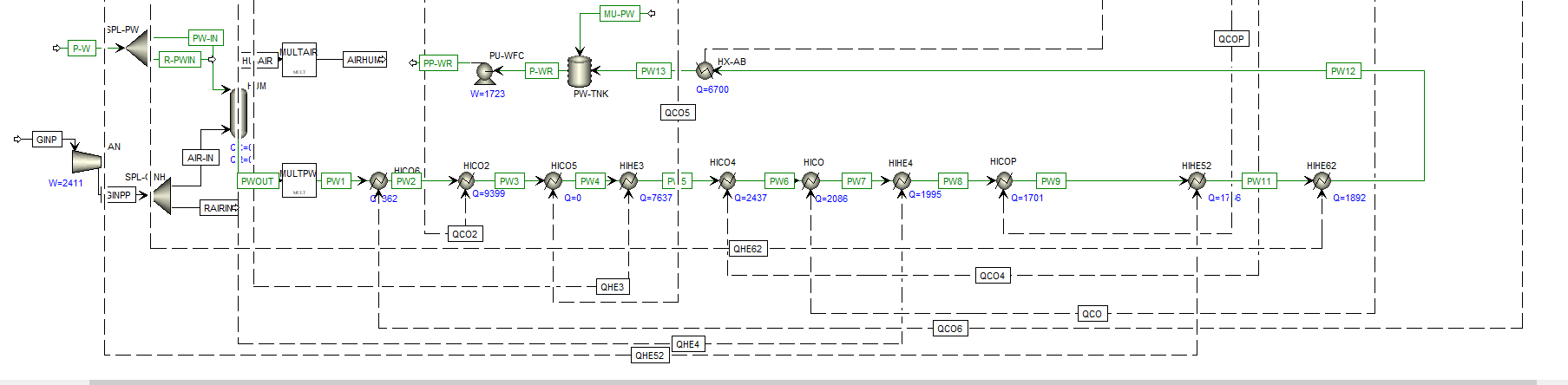


**Figure S9** Screenshot of main flowsheet in Aspen Plus V12 for process alternative P3.


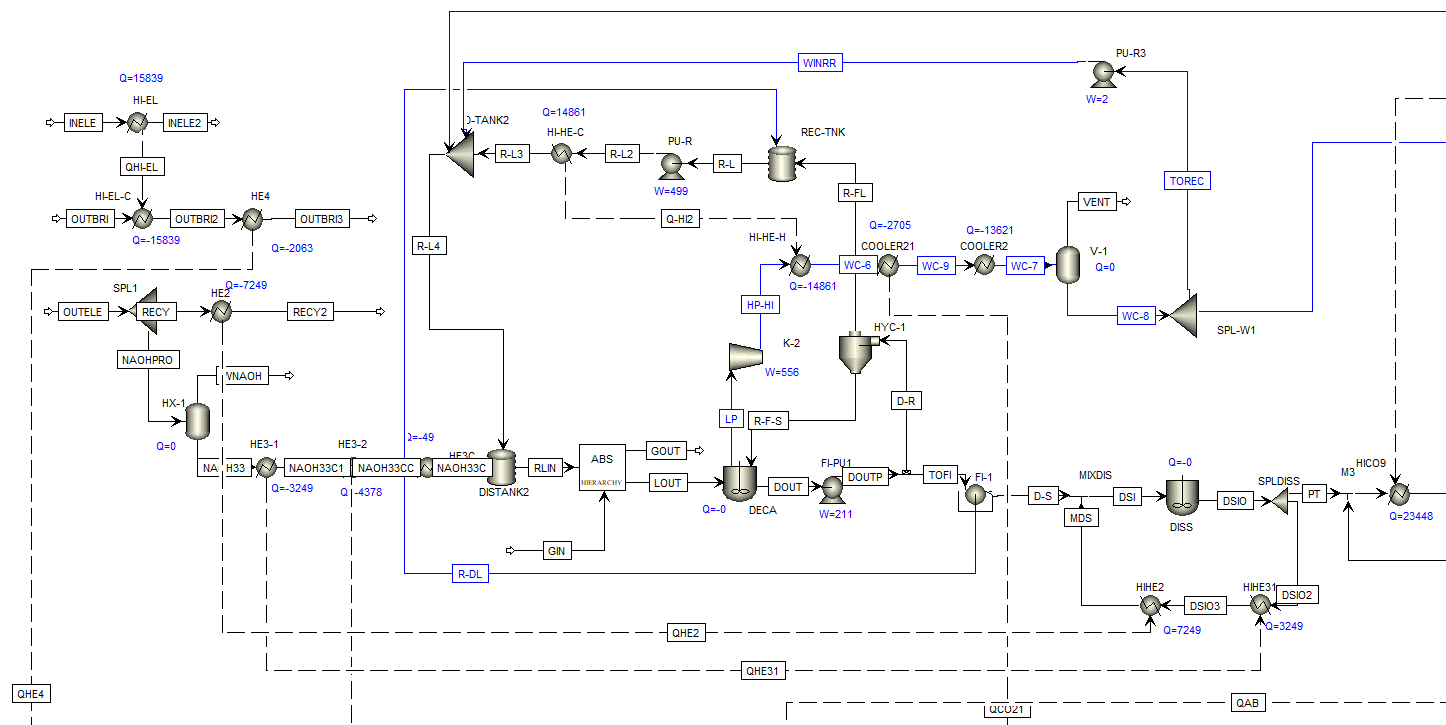

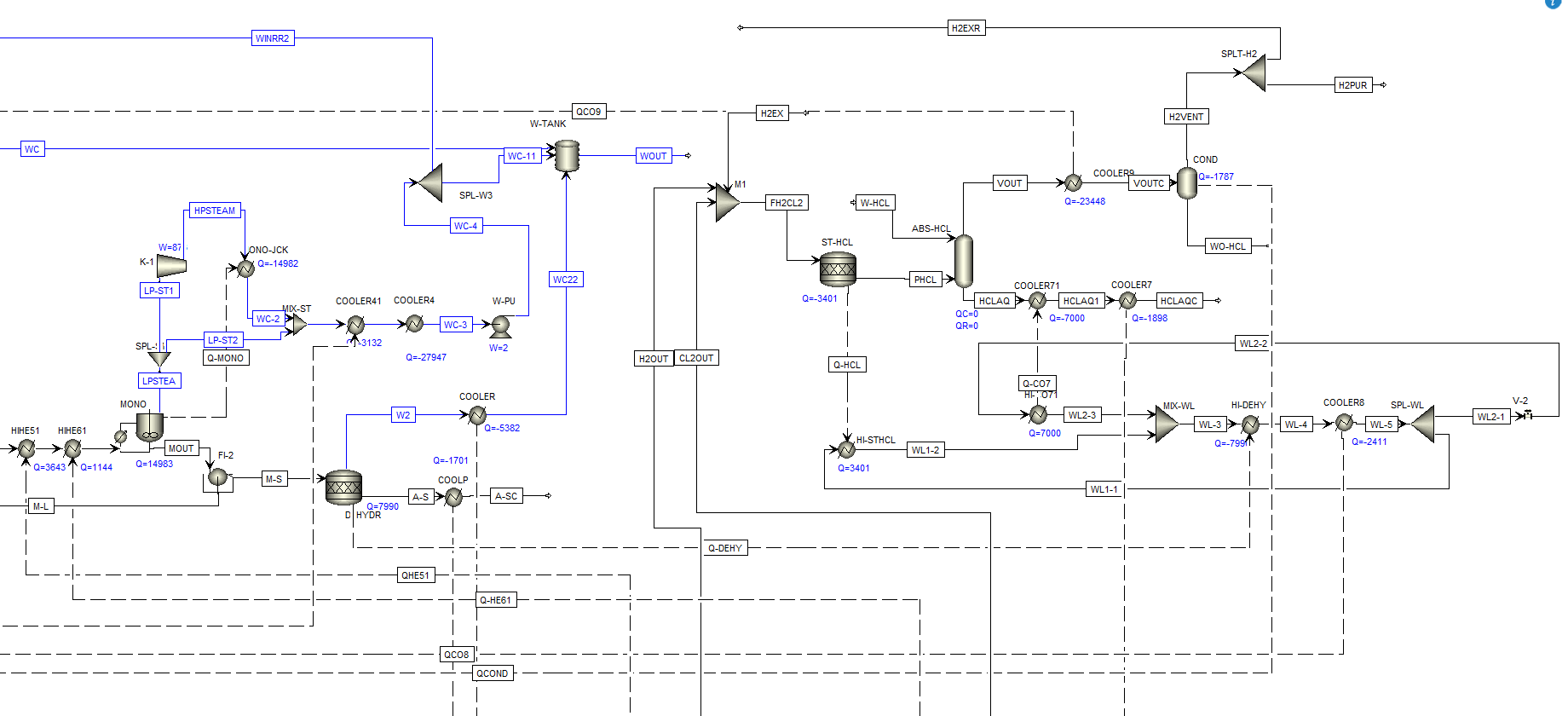


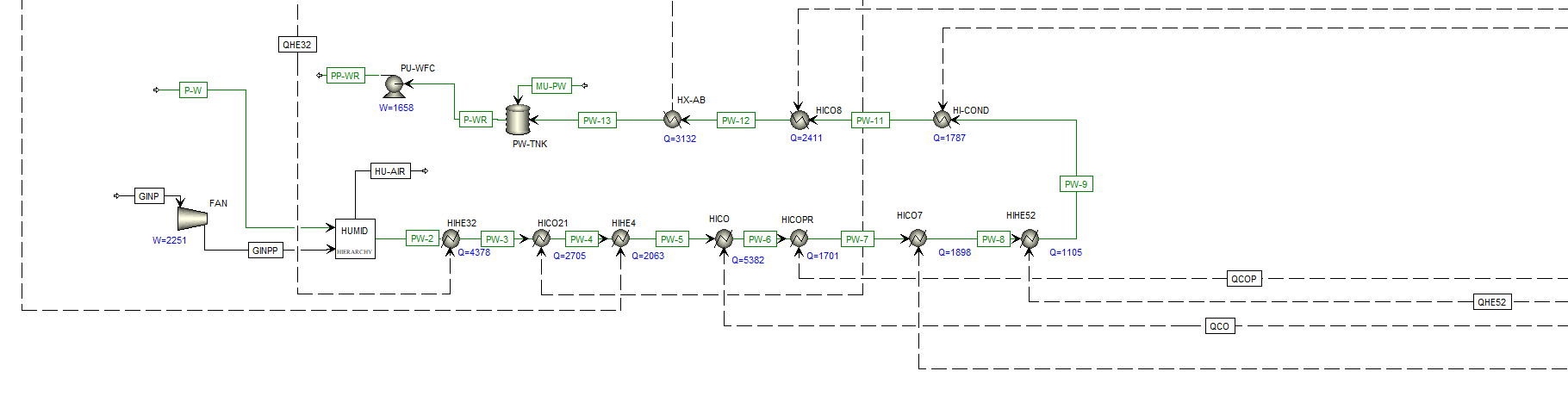

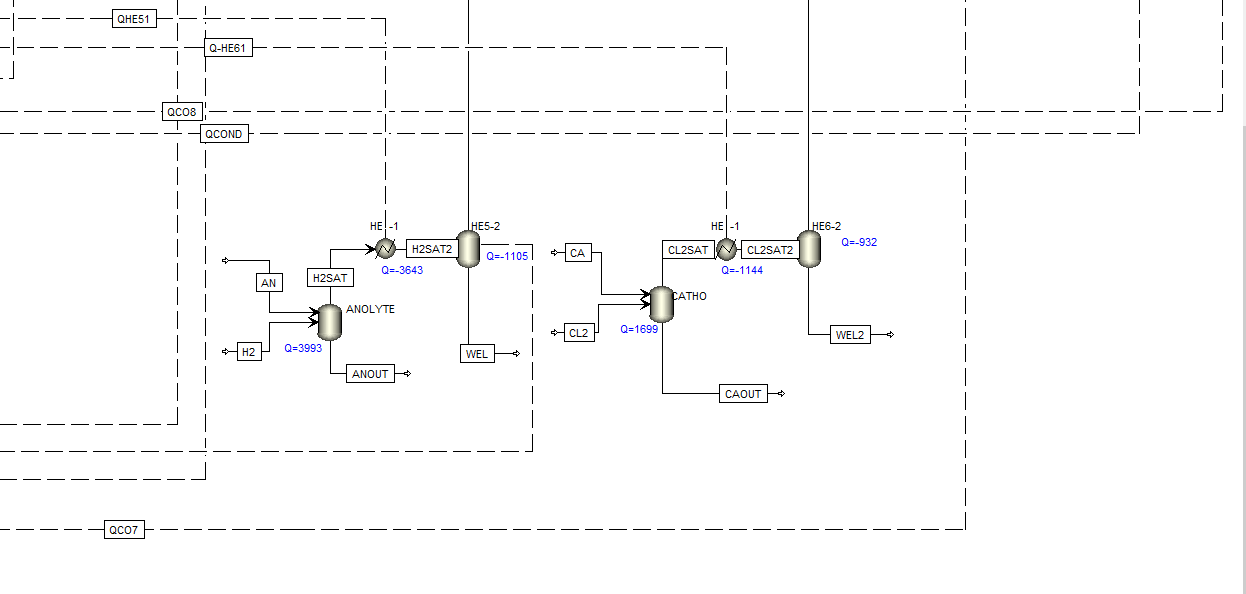


**Figure S10** Screenshot of main flowsheet in Aspen Plus V12 for process alternative P4.

# **Other results**

**Table S9.** Results details on the heat exchangers for the process alternative P1.

| **Heat exchanger** | **Name in Aspen plus** | **Heat transfer coefficient, W/m2C** | **Heat, kW** | **Tin, °C** | **Tout, °C** | **P, bar** | **Tutility in, °C** | **Tutility out, °C** | **LMDT, °C** | **Area, m2** | **dT1, C** | **dT2, C** |
| --- | --- | --- | --- | --- | --- | --- | --- | --- | --- | --- | --- | --- |
| HE13 | COOLER | 850 | -2085.84 | 204.99 | 11.00 | 20.00 | 9.99 | 10.11 | -36.83 | 66.63 | -194.88 | -1.01 |
| HE10 | MONO-JCK | 1500 | -38387.60 | 102.97 | 55.00 | 0.16 | 50.13 | 50.00 | -20.16 | 1269.25 | -52.97 | -4.88 |
| HE14 | COOLP | 850 | -1700.92 | 150.00 | 11.30 | 1.00 | 10.28 | 10.38 | -28.17 | 71.04 | -139.62 | -1.02 |
| HE7 | HI-HE-H | 1500 | -14795.20 | 31.22 | 11.00 | 0.01 | 9.24 | 10.00 | -7.81 | 1262.14 | -21.22 | -1.76 |
| HE11 | COOLER4 | 850 | -2853.06 | 55.00 | 11.20 | 0.16 | 10.11 | 10.28 | -11.74 | 286.01 | -44.72 | -1.09 |
| HE3-2 | HE3-2 | 850 | -4377.84 | 55.93 | 10.50 | 1.00 | 9.46 | 9.71 | -11.92 | 432.09 | -46.22 | -1.04 |
| HE4 | HE4 | 850 | -2063.02 | 20.00 | 10.90 | 1.00 | 9.87 | 9.99 | -3.95 | 614.95 | -10.01 | -1.03 |
| HE1 | HI-EL | 850 | 15839.00 | 10.00 | 63.00 | 1.00 | 90.00 | 20.00 | 17.11 | 1088.82 | 10.00 | 27.00 |
| HE2 | HE2 | 850 | -7248.69 | 90.00 | 75.00 | 1.00 | 51.56 | 55.00 | -28.84 | 295.71 | -35.00 | -23.44 |
| HE3-1 | HE3-1 | 850 | -3249.27 | 90.00 | 55.93 | 1.00 | 50.00 | 51.56 | -17.40 | 219.75 | -38.44 | -5.93 |
| HE8-1 | COOLER21 | 1500 | -2705.08 | 11.00 | 10.99 | 0.01 | 9.71 | 9.87 | -1.20 | 1501.90 | -1.13 | -1.28 |
| HE5-1 | HE5-1 | 50 | -3689.40 | 90.00 | 55.00 | 1.28 | 49.97 | 50.09 | -16.84 | 4381.72 | -39.91 | -5.03 |
| HE6-1 | HE6-1 | 50 | -1186.78 | 90.00 | 55.00 | 1.30 | 50.09 | 50.13 | -16.69 | 1421.72 | -39.87 | -4.91 |
| HE9 | COOLER5 | 1500 | -5409.91 | 50.00 | 11.40 | 0.10 | 10.38 | 10.70 | -10.48 | 344.13 | -39.30 | -1.02 |
| HE3-3 | HE3C | 850 | -49.03 | 10.50 | 10.00 | 1.00 | 5.00 | 8.00 | -3.61 | 15.99 | -2.50 | -5.00 |
| HE8-2 | COOLER2 | 1500 | -13667.80 | 10.99 | 10.00 | 0.01 | 5.00 | 8.00 | -3.91 | 2331.79 | -2.99 | -5.00 |
| ignored | COOLER3 | 850 | -3.35 | 11.44 | 10.00 | 1.00 | 5.00 | 8.00 | -4.17 | 0.95 | -3.44 | -5.00 |
| HE15 | HX-AB | 850 | 3182.64 | 10.81 | 11.00 | 1.00 | 20.00 | 15.00 | 6.29 | 595.29 | 4.19 | 9.00 |
| HE5-2 | HE5-2 | 50 | -1055.37 | 55.00 | 11.70 | 1.28 | 10.70 | 10.76 | -11.41 | 1849.38 | -44.24 | -1.00 |
| HE6-2 | HE6-2 | 50 | -887.14 | 55.00 | 11.80 | 1.30 | 10.76 | 10.81 | -11.51 | 1541.99 | -44.19 | -1.04 |
| HE12 | HI-REC | 850 | 4991.56 | 212.44 | 204.99 | 20.00 | 168.85 | 200.00 | -22.22 | 264.25 | -12.44 | -36.14 |

**Table S10.** Results details on the heat exchangers for the process alternative P2.

| **Heat exchanger** | **Name in Aspen plus** | **Heat transfer coefficient, W/m2C** | **Heat, kW** | **Tin, °C** | **Tout, °C** | **P, bar** | **Tutility in, °C** | **Tutility out, °C** | **LMDT, °C** | **Area, m2** | **dT1, C** | **dT2, C** |
| --- | --- | --- | --- | --- | --- | --- | --- | --- | --- | --- | --- | --- |
| HE13 | COOLER | 850 | -1212.81 | 125.55 | 10.90 | 20.00 | 9.80 | 9.84 | -24.60 | 58.00 | -115.71 | -1.10 |
| HE10 | MONO-JCK | 1500 | -38386.20 | 102.34 | 55.00 | 0.16 | 50.13 | 50.00 | -19.99 | 1280.04 | -52.33 | -4.87 |
| HE14 | COOLP | 850 | -1091.19 | 100.00 | 11.02 | 1.00 | 9.93 | 9.97 | -20.14 | 63.74 | -90.03 | -1.09 |
| HE8 | COOLP1 | 850 | -613.16 | 150.00 | 100.00 | 1.00 | 95.00 | 99.63 | -19.64 | 36.73 | -50.37 | -5.00 |
| HE11 | COOLER4 | 850 | -3083.03 | 55.00 | 11.00 | 0.16 | 9.84 | 9.93 | -12.00 | 302.33 | -45.07 | -1.16 |
| HE3-2 | HE3-2 | 850 | -4225.44 | 55.57 | 10.60 | 1.00 | 9.61 | 9.74 | -11.68 | 425.59 | -45.83 | -0.99 |
| HE4 | HE4 | 850 | -2085.76 | 20.00 | 10.80 | 1.00 | 9.74 | 9.80 | -4.03 | 608.25 | -10.19 | -1.06 |
| HE1 | HI-EL | 850 | 15839.00 | 10.00 | 63.00 | 1.00 | 90.00 | 20.00 | 17.11 | 1088.82 | 10.00 | 27.00 |
| HE2 | HE2 | 850 | -7248.69 | 90.00 | 75.00 | 1.00 | 51.54 | 55.00 | -28.84 | 295.65 | -35.00 | -23.46 |
| HE3-1 | HE3-1 | 850 | -3203.67 | 90.00 | 55.57 | 0.33 | 50.00 | 51.54 | -17.02 | 221.48 | -38.46 | -5.57 |
| HE9 | COOLER6 | 1500 | -5187.05 | 50.00 | 11.10 | 0.10 | 10.01 | 10.17 | -10.76 | 321.46 | -39.83 | -1.09 |
| HE5-1 | HE5-1 | 50 | -3689.40 | 90.00 | 55.00 | 1.28 | 49.97 | 50.09 | -16.84 | 4381.72 | -39.91 | -5.03 |
| HE6-1 | HE6-1 | 50 | -1188.19 | 90.00 | 55.00 | 1.30 | 50.09 | 50.13 | -16.69 | 1423.41 | -39.87 | -4.91 |
| HE18 | COOLER5 | 1500 | -1597.89 | 90.00 | 11.05 | 0.33 | 9.97 | 10.01 | -18.35 | 58.06 | -79.99 | -1.08 |
| HE16 | HX-11 | 850 | 613.17 | 90.00 | 96.41 | 1.00 | 99.63 | 95.00 | 4.05 | 178.26 | 5.00 | 3.22 |
| HE15 | HX-AB | 850 | 25672.90 | 10.23 | 11.00 | 1.00 | 145.78 | 86.49 | 102.76 | 293.93 | 76.26 | 134.78 |
| HE7 | F-HX | 850 | -14544.30 | 10.04 | 10.00 | 1.00 | -0.45 | -0.45 | -10.47 | 1633.97 | -10.49 | -10.45 |
| HE3-3 | HE3C | 1500 | -57.38 | 10.60 | 10.00 | 1.00 | 5.00 | 8.00 | -3.67 | 10.42 | -2.60 | -5.00 |
| ignored | COOLER3 | 850 | -3.12 | 11.34 | 10.00 | 1.00 | 5.00 | 8.00 | -4.11 | 0.89 | -3.34 | -5.00 |
| HE5-2 | HE5-2 | 300 | -1060.91 | 55.00 | 11.30 | 1.28 | 10.17 | 10.20 | -11.87 | 297.97 | -44.80 | -1.13 |
| HE6-2 | HE6-2 | 300 | -891.04 | 55.00 | 11.30 | 1.30 | 10.20 | 10.23 | -11.78 | 252.14 | -44.77 | -1.10 |
| HE17 | HX-1 | 300 | 873.099 | 96.40 | 90 | 0.33 | 204.91 | 125.55 | 62.51 | 46.56 | 29.14 | 114.91 |
| HE12 | HI-REC | 850 | 4992.55 | 212.44 | 204.91 | 20.00 | 168.85 | 199.91 | -22.26 | 263.87 | -12.53 | -36.06 |
| HE19 | ECON | 850 | 6091.06 | 86.49 | 58.48 | 11.58 | -0.45 | 53.48 | -44.72 | 160.23 | -33.01 | -58.93 |

**Table S11.** Results details on the heat exchangers for the process alternative P3.

| **Heat exchanger** | **Name in Aspen plus** | **Heat transfer coefficient, W/m2C** | **Heat, kW** | **Tin, °C** | **Tout, °C** | **P, bar** | **Tutility in, °C** | **Tutility out, °C** | **LMDT, °C** | **Area, m2** | **dT1, C** | **dT2, C** |
| --- | --- | --- | --- | --- | --- | --- | --- | --- | --- | --- | --- | --- |
| HE13 | COOLER | 850 | -2085.73 | 204.99 | 11.01 | 20.00 | 10.01 | 10.14 | -36.78 | 66.71 | -194.86 | -1.00 |
| HE10 | MONO-JCK | 1500 | -259868.00 | 135.95 | 55.01 | 0.16 | 50.00 | 50.00 | -28.47 | 6084.90 | -85.95 | -5.01 |
| HE14 | COOLP | 850 | -1700.92 | 150.00 | 11.30 | 1.00 | 10.26 | 10.36 | -28.29 | 70.73 | -139.64 | -1.04 |
| HE7 | PR | 850 | 25170.00 | 11.38 | 50.00 | 1.00 | 55.01 | 16.39 | 5.01 | 5910.11 | 5.01 | 5.01 |
| HE11 | COOLER4 | 850 | -2437.43 | 16.39 | 10.90 | 0.16 | 9.86 | 10.01 | -2.95 | 973.53 | -6.38 | -1.04 |
| HE3-1 | HE3 | 850 | -7636.90 | 90.00 | 10.40 | 1.00 | 9.39 | 9.86 | -18.09 | 496.61 | -80.14 | -1.01 |
| HE4 | HE4 | 850 | -1994.82 | 20.00 | 11.20 | 1.00 | 10.14 | 10.26 | -3.92 | 599.03 | -9.74 | -1.06 |
| HE1 | HI-EL | 850 | 15839.00 | 10.00 | 63.00 | 1.00 | 90.00 | 20.00 | 17.11 | 1088.82 | 10.00 | 27.00 |
| HE2 | HE2 | 850 | -7248.69 | 90.00 | 75.00 | 1.00 | 50.00 | 61.61 | -26.66 | 319.87 | -28.39 | -25.00 |
| HE16 | COOLER2 | 850 | -9399.08 | 26.33 | 10.00 | 1.00 | 8.81 | 9.39 | -5.93 | 1866.21 | -16.94 | -1.19 |
| HE8 | COOLER6 | 850 | -361.86 | 10.92 | 10.00 | 1.00 | 8.79 | 8.81 | -1.62 | 263.17 | -2.11 | -1.21 |
| HE5-1 | HE5-1 | 50 | -2962.50 | 90.00 | 67.00 | 1.28 | 61.61 | 66.34 | -12.35 | 4796.40 | -23.66 | -5.39 |
| HE6-1 | HE6-1 | 50 | -184.58 | 90.00 | 71.00 | 1.30 | 66.34 | 66.63 | -11.60 | 318.18 | -23.37 | -4.66 |
| HE15 | COOLER7 | 1500 | -6700.35 | 50.00 | 41.09 | 0.08 | 10.59 | 11.00 | -34.57 | 129.20 | -39.00 | -30.50 |
| HE3-2 | HE3C | 850 | -39.23 | 10.40 | 10.00 | 1.00 | 5.00 | 8.00 | -3.54 | 13.03 | -2.40 | -5.00 |
| ignored | COOLER3 | 850 | -3.38 | 11.45 | 10.00 | 1.00 | 5.00 | 8.00 | -4.18 | 0.95 | -3.45 | -5.00 |
| HE9 | COOLER71 | 850 | -8120.51 | 41.09 | 11.00 | 0.08 | 5.00 | 8.00 | -15.86 | 602.20 | -33.09 | -6.00 |
| HE5-2 | HE5-2 | 300 | -1786.43 | 67.00 | 11.40 | 1.28 | 10.36 | 10.47 | -13.88 | 2574.53 | -56.53 | -1.04 |
| HE6-2 | HE6-2 | 300 | -1892.41 | 71.00 | 11.50 | 1.30 | 10.47 | 10.59 | -14.58 | 2596.76 | -60.41 | -1.03 |
| HE12 | HI-REC | 850 | 4991.56 | 212.44 | 204.99 | 20.00 | 168.85 | 200.00 | -22.22 | 264.25 | -12.44 | -36.14 |

**Table S12.** Results details on the heat exchangers for the process alternative P4.

| **Heat exchanger** | **Name in Aspen plus** | **Heat transfer coefficient, W/m2C** | **Heat, kW** | **Tin, °C** | **Tout, °C** | **P, bar** | **Tutility in, °C** | **Tutility out, °C** | **LMDT, °C** | **Area, m2** | **dT1, C** | **dT2, C** |
| --- | --- | --- | --- | --- | --- | --- | --- | --- | --- | --- | --- | --- |
| HE13 | COOLER | 850 | -5381.62 | 150.00 | 10.90 | 1.00 | 9.99 | 10.31 | -27.55 | 229.81 | -139.70 | -0.91 |
| HE10 | MONO-JCK | 1500 | -14981.50 | 102.36 | 55.00 | 0.16 | 51.07 | 50.00 | -18.71 | 533.76 | -52.36 | -3.94 |
| HE14 | COOLP | 850 | -1700.92 | 150.00 | 11.30 | 1.00 | 10.31 | 10.40 | -28.03 | 71.38 | -139.60 | -0.99 |
| HE7 | HI-HE-H | 1500 | -14861.50 | 31.23 | 11.01 | 0.01 | 9.25 | 10.00 | -7.82 | 1266.43 | -21.23 | -1.76 |
| HE15 | COOLER41 | 850 | -3131.76 | 46.11 | 46.11 | 0.10 | 10.82 | 11.00 | -35.20 | 104.68 | -35.11 | -35.29 |
| HE3-2 | HE3-2 | 850 | -4377.84 | 55.93 | 10.50 | 1.00 | 9.46 | 9.72 | -11.90 | 432.97 | -46.21 | -1.04 |
| HE4 | HE4 | 850 | -2063.02 | 20.00 | 10.90 | 1.00 | 9.87 | 9.99 | -3.94 | 615.65 | -10.00 | -1.03 |
| HE1 | HI-EL | 850 | 15839.00 | 10.00 | 63.00 | 1.00 | 90.00 | 20.00 | 17.11 | 1088.82 | 10.00 | 27.00 |
| HE2 | HE2 | 850 | -7248.69 | 90.00 | 75.00 | 1.00 | 51.56 | 55.00 | -28.84 | 295.73 | -35.00 | -23.44 |
| HE3-1 | HE3-1 | 850 | -3249.27 | 90.00 | 55.93 | 1.00 | 50.00 | 51.56 | -17.40 | 219.75 | -38.44 | -5.93 |
| HE8-1 | COOLER21 | 1500 | -2705.08 | 11.01 | 11.00 | 0.01 | 9.72 | 9.87 | -1.20 | 1497.08 | -1.13 | -1.28 |
| HE5-1 | HE5-1 | 50 | -3642.69 | 90.00 | 56.00 | 1.28 | 50.85 | 51.01 | -16.72 | 4356.87 | -38.99 | -5.15 |
| HE6-1 | HE6-1 | 50 | -1144.39 | 90.00 | 56.00 | 1.30 | 51.01 | 51.07 | -16.52 | 1385.46 | -38.93 | -4.99 |
| HE17 | COOLER7 | 850 | -1897.90 | 31.60 | 11.40 | 1.00 | 10.40 | 10.51 | -6.58 | 339.23 | -21.09 | -1.00 |
| HE12 | COOLER71 | 850 | -7000.00 | 104.58 | 31.60 | 1.00 | 37.09 | 110.31 | 5.61 | 1468.48 | 5.73 | 5.49 |
| HE16 | COOLER8 | 850 | -2410.89 | 155.00 | 37.00 | 5.43 | 10.68 | 10.82 | -69.30 | 40.93 | -144.18 | -26.32 |
| HE11 | COOLER9 | 50 | -23448.20 | 99.39 | 55.00 | 1.00 | 49.97 | 50.85 | -19.19 | 1437.30 | -48.54 | -5.03 |
| HE18 | COND | 50 | -1787.07 | 55.00 | 11.50 | 1.00 | 10.58 | 10.68 | -11.21 | 3189.31 | -44.32 | -0.92 |
| Combustion chamber | STHCL | 50 | -3401.01 | 2250.00 | 2250.00 | 1.01 | 37.00 | 344.96 | -2055.17 | 33.10 | -1905.04 | -2213.00 |
| Anhydrate dryer | DEHYDR | 850 | 7990.12 | 50.00 | 150.00 | 1.00 | 184.69 | 155.00 | 63.49 | 148.07 | 105.00 | 34.69 |
| HE3-3 | HE3C | 850 | -49.03 | 10.50 | 10.00 | 1.00 | 5.00 | 8.00 | -3.61 | 15.99 | -2.50 | -5.00 |
| HE8-2 | COOLER2 | 850 | -13621.00 | 11.00 | 10.00 | 0.01 | 5.00 | 8.00 | -3.91 | 4095.86 | -3.00 | -5.00 |
| HE9 | COOLER4 | 850 | -27946.50 | 46.11 | 10.00 | 0.10 | 5.00 | 8.00 | -16.30 | 2016.85 | -38.11 | -5.00 |
| HE5-2 | HE5-2 | 50 | -1104.86 | 56.00 | 11.50 | 1.28 | 10.51 | 10.58 | -11.60 | 1904.53 | -45.42 | -0.99 |
| HE6-2 | HE6-2 | 50 | -931.57 | 56.00 | 11.60 | 1.30 | 5.00 | 8.00 | -20.87 | 892.93 | -48.00 | -6.60 |

**Table S13.** Results details on the absorber for the studied CODA process alternatives.

| **Process alternative** | **P1** | **P2** | **P3** | **P4** |
| --- | --- | --- | --- | --- |
| CO2 captured, kg/h | 17301.3 | 17301.3 | 17301.2 | 17301.2 |
| Average cap rate, kg CO2/h/m3 | 0.362749 | 0.989368 | 0.362749 | 0.362749 |
| Total volume, m3 | 50583.3 | 17487.2 | 54178 | 50583.1 |
| Gm/Lm | 2.15659 | 0.0674749 | 2.1626 | 2.13906 |
| Number of nozzles NP841 |  | 5.48E+06 |  |  |
| Number of holes |  | 4.61E+09 |  |  |
| Depth, m | 9.1208 | 66.4971 | 9.769 | 9.1208 |
| Height, m | 20 | 5 | 20 | 20 |
| Lenght of box or diameter, m | 277.296 | 73.4044 | 277.295 | 277.295 |
| Air inlet area, m2 | 5545.92 |  | 5545.91 | 5545.91 |
| Pump energy consumption, kW | 1134.03 | 19176.1 | 1131.3 | 1143.33 |
| Pump energy due to height, kW | 1134.03 | 15947.2 | 1131.3 | 1143.33 |
| Energy due to nozzle or packing, kW | 0 | 3228.93 | 0 | 0 |
| Nozzle or packing energy fraction | 0 | 0.168383 | 0 | 0 |
| Fan energy consumption, kW | 2251.38 | 0 | 2411.29 | 2251.2 |
| Total energy consumption, kW | 3385.42 | 19176.1 | 3542.59 | 3394.54 |

**References**

Flagiello, Domenico, Arianna Parisi, Amedeo Lancia, and Francesco Di Natale. 2021. “A Review on Gas-Liquid Mass Transfer Coefficients in Packed-Bed Columns.” *ChemEngineering* 5(3). doi: 10.3390/chemengineering5030043.

Ghaffari, Somayyeh, Maria F. Gutierrez, Peter Schulze, Andreas Seidel-Morgenstern, and Heike Lorenz. 2025. “Solubility and Metastable Zone Width Measurement of Na2CO3 Hydrate Phases in the Na2CO3-NaOH-H2O System as a Basis for a Novel Carbon- Negative Soda Ash Production Strategy.” *Industrial & Engineering Chemistry Research* (ie-2025-00320a). doi: https://doi.org/10.1021/acs.iecr.5c00320.

Ghaffari, Somayyeh, Maria F. Gutierrez, Andreas Seidel-Morgenstern, Heike Lorenz, and Peter Schulze. 2023. “Sodium Hydroxide-Based CO2 Direct Air Capture for Soda Ash Production─Fundamentals for Process Engineering.” *Industrial & Engineering Chemistry Research* 62(19):7566–79. doi: 10.1021/acs.iecr.3c00357.

Holmes, Geoffrey, and David W. Keith. 2012. “An Air-Liquid Contactor for Large-Scale Capture of CO2 from Air.” *Philosophical Transactions of the Royal Society A: Mathematical, Physical and Engineering Sciences* 370(1974):4380–4403. doi: 10.1098/rsta.2012.0137.

Ramdin, Mahinder, Andrew R. T. Morrison, Mariette De Groen, Rien Van Haperen, Robert De Kler, Erdem Irtem, Antero T. Laitinen, Leo J. P. Van Den Broeke, Tom Breugelmans, J. P. Martin Trusler, Wiebren De Jong, and Thijs J. H. Vlugt. 2019. “High-Pressure Electrochemical Reduction of CO2 to Formic Acid/Formate: Effect of PH on the Downstream Separation Process and Economics.” *Industrial and Engineering Chemistry Research* 58(51):22718–40. doi: 10.1021/acs.iecr.9b03970.

Seider, Warren D., J. D. Seader, and Daniel R. Lewin. 2004. *Product and Process Design Principles: Synthesis Analysis and Evaluation*. Second Edition. John Wiley & Sons, Inc.

Treybal, Robert E. 1980. *Mass-Transfer Operations*. Third Edition. McGraw-Hill.
